# Multilayer brain network analysis in mice reveals ketamine-induced reorganization of brain- wide fluctuations and gut-brain axis

**DOI:** 10.1038/s42003-026-10606-0

**Published:** 2026-07-03

**Authors:** Fengkai He, Xiaojun Xu, Yuqi Zhu, Jiahui Lu, Jie Liu, Dongyong Guo, Aili Liu, Wenwen Bai, Xuyuan Zheng, Baolin Guo, Tiaotiao Liu

**Affiliations:** 1 School of Biomedical Engineering and Technology, Tianjin Medical University, Tianjin, China; 2 Bioland Laboratory, Guangzhou, China; 3 School of Medical Imaging, Tianjin Medical University, Tianjin, China; 4 Department of Critical Care Medicine, Tianjin Medical University General Hospital, Tianjin, China; 5 Department of Anesthesiology, Tianjin Medical University Cancer Institute and Hospital, Tianjin, China; 6 School of Basic Medicine, Tianjin Medical University, Tianjin, China; 7 Department of Neurobiology, School of Basic Medicine, Fourth Military Medical University, Xi’an, China

**Keywords:** Microbiology, Computational neuroscience, Psychiatric disorders

## Abstract

Depression involves dysregulation across distributed cortico-limbic circuits, and ketamine is notable for its rapid antidepressant effects. Although depression and ketamine treatment have been linked to altered brain network topology, how within-frequency and cross-frequency coupling are jointly reorganized at the brain-wide level remains unclear. Here, we developed a frequency-varying multilayer brain functional network (FMBFN) framework to analyze local field potential recordings from eight brain regions in male C57BL/6 mice. This framework integrates within- and cross-frequency coupling and extracts multi-scale network features to characterize brain network structure. Applying this approach in the chronic social defeat stress (CSDS) model, we found that CSDS was associated with frequency-specific hyperconnectivity and selective alterations in network integration during social interaction. Ketamine reversed social avoidance and induced the distinct reorganization of multilayer network topology, including region-specific nodal changes. Notably, the lateral habenula showed the response pattern opposite to that of the other recorded regions. As an exploratory cross-modal extension, we further examined gut microbial features and found that specific ketamine-associated microbial changes were linked to global network topology, suggesting candidate gut-brain association patterns. Together, these findings establish the FMBFN framework as a systems-level tool for characterizing brain-wide neural dynamics in psychiatric disorders and for linking network-level alterations to biological contexts.

## Abstract (web summary)

A frequency-varying multilayer brain network framework reveals stress-induced brain-wide network abnormalities and ketamine-associated functional reorganization underlying social avoidance in mice.

## Introduction

Depression, a common mental disorder affecting approximately 3.8% of the global population, is a leading contributor to the worldwide disease burden^1^. Chronic social stress is recognized as a major risk factor^2^, impairing social functioning—a core feature of depressive states^3^. The chronic social defeat stress (CSDS) model is widely used in preclinical depression research because it mimics the physical and psychological stressors encountered in humans^4^, reliably inducing social avoidance behavior, a hallmark of depression-like phenotypes^5^. Increasing evidence indicates that CSDS-induced social avoidance involves dysregulation within a distributed cortico-limbic network, with key nodes including the prefrontal cortex (PFC), nucleus accumbens (NAc), basolateral amygdala (BLA), ventral tegmental area (VTA), lateral habenula (LHb), and ventral hippocampus (vHPC)^6,7^.

Currently, pharmacological therapy remains one of the primary clinical strategies for treating depression. Among available antidepressant interventions, ketamine has received significant attention because of its rapid and sustained antidepressant effects after a single administration in both humans and rodents^8–13^. Evidence indicates that ketamine’s antidepressant actions also involve widespread cortical and limbic brain regions^14^. As our understanding of these regions grows, a crucial next step is to characterize neural activity holistically across these interacting circuits. A central challenge in this context is that depression-related and ketamine-related brain regions do not function independently, but rather as components of a complex, interconnected system. Capturing such system-level organization requires moving beyond region-specific analyses toward a comprehensive investigation of interregional connectivity^15^.

Network science has emerged as a useful tool for exploring interconnected neural systems^16^. While traditional single-layer brain functional networks have identified aberrant interaction patterns in social avoidance, they assume static operations within isolated frequency bands, neglecting the brain’s dynamic cross-frequency coordination across multiple spectral domains^17^. The frequency-varying multilayer brain functional network (FMBFN) overcomes the limitations of single-layer networks in capturing multi-dimensional neural information^18^. In the present study, we developed an FMBFN analytical framework based on multilayer network theory and applied it to depression-related research. Compared with traditional approaches, this framework integrates spectral and organizational dimensions to characterize neural activity at multiple scales, enabling a more nuanced and comprehensive characterization of brain-wide functional architecture.

Beyond central neural mechanisms, increasing evidence suggests that both the pathophysiology of depression and the actions of rapid-acting antidepressants involve the microbiota–gut–brain axis^19–21^. Specifically, this bidirectional system enables the brain to regulate gut motility and secretion via autonomic signaling^22^, while gut status may in turn influence brain activity through neural pathways and microbially derived metabolites^23,24^. Given accumulating evidence that both CSDS and ketamine can alter gut microbial composition^25–27^, the present study incorporated gut-microbial features as an exploratory cross-modal extension to examine whether FMBFN-derived neural network alterations are associated with changes in the gut microbiota.

To demonstrate the utility of this framework, we combined local field potentials (LFPs) recordings from eight cortico-limbic regions with 16S rRNA sequencing of gut microbiota. Specifically, the framework was used to (1) characterize CSDS-induced brain-wide alterations associated with social avoidance, (2) quantify ketamine-related reorganization of network dynamics during behavioral rescue, and (3) explore gut–brain axis-related associations between neural network alterations and gut-microbial changes. By transforming complex neural data into interpretable, system-level features, the FMBFN framework provides a structured approach for characterizing brain-wide functional architecture. This framework offers mechanistic insights into psychiatric disorders and may inform future therapeutic strategies.

## Results

### The frequency-varying multilayer brain functional network (FMBFN) analytical framework

Our FMBFN analytical framework comprises the following integrated components:

1. Multilayer network construction. The FMBFN’s complete construction pipeline is illustrated in Fig. 1, proceeding through four key stages: (1) LFPs were recorded from eight brain regions (using a custom multi-region electrode array, Supplementary Fig. 1) during social interaction tests (SIT). The signals were segmented into discrete trials, with each trial defined as the period from entry to exit from the social interaction zone. Multiple trials were obtained from each mouse during the SIT session. Because trial duration varied substantially depending on how long each mouse remained within the zone, each trial was further divided into non-overlapping 5-s epochs to improve analytical consistency. Each epoch was then used as the basic unit for constructing a single FMBFN (Fig. 1A, B); (2) For each epoch, Cleaned LFPs were filtered into five distinct frequency bands: band Ⅰ (2–7 Hz), band Ⅱ (7–12 Hz), band Ⅲ (12–30 Hz), band Ⅳ (30–60 Hz), and band Ⅴ (60–90 Hz), for the subsequent construction of the FMBFN (Fig. 1C); (3) Functional connectivity strength was quantified using the phase-lag index (PLI) for all within- and cross-frequency combinations. Specifically, the within-frequency functional connectivity (WFC) matrices and cross-frequency functional connectivity (CFC) matrices characterized the functional connectivity strength between every pair of brain-region across frequency bands (Fig. 1D, E); (4) These connectivity matrices were then integrated into a generalized functional connectivity (GFC) matrix, which comprehensively captured the multilayer architecture of neural interactions across frequencies by assembling the full set of WFC and CFC matrices(Fig. 1F); (5) To reduce spurious connections, a binarization threshold was applied to the GFC matrix to generate a super-adjacency matrix, which served as the mathematical representation of the FMBFN. Architecturally, the FMBFN consisted of five distinct layers, each corresponding to a specific frequency band: layer I (2–7 Hz), layer II (7–12 Hz), layer III (12–30 Hz), layer IV (30–60 Hz), and layer V (60–90 Hz). Within each layer, nodes representing brain regions were connected by edges denoting WFC, whereas interlayer connections linked the same node across different frequency bands and represented CFC. This multilayer organization enabled a unified representation of brain activity across the spectral domain (Fig. 1G, H).

**Fig. 1:**
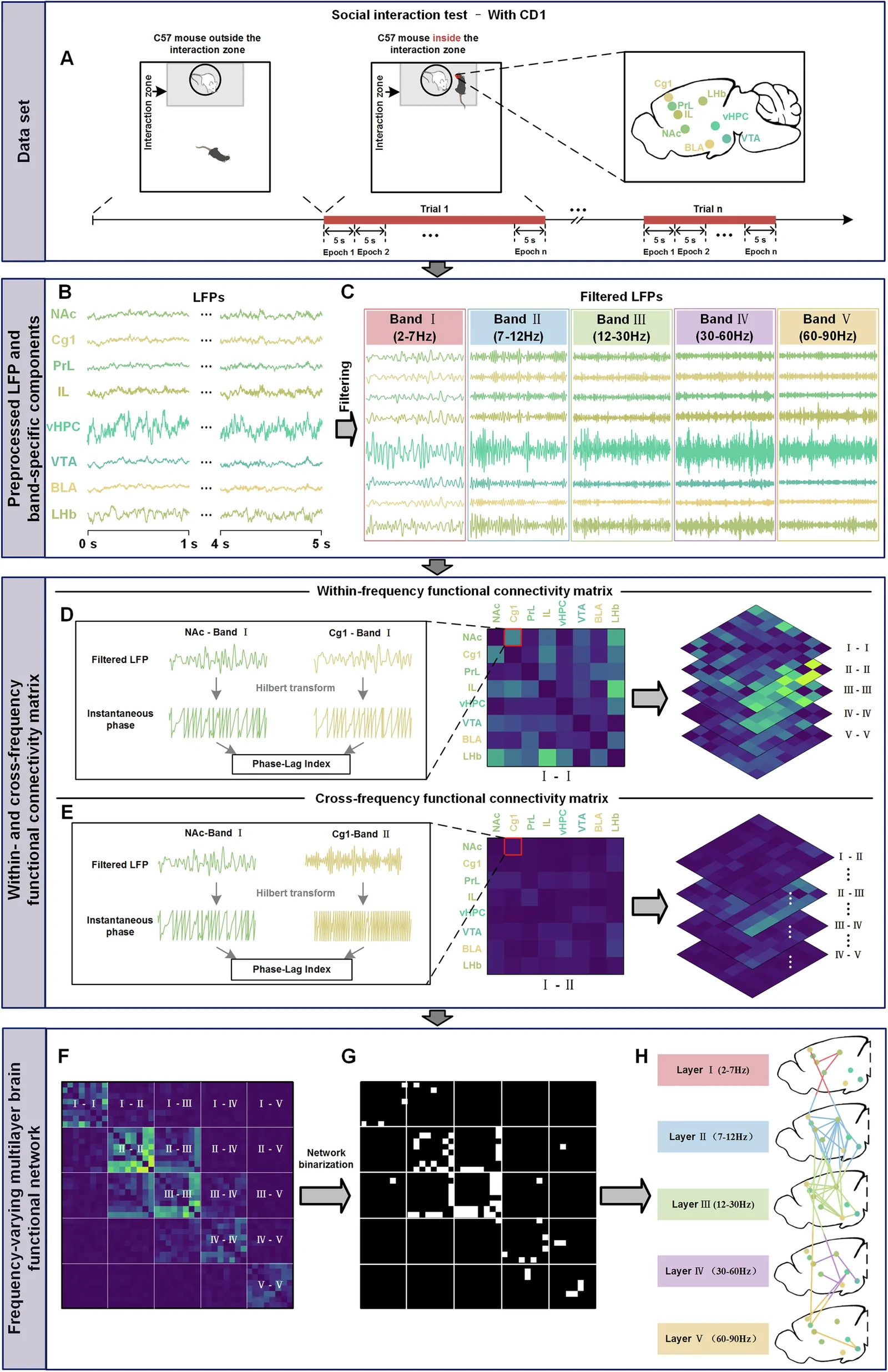
Workflow pipeline for constructing the frequency-varying multilayer brain functional network (FMBFN). **A** Schematic illustrating the protocol for Local field potentials (LFPs) acquisition and segmentation. The mouse illustrations were adapted from BioRender.com. **B** Sample 5-s LFP epochs. The panel displays cleaned LFP traces (duration = 5 s) concurrently recorded from eight brain regions. **C** Frequency band decomposition. signals were decomposed into five frequency bands. **D** Construction of the within-frequency functional connectivity (WFC) matrix set. The workflow for building a single WFC matrix is exemplified by the calculation between the NAc and Cg1 in band Ⅰ (Ⅰ–Ⅰ). **E** Construction of the cross-frequency functional connectivity (CFC) matrix set. The workflow for building a CFC matrix is illustrated by calculating the connectivity between the NAc (signal filtered in band Ⅰ) and the Cg1 (signal filtered in band Ⅱ) for the Ⅰ–Ⅱ matrix. **F** Schematic of the generalized functional connectivity (GFC) matrix. The GFC matrix provides an integrated representation of both WFC and CFC within a unified framework. **G** Schematic of the super-adjacency matrix. The super-adjacency matrix is derived by applying a binarization threshold to the GFC matrix. **H** Schematic of the FMBFN.
2. Multi-scale feature extraction. From the constructed FMBFN, we systematically extracted three complementary classes of features: (1) Functional connectivity strength: average coupling strength within and between all frequency bands; (2) Global topological properties: metrics that capture the overall integration and segregation of the network; (3) Local topological properties: node-specific measures that characterize regional roles in cross-frequency communication.
3. Computational validation via machine learning. To objectively evaluate the discriminative power and biological relevance of the extracted features, we employed a support vector machine classification approach. The features were used to distinguish between experimental groups in both binary and multiclass settings.
4. Multimodal data integration interface. The framework provides a flexible architecture for correlating neural features with external biological data. We implemented statistical tools for Spearman correlation and mediation analyses, enabling quantitative examination of the relationships between brain-wide fluctuations and peripheral systems such as the gut microbiome.

This integrated analytical framework not only enables the spectral characterization of neural interactions, but also their interpretation through topological analysis and validation via machine learning. Additionally, it facilitates contextual analysis through multi-modal integration, thereby establishing a framework applicable to systems-level neuroscience research.

### Characterizing CSDS-induced alterations in brain-wide functional architecture during social avoidance

**Fig. 2:**
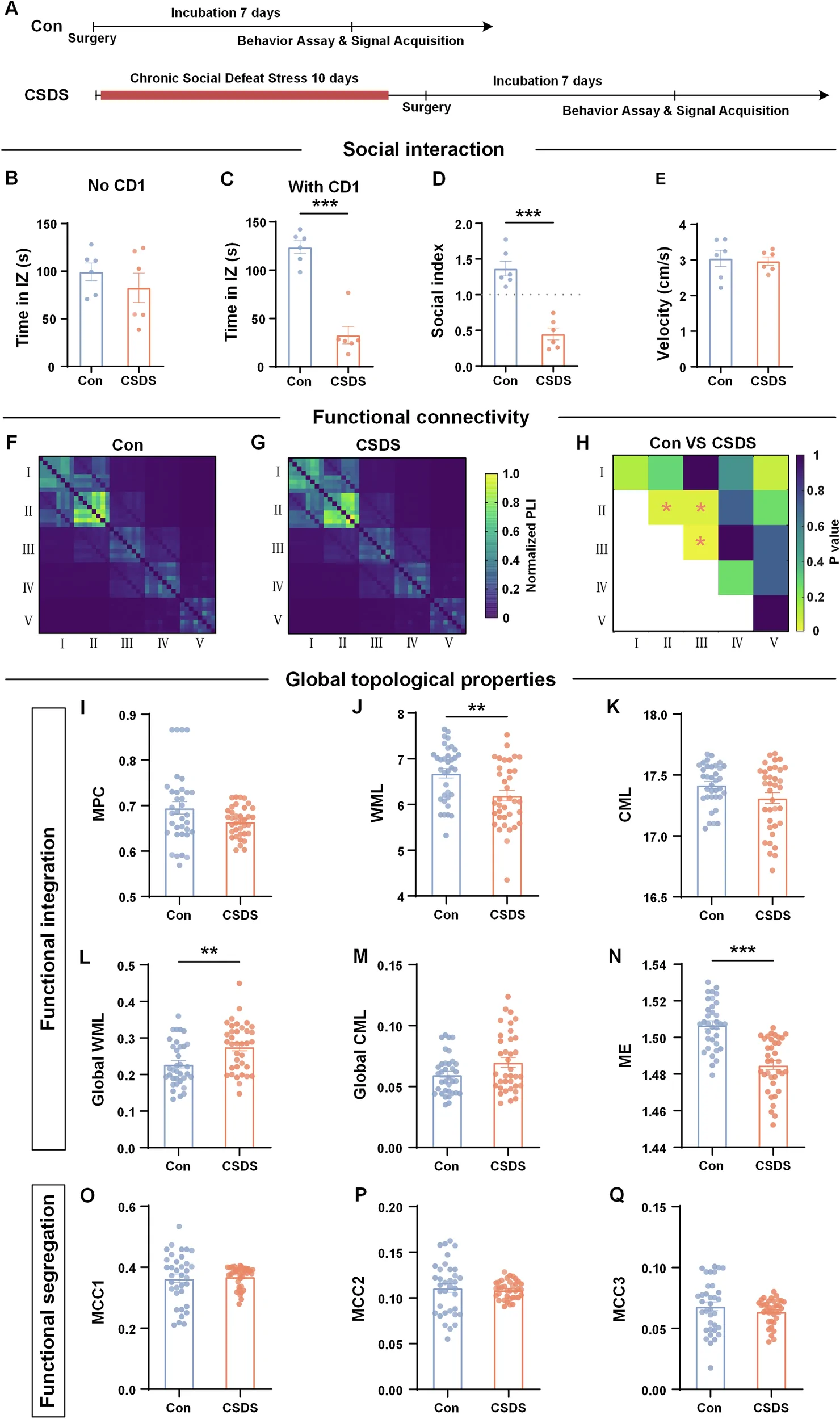
Multilayer network hyperconnectivity and topological disruptions underlie CSDS-induced social avoidance. **A** Experimental timelines for Control (Con) and chronic social defeat stress (CSDS) groups. **B** Time spent in the interaction zone by mice from the Con and CSDS groups during the “No CD1” phases of the SIT. **C** Time spent in the interaction zone by mice from the Con and CSDS groups during the “With CD1” phases of the SIT. **D** Social index of Con and CSDS mice. (E) Velocity of Con and CSDS mice in SIT. **B**–**E** All data are presented as mean ± SEM. For Con group: *n* = 6 mice; for CSDS group: *n* = 6 mice. Behavioral data from the full cohort of mice assigned to the electrophysiological study are provided in Supplementary Fig. 2. **F** The group-averaged GFC matrices are shown for the Con group Matrices, values represent the Normalized PLI. **G** Group-averaged GFC matrices for CSDS group. **H** Statistical comparison of functional connectivity strength between the Con and CSDS groups. Asterisks indicate significant differences (*P* < 0.05); orange and blue denote increased and decreased connectivity, respectively, in the CSDS group relative to Con. Inter-group differences were assessed using appropriate parametric or non-parametric tests (see Methods) with FDR correction for multiple comparisons. **I**–**Q** Comparison of the global topological properties. All data are presented as mean ± SEM, with each data point representing the average value across all epochs from a single mouse, all statistical comparisons were performed at the mouse level. For the Con group, *n* = 35 mice; for the CSDS group, *n* = 36 mice. All *P* values were corrected for multiple testing using the Benjamini–Hochberg false discovery rate (FDR) procedure. Detailed statistical methods and full statistical results are provided in Supplementary Data 1.

To evaluate the effects of CSDS on neurophysiological responses during social interaction, C57BL/6 mice were randomly assigned to two experimental groups: Con and CSDS (Fig. 2A). Following a post-surgical recovery period, LFPs were recorded from eight brain regions (NAc, Cg1, PrL, IL, vHPC, VTA, BLA, and LHb) during the SIT. To facilitate reading, a list of abbreviations used throughout the manuscript is provided in Supplementary Table 1.

#### CSDS results in social avoidance behavior

After exposure to CSDS, social avoidance behavior was assessed in male C57BL/6 mice using the SIT. During the “No CD1” phase, no significant difference was observed between CSDS group and Con group in the time spent in the interaction zone (Fig. 2B). In contrast, during the “With CD1” phase, CSDS mice spent significantly less time in the interaction zone compared to controls (Fig. 2C). Accordingly, the social interaction index (SI) was significantly reduced in the CSDS group (Fig. 2D), confirming the presence of social avoidance. Furthermore, no significant group differences were found in locomotor velocity during SIT (Fig. 2E), indicating that CSDS specifically impairs social behavior without affecting general locomotion.

#### CSDS-induced hyperconnectivity across specific frequency bands

Based on the recordings, we constructed FMBFNs and derived their corresponding GFC matrices for quantitative analysis. Only mice that exhibited social avoidance behavior (SI < 1) were included in the CSDS group, and behavioral data from the full cohort of mice assigned to the electrophysiological study are provided in Supplementary Fig. 2. The group-averaged matrices for the Con and CSDS groups are shown in Fig. 2F, G, respectively. Statistical analysis revealed a significant enhancement in within-frequency connectivity in the CSDS group, specifically in the II and III bands. Moreover, cross-frequency connectivity between bands II and III (II-III) was also significantly increased in the CSDS group compared to the Con group (Fig. 2H). These results demonstrate the capacity of the FMBFN analytical framework to detect specific frequency-band alterations in functional connectivity strength induced by CSDS, highlighting its sensitivity in resolving multidimensional neural disturbances.

#### CSDS selectively disrupts topological properties of FMBFN

Having established alterations in functional connectivity strength, we next investigated whether CSDS also disrupted the topological organization of the constructed multi-regional networks using the FMBFN analytical framework. Analysis of global topological properties revealed that CSDS preferentially affected metrics related to functional integration while sparing those of functional segregation (Fig. 2I–Q). Specifically, the Control group showed significantly higher within-frequency multilayer characteristic path length (WML) (Fig. 2J). Correspondingly, the CSDS group exhibited a significant increase in within-frequency multilayer global efficiency (Global WML) (Fig. 2L). Furthermore, multilayer entropy (ME) was markedly higher in the Control group compared to the CSDS group (Fig. 2N). Notably, these findings indicate that CSDS alters network integration without impairing segregation. To examine whether these topological effects could be attributed to differences in network sparsity, we quantified edge density for each within-frequency and cross-frequency matrix. No significant between-group differences in matrix-wise edge density were detected between the Con and CSDS groups (Supplementary Fig. 3A). We further performed a density-controlled analysis using matched block-specific edge densities across groups (Supplementary Fig. 4A). Under this condition, the group differences in WML and Global WML disappeared, whereas the difference in ME remained significant. These results indicate that path-length-related metrics were more sensitive to edge density, while the CSDS-related alteration in ME was robust after controlling for network sparsity, further supporting the conclusion that CSDS selectively disrupts functional integration rather than functional segregation.

To link global topological alterations with behavior, we conducted correlation analysis to investigate the relation between these metrics and the social index (Supplementary Fig. 5). WML, GlobalWML, and ME were significantly correlated with SI, consistent with the outcome of the statistical comparisons.

**Fig. 3:**
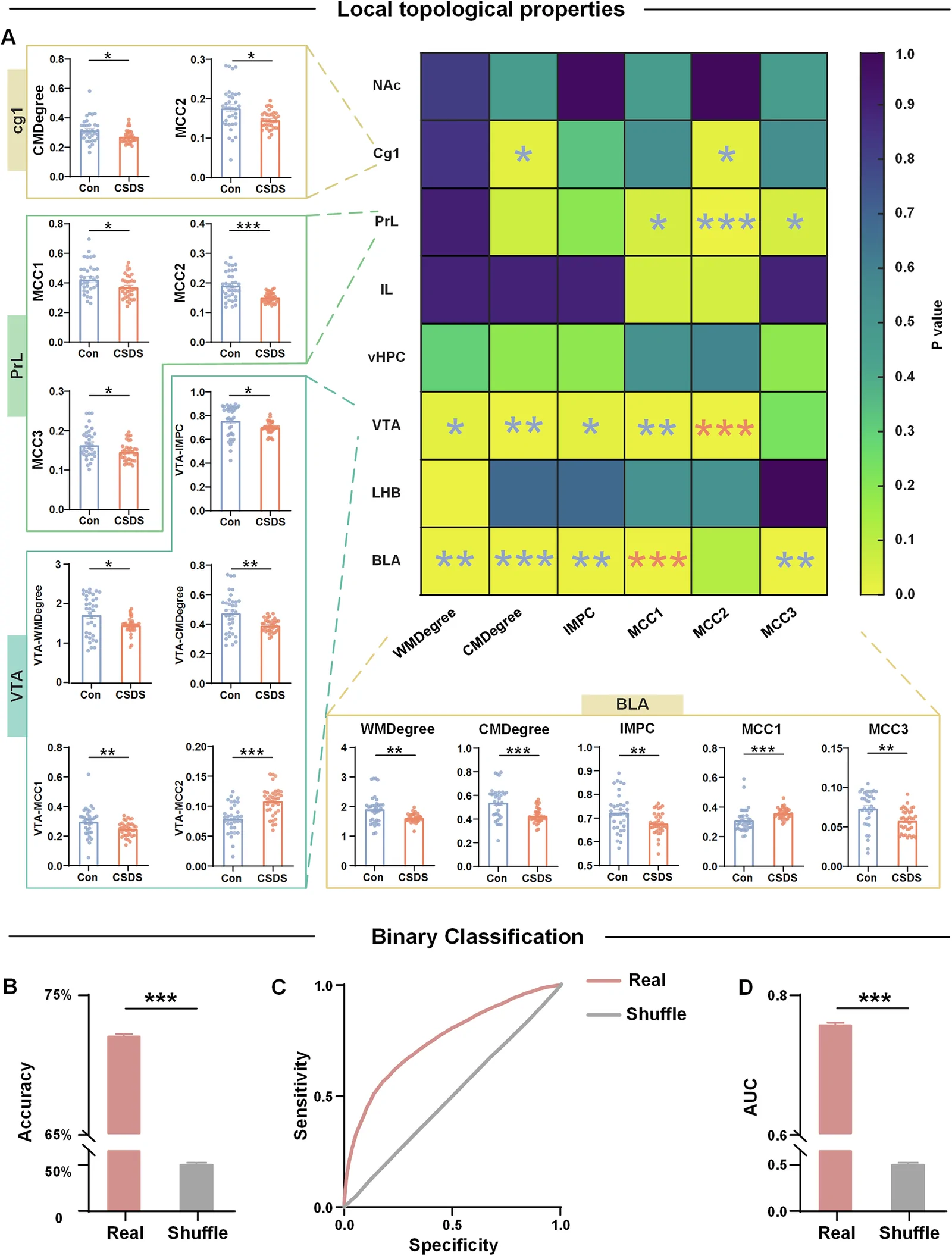
Local topological disruptions of the FMBFN and binary classification of Con and CSDS mice. **A** Heatmap summarizing the statistical comparison of local topological properties across brain regions. The color scale indicates *P* values. Asterisks mark significant group differences. *P < 0.05, **P < 0.01, *** *P* < 0.001. Representative bar plots are shown for properties with significant group differences. Significant differences are highlighted with asterisks, the color of the asterisks denotes the direction of the differences. Data are presented as mean ± SEM, all statistical comparisons were performed at the mouse level (Con, *n* = 35 mice; CSDS, *n* = 36 mice). **B** Binary classification accuracy based on functional connectivity strength and global topological properties for Real and Shuffle data. See “Methods” for details. Data are presented as mean ± SEM. (Mann-Whitney test; Real 72.12% ± 0.18%; Shuffle: 49.94 ± 0.26%; U = 50, ^***^ *P* < 0.001). **C** Average ROC from the binary classification using features derived from real data and shuffled data. **D** AUC values for distinguishing Con from CSDS groups using features from real data and from data with shuffled labels Data are presented as mean ± SEM. (Mann-Whitney test; Real 0.76 ± 0.00; Shuffle: 0.51 ± 0.01; *U* = 6, ^***^ *P* < 0.001). Detailed statistical methods and full statistical results are provided in Supplementary Data 1.

At the local network level, CSDS mice exhibited significant changes in multiple local topological properties across multiple brain regions (Fig. 3A). These disruptions suggest that CSDS impairs the capacity of key brain regions to integrate multi-frequency neural information, which may underlie the emergence of social avoidance.

Taken together, these results highlight the utility of the FMBFN analytical framework in capturing multidimensional neural disturbances induced by social defeat stress, revealing alterations across functional connectivity strength, global network topology, and local nodal properties.

#### Binary classification validates the discriminative power of FMBFN features

To further validate the biological relevance of the observed group differences, we next examined whether the FMBFN-derived features could reliably classify Con and CSDS mice using a binary SVM model.The binary SVM classification, designed to distinguish between the Con and CSDS groups based on the 24-dimensional FMBFN feature set (9 global topological properties, 15 connectivity features), yielded performance well above chance (shuffle). Across 100 iterations of training-testing splits, the classifier trained on the authentic data achieved a mean accuracy of 72.12% ± 0.18% (mean ± standard deviation), whereas the shuffle model, trained on data with randomly permuted class labels, performed at chance level (49.94% ± 0.26%). The difference in accuracy was statistically significant (Fig. 3B), confirming that the classification relied on biologically meaningful patterns rather than random correlations.

Receiver operating characteristic (ROC) analysis further validated the superiority of the real classifier. Its mean ROC curve consistently occupied the upper-left quadrant, indicating a robust balance of sensitivity and specificity across thresholds (Fig. 3C). By contrast, the shuffle ROC curve approximated the diagonal of random guessing. Quantitatively, the real classifier achieved an AUC of 0.76 ± 0.00, representing fair-to-good discriminative power, which was significantly higher than the shuffle AUC (0.51 ± 0.01) (Fig. 3D).

To further address the potential concern of within-mouse non-independence, we additionally repeated the classification analysis using animal-level splitting. Although the classification performance decreased compared with the epoch-level analysis, the animal-level classifier still remained significantly above the shuffled-label baseline in both accuracy and AUC (Supplementary Fig. 6).

Collectively, these results provide rigorous, data-driven validation that the brain-wide neural fluctuations characterized by our FMBFN analytical framework serve as sensitive and reliable biomarkers of CSDS-related neural signatures

### Quantifying ketamine-induced reorganization of Brain-wide fluctuations in the remediation of social avoidance

**Fig. 4:**
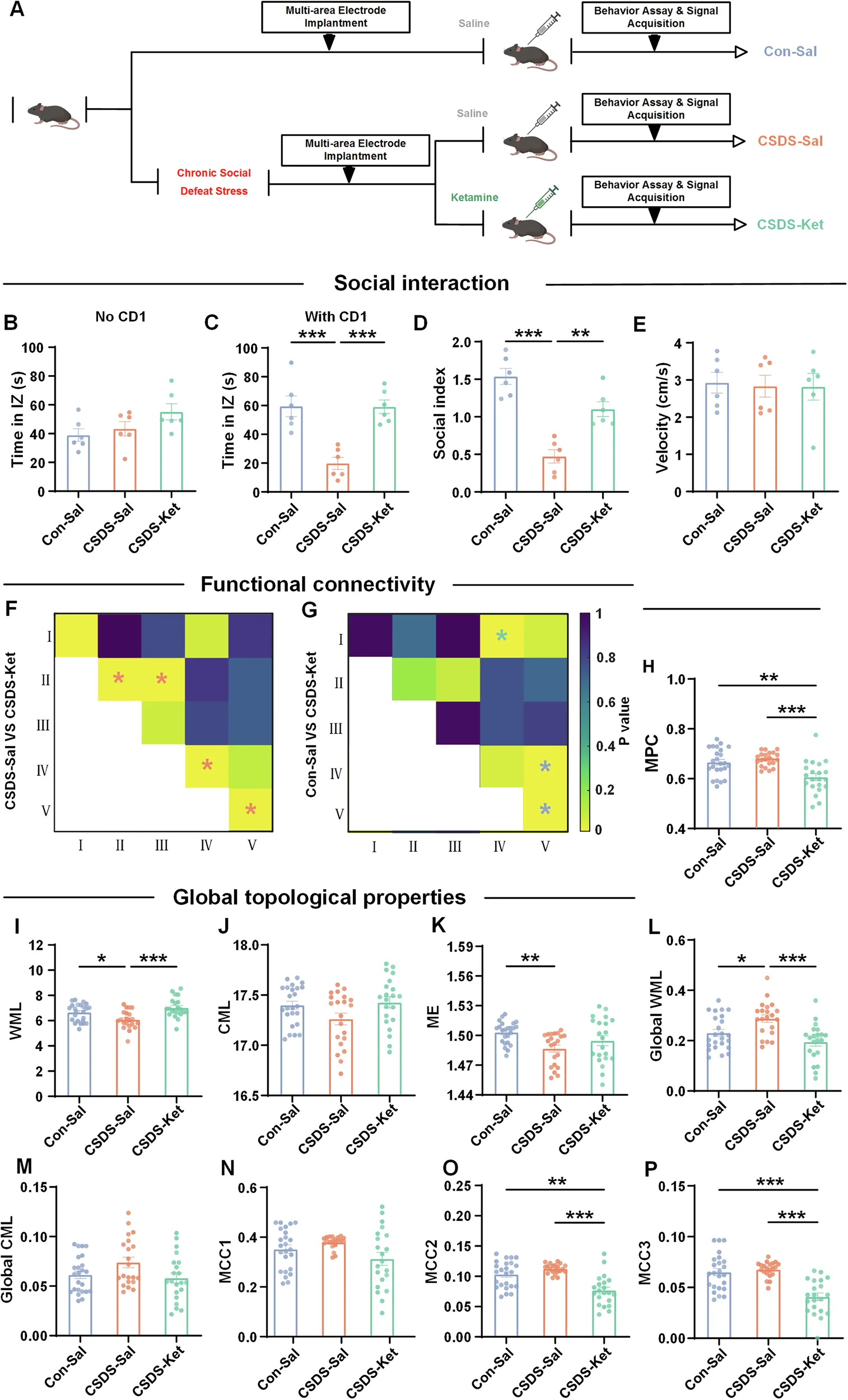
Ketamine alleviates social avoidance and induces a multilayer network reset. **A** Experimental design for behavioral tests and in-vivo electrophysiological signals acquisition after intraperitoneal injection of ketamine or saline, with the Control group (Con-Sal), CSDS group receiving saline (CSDS-Sal), and CSDS group receiving ketamine (CSDS-Ket). Mouse illustrations and syringe illustrations were adapted from BioRender.com. **B** Time spent in the interaction zone during the “No CD1” phases of the SIT for each group. **C** Time spent in the interaction zone during the “With CD1” phases of the SIT for each group. **D** SI of each group. **E** Velocity of each group in SIT. **B**–**E** All data are presented as mean ± SEM. For Con-Sal group: *n* = 6 mice; for CSDS-Sal group: *n* = 6 mice; for CSDS-Ket group: *n* = 6 mice. **F** Functional connectivity strength differences between CSDS-Sal and CSDS-Ket groups. Asterisks indicate significant differences (*P* < 0.05); green and orange denote increased and decreased connectivity, respectively, in the CSDS-Ket group relative to the CSDS-Sal group. **G** Functional connectivity strength differences between Con-Sal and CSDS-Ket groups. Green and blue denote increased and decreased connectivity, respectively, in the CSDS-Ket group relative to the Con-Sal group. **H**–**P** Comparison of the global topological properties. All data are presented as mean ± SEM, with each data point representing the average value across all epochs from a single mouse, all statistical comparisons were performed at the mouse level. For Con-Sal group: *n* = 24 mice; for CSDS-Sal group: *n* = 21 mice; for CSDS-Ket group: *n* = 21 mice. All *P* values were corrected for multiple testing using the Benjamini–Hochberg false discovery rate (FDR) procedure. Detailed statistical methods and full statistical results are provided in Supplementary Data 1.

We used the FMBFN analytical framework to examine the effects of ketamine in the CSDS model in order to assess its ability to identify neural changes by drug therapy. The control group received an intraperitoneal (i.p.) injection of saline (Con-Sal). Social defeat stress exposed mice were randomly assigned to two subgroups: one received a single i.p. injection of ketamine (10 mg/kg) and was labeled CSDS-Ket, while the other was administered an equivalent volume of saline (CSDS-Sal). Behavioral testing and LFP recordings were performed 24 h after injection (Fig. 4A).

#### Ketamine alleviates CSDS-induced social avoidance behavior

Consistent with ketamine’s known antidepressant properties, the CSDS-Ket group showed a significantly higher social index compared to CSDS-Sal mice, indicating a reversal of social avoidance behavior (Fig. 4B–D). No significant differences in locomotors activity were observed among groups, confirming that the behavioral improvement was not confounded by motor function (Fig. 4E).

#### Ketamine induces coordinated multilayer reorganization of network architecture

Given the behavioral rescue induced by ketamine, we next employed the FMBFN analytical framework to characterize its neuromodulatory effects. Our analysis revealed that ketamine induced a coherent reorganization encompassing both functional connectivity strength (Fig. 4F, G) and global topological properties (Fig. 4H–P).

Regarding functional connectivity strength, ketamine normalized CSDS-induced hyperconnectivity, specifically reversing the elevated within-frequency connectivity in band II and cross-frequency connectivity in band II-III observed in the CSDS-Sal group. Beyond this normalization, ketamine also elicited additional frequency-specific changes. Notably, the CSDS-Ket group showed significantly lower connectivity in bands IV and V compared to CSDS-Sal. Moreover, the connectivity profile of the CSDS-Ket group differed from that of the Con-Sal group, exhibiting reduced in bands V and IV-V, but enhanced in I-IV (Fig. 4F, G).

**Fig. 5:**
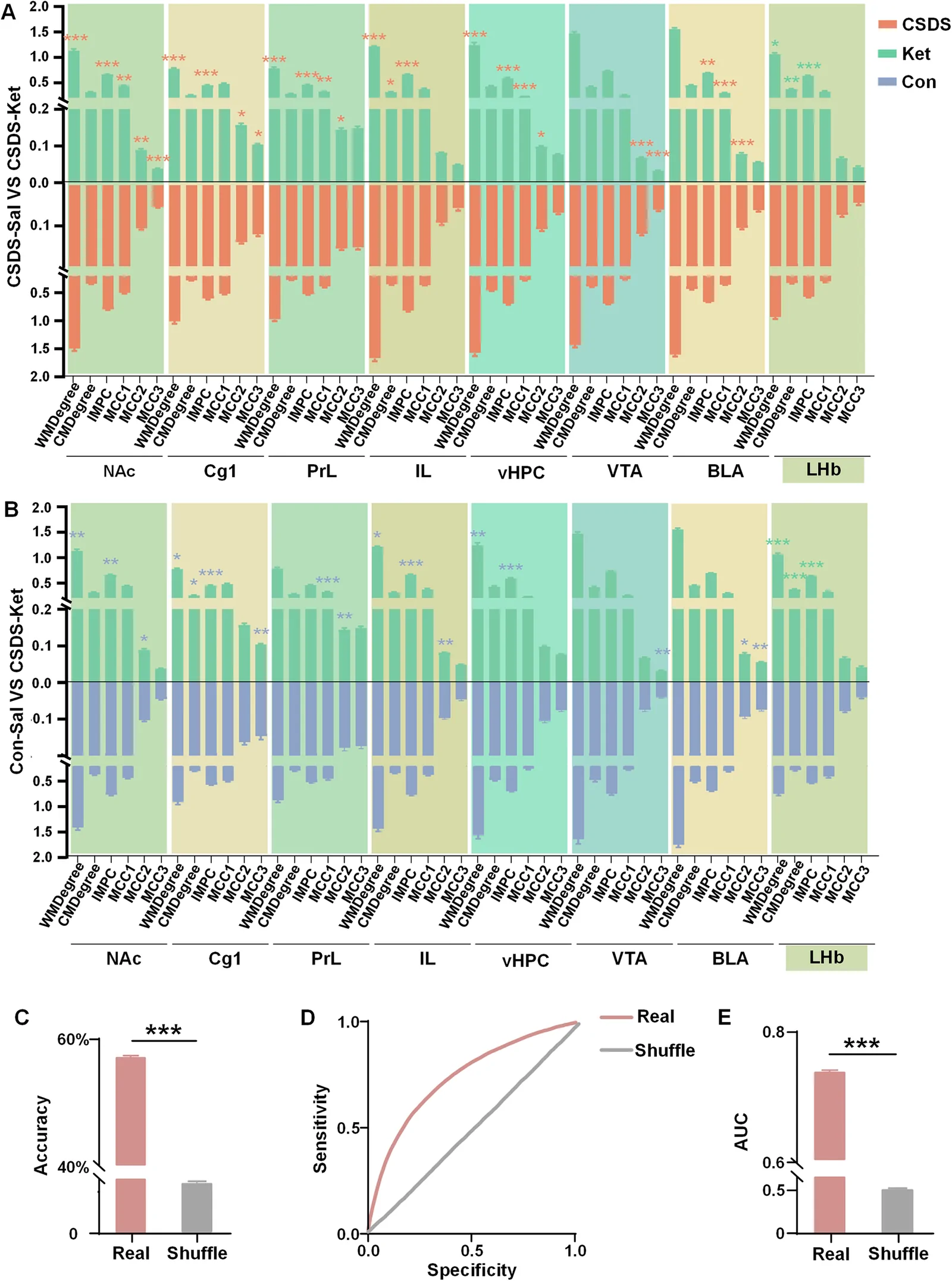
Ketamine induces region-dependent topological changes with opposite direction specifically in the LHb. **A** Region-specific comparisons of local multilayer topological properties between CSDS-Sal and CSDS-Ket groups. Bar chart display the results of pairwise comparisons between the CSDS-Sal and CSDS-Ket groups for local topological properties across all brain regions. **B** Region-specific comparisons of local multilayer topological properties between Con-Sal and CSDS-Ket groups. **A**, **B** All data are presented as mean ± SEM. Significant differences are highlighted with asterisks, the color of the asterisks denotes the direction of the differences. All statistical comparisons were performed at the mouse level. Detailed statistical methods and full statistical results are provided in Supplementary Data 1. **C** Three-class classification accuracy based on functional connectivity strength and global topological properties for Real and Shuffle data. See “Methods” for details (Welch’s t test; Real 57.15% ± 0.34%; Shuffle: 36.03% ± 0.76%; Welch-corrected *t* = 24.92, ^***^ *P* < 0.001). **D** Average ROC curves from the three-class classification using features derived from real data and shuffled data. **E** AUC values for three-class classification using features from real data and from data with shuffled labels (Mann-Whitney test; Real 0.73 ± 0.00; Shuffle: 0.50 ± 0.01; *U* = 17, ^***^ *P* < 0.001). All data are presented as mean ± SEM.

This dual pattern was mirrored in topological properties. Ketamine both normalized CSDS-induced deficits in functional integration—with MWL and globalMWL abnormalities in CSDS-Sal reversed to levels comparable to Con-Sal – and induced distinct alterations in topological metrics that were unaffected by CSDS alone (Fig. 4H–P). Metrics including MCC2, cross-three-frequency clustering coefficient (MCC3), and global MPC, which did not differ between Con-Sal and CSDS-Sal, were markedly reduced in CSDS-Ket relative to both other groups. For ME, which was also perturbed by CSDS, ketamine produced a non-significant trend toward normalization. Analysis of local topological properties revealed that ketamine induced widespread yet divergent changes across all eight brain regions examined (Fig. 5A, B). In each region, at least one local metric differed significantly in the CSDS-Ket group compared to the other two groups. Importantly, the direction of change was region-dependent: in the LHb, significantly altered local metrics were consistently higher in CSDS-Ket than in the other groups, whereas in all other regions, values were consistently lower in CSDS-Ket relative to both control groups. Notably, although matrix-wise edge density differed in several frequency matrices involving the CSDS-Ket group (Supplementary Fig. 3B), the main conclusion was preserved after density-controlled analysis: ketamine-related alterations remained evident in global topological dimensions that were not affected by CSDS alone, supporting the interpretation that ketamine induces an additional reorganization of multilayer network architecture rather than merely reversing the stress phenotype (Supplementary Fig. 4B).

Together, these results demonstrate that ketamine’s therapeutic effects involve coordinated reorganization across spectral, topological, and nodal properties, coherently captured by the FMBFN.

#### FMBFN features discriminate control, stressed, and ketamine-treated states

We next investigated whether the FMBFN features could differentiate the more complex neural states following ketamine treatment. In a three-way classification task (Con-Sal vs. CSDS-Sal vs. CSDS-Ket), the SVM achieved an accuracy of 57.15%, significantly above the shuffle performance (Fig. 5C). This discriminative capacity was further supported by a macro-average AUC of 0.73 (Fig. 5D, E). These results demonstrate that the FMBFN analytical framework captures system-level neural alterations that are not only statistically significant but also robust enough to allow a machine learning model to reliably distinguish the neural network signatures of health, chronic stress pathology, and ketamine-induced antidepressant.

### Elucidating gut-brain axis mediation mechanisms underlying ketamine’s therapeutic effects

**Fig. 6:**
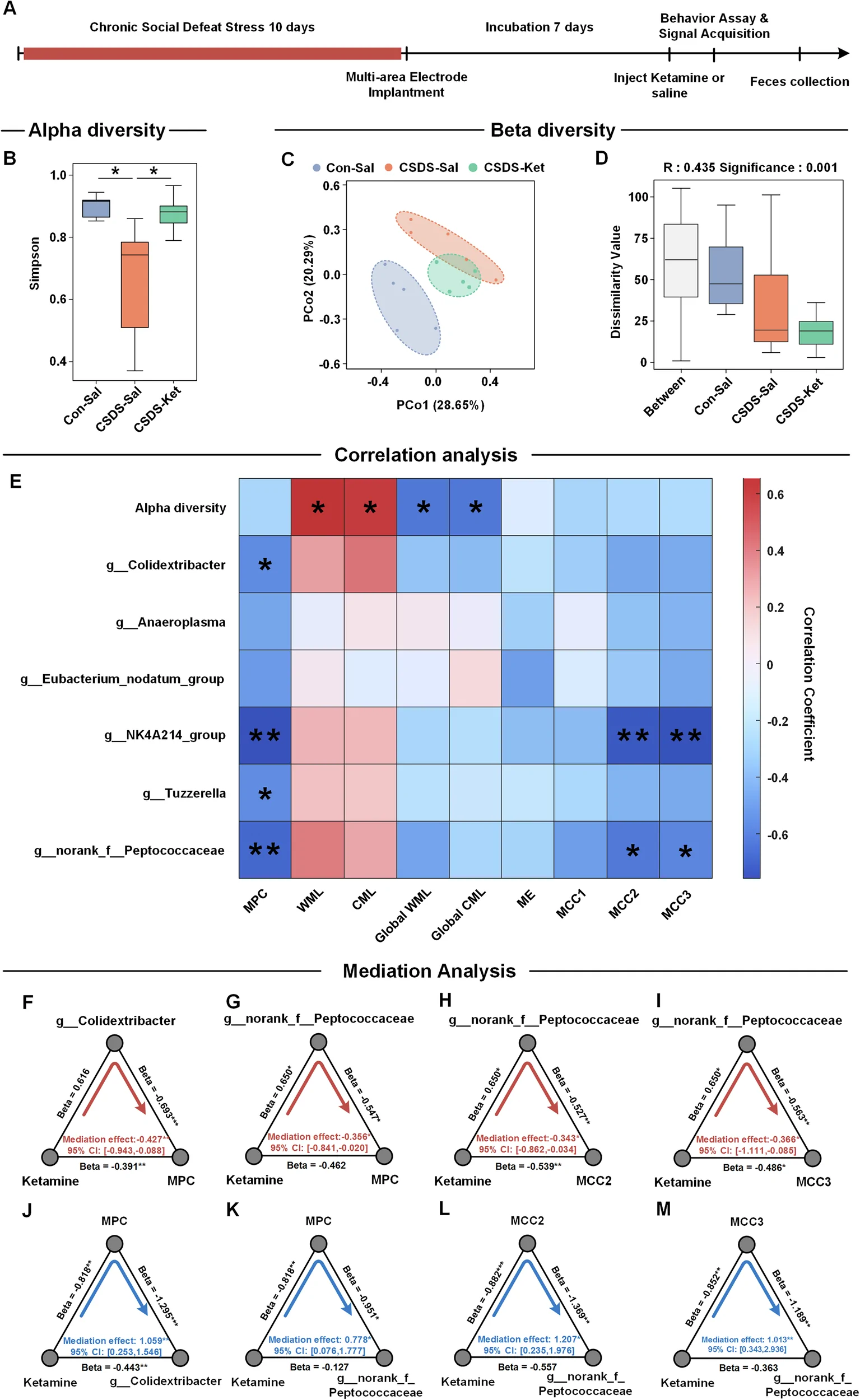
Ketamine regulates multilayer brain network remodeling and gut–brain association patterns. **A** Experimental design for multimodal data acquisition. **B** Comparison of microbial alpha-diversity. Simpson index values presented as box plots (median ± IQR) for Con-Sal, CSDS-Sal, and CSDS-Ket groups **C** Beta diversity analysis of gut microbiota. Principal coordinates analysis (PCoA) plot illustrating beta diversity across groups using Bray-Curtis distance. **D** Analysis of similarities (ANOSIM) among the three groups. In the PCoA plot, each point represents one sample. In the ANOSIM plot, the *x*-axis shows groups and the y-axis shows ranked sample distances. A positive *R* value indicates greater between-group than within-group dissimilarity. Box plots show the median and interquartile range (IQR); whiskers indicate the minimum and maximum values. **E** Correlation between microbial features and global topological properties. Tile plot displays Spearman correlation coefficients between gut microbial features and global topological properties. Red and blue tiles represent positive and negative correlations, respectively. Significance levels after FDR correction are indicated by asterisks (^*^ *P* < 0.05, ^**^ *P* < 0.01^, ***^ *P* < 0.001).

To examine whether FMBFN-derived neural network alterations are associated with changes in the gut microbiota, we performed 16S rRNA gene sequencing of colonic content samples collected 24 h after ketamine injection (immediately prior to LFP recordings) from all three experimental groups. This approach enabled simultaneous evaluation of gut microbiota alterations following CSDS and ketamine treatment (Fig. 6A).

#### Ketamine administration reverses CSDS-induced gut microbial dysbiosis

Analysis of gut microbiota alpha diversity revealed that the CSDS-Sal group exhibited a significantly lower Simpson index compared to both the Con-Sal and CSDS-Ket groups. No significant difference was observed between the Con-Sal and CSDS-Ket groups, indicating that CSDS significantly reduced microbial community richness, an effect reversed by ketamine treatment (Fig. 6B).

Beta diversity, assessed through PCoA based on Bray-Curtis dissimilarity (Fig. 6C), demonstrated distinct clustering of the gut microbiota among the three groups. Statistical testing of intergroup differences confirmed significant separation (Fig. 6D), suggesting that CSDS markedly altered the microbial community structure, while ketamine modulated gut microbiota composition in CSDS mice. This structural reorganization was further reflected in taxonomic composition, from phylum to family (Supplementary Fig. 7).

#### Identification of genus-level microbial biomarkers for ketamine intervention

We next aimed to identify potential microbial biomarkers associated with ketamine intervention at genus level, we performed LEfSe analysis followed by Kruskal-Wallis rank-sum test analyses. LEfSe analysis revealed 11 differentially abundant bacterial genera (LDA score >2) (Supplementary Fig. 8A), which were further subjected to Kruskal–Wallis tests with post hoc pairwise comparisons (Supplementary Fig. 8B–L). A genus was considered a potential biomarker if it met either of the following two criteria^26^:

1. Reversion-type: Significantly different between CSDS-Sal and Con-Sal groups, and this difference significantly reversed in CSDS-Ket.

2. Enrichment-type: Significantly higher abundance in CSDS-Ket compared to both other groups, with no significant difference between Con-Sal and CSDS-Sal.

Based on these criteria, six genera were identified as potential biomarkers: *g_ Colidextribacter*, *g_Anaeroplasma*, *g_Eubacterium_nodatum_group*, *g_NK4A214_group*, *g_Tuzzerella* and *g_norank_f__Peptococcaceae*. Notably, all six were classified as enrichment-type (Supplementary Fig. 8B-G).

#### FMBFN analytical framework reveals bidirectional gut-brain communication via multimodal data integration

To explore gut–brain axis-related associations linked to ketamine treatment using our FMBFN analytical framework, we first performed correlation analyses between microbial features (alpha diversity and identified potential microbial biomarkers) and global topological properties of the FMBFN (Fig. 6E). Alpha diversity showed significant positive correlations with WML and cross-frequency multilayer characteristic path length (CML), but negative correlations with globalWML and cross-frequency multilayer global efficiency (globalCML). In addition, the identified microbial biomarkers were significantly associated with global topological properties: *g_Colidextribacter* and *g_Tuzzerella* abundances were negatively correlated with MPC, while *g_NK4A214_group* and *g_norank_f__Peptococcaceae* exhibited significant negative correlations with MPC, MCC2 and MCC3.

To further evaluate these complex relationships, we conducted the bidirectional mediation analysis (Fig. 6F–M). Unlike correlation analysis, mediation analysis not only reveals the correlation among variables, but also provides potential causal insights. Strikingly, significant bidirectional mediation effects were observed between *g_Colidextribacter* and MPC; *g_norank_f__Peptococcaceae* and MPC, MCC2, MCC3. These findings suggest that ketamine-induced topological network reorganization may be partly mediated by specific gut microbial features through the gut–brain axis. Conversely, ketamine-associated alterations in gut microbiota may also be linked to changes in brain network topology through the pathway. These candidate bidirectional associations warrant further experimental validation.

Collectively, these findings illustrate the utility of the FMBFN analytical framework for integrating multimodal biological data and identifying candidate gut–brain interaction patterns associated with ketamine treatment.

## Discussion

We present an FMBFN framework that characterizes brain-wide neural fluctuations and topological features from in vivo LFP recordings and 16S RNA sequencing of gut microbiota. In contrast to conventional analyses, this framework integrates multiple dimensions to capture system-level dynamics. We applied it to three scenarios: (i) detecting CSDS-induced neural disturbances, (ii) characterizing ketamine-induced reorganization, and (iii) examining gut-brain axis interactions. Our results reveal that CSDS pathology was associated with frequency - specific hyperconnectivity alongside selective disruption of global integrative topology. In contrast, ketamine exerts its effects through a multilayer “network reset”, characterized by simplification of global organization and region-specific reprogramming—notably enhancing LHb centrality while suppressing influence in other cortico-limbic regions. To assess the translational relevance of the FMBFN-derived features, SVM classification achieved high accuracy in distinguishing stress states, indicating the framework’s sensitivity in capturing pathophysiological network signatures. Furthermore, by integrating multimodal data uncovered potential bidirectional mediation between gut microbiota and brain network topologies enabled by the core strengths of our FMBFN analytical framework.

A central feature of our FMBFN analytical framework is its ability comprehensively and holistically characterize of neural activity. This advantage manifests across two critical dimensions: the spectral dimension and the interpretative dimension. In the spectral dimension, traditional single-layer brain network models often neglect cross-frequency coupling (CFC), a critical mechanism for neural coordination across spatial and temporal scales^28^. Notably, CFC disruptions have been implicated in several psychiatric disorders, including depression and Alzheimer’s disease, where aberrant theta-gamma coupling serves as a potential biomarker^29,30^. By explicitly incorporating both within-frequency coupling (WFC) and CFC, our framework offers an expanded representation of neurophysiological interactions. This integrated approach enabled us to uncover pathological features in the CSDS model, including hyperconnectivity within Bands II (7-12 Hz) and III (12-30 Hz) and abnormal coupling between these bands. In the interpretative dimension, functional connectivity strength quantifies synchronization between neural signals across brain regions over frequency, bridging cellular activity with large-scale brain function and illuminating how coordinated neural communication supports cognition and behavior^31^. Meanwhile, topological properties extend beyond pairwise connections, revealing the brain’s higher-order organizational principles for efficient information processing. Our comprehensive topological analysis incorporated both global metrics, reflecting overall network integrity, and local metrics, identifying specific disruption sites. This approach was essential because local analysis uncovered a pronounced MCC2 reduction specifically in the PrL, a finding obscured by network-wide averages. Our FMBFN framework integrates functional connectivity strength, global topology, and local topology into a unified analytical paradigm, providing a systems-level mechanistic explanation addresses the limitations inherent to single-perspective analyses. Additionally, the framework incorporates an SVM-based machine learning validation component, providing additional computational support of the biological relevance and discriminative power of the extracted neural features. The SVM classification demonstrated that the multiscale features, integrating spectral and topological information, effectively distinguish between experimental groups with high accuracy. This computational validation strengthens the biological significance of our findings and positions the FMBFN framework as a powerful tool for generating interpretable biomarkers of brain states. Ultimately, the FMBFN framework enables the characterization of large-scale neural dynamics from multiple perspectives, providing a systematic tool for understanding neural circuitry and linking neural features to broader biological contexts.

Our analysis revealed that CSDS induced significant changes in global topological properties, including decreased WML, increased globalWML, and reduced ME. These alterations suggest a state of hyper-integration and reduced network flexibility under stress, in which the brain’s ability to balance segregation and integration is impaired. The brain operates as a complex network that balances functional segregation (specialized processing) and integration (global communication) in a small-world architecture. This architecture is crucial for efficient neural computation^32^. Small-world networks combine the benefits of both random and regular networks, optimizing both local specialization and global efficiency^33^. The observed global hypersynchronization is likely driven by exaggerated synchronization within specific frequency bands, such as Bands II and III, as seen in our functional connectivity strength analysis. This hyperconnectivity within bands shortens information transmission paths (lower WML) and increases global synchronization (higher globalWML), shifting the brain’s network from an optimal small-world state to a rigid, inefficient one. This shift mirrors what is seen in major depressive disorder (MDD), where neuroimaging studies report similar disruptions in network balance. In treatment- naïve MDD patients, structural networks show reduced shortest path length and increased global efficiency, reflecting a breakdown in the balance between local and global network functions^34^. Similarly, functional networks in drug- naïve, first-episode MDD patients exhibit decreased characteristic path length and increased global efficiency, indicating network randomization^35^. Notably, Li et al. showed that electroconvulsive therapy (ECT) reverses this hyper-integration by restoring path length and reducing global efficiency, improving depressive symptoms^36^. Furthermore, changes in ME, which reflects the capacity for functional integration, highlight a loss of network diversity and adaptability that exacerbates the hyper-integrated state. Oscillatory signals contain both phase and amplitude components^37^, and within- and cross-frequency interactions can be characterized from multiple complementary perspectives, including phase–phase (PPC), amplitude–amplitude(AAC), phase–amplitude(PAC), and time-dependent coupling^28^. Different metrics therefore capture different aspects of neural coordination rather than interchangeable forms of the same phenomenon^38^. In our study, both within-frequency and cross-frequency interactions were quantified using PLI, a phase-based PPC metric. A change in cross-frequency PLI indicates that the temporal stability of non-zero phase relationships between oscillatory activities operating at different timescales has been altered across distributed brain regions. One plausible biological interpretation is that oscillatory phase alignment may help regulate inter-areal communication by aligning windows of neuronal excitability^17,39,40^; thus, altered cross-frequency PLI may reflect altered temporal coordination of neuronal communication across timescales. In this framework, the increased II–III cross-frequency PLI observed in the CSDS group may indicate abnormally rigid cross-timescale locking or excessive synchronization, consistent with the concomitant increase in global integration and reduced network flexibility. Conversely, ketamine-related reductions in cross-frequency PLI may reflect a release from this rigid temporal locking and a shift toward more flexible network communication. Because different CFC metrics index distinct physiological processes, our PLI-based phase measure should be considered complementary to PAC- and AAC-based measures rather than interchangeable with them.

Nowadays, pharmacological interventions remain the primary treatment for depression^41,42^. Ketamine, a non-competitive N-methyl-D-aspartate receptor (NMDAR) antagonist, blocks the ion permeation pore located inside the NMDAR. Our findings demonstrate that ketamine induces a fundamental reorganization of brain network architecture, characterized by reduced MPC, MCC2, and MCC3. This pattern suggests a broad reduction in network complexity that impairs both integrative capacity and functional segregation. Rather than merely reversing stress-induced deficits, ketamine may facilitate a reconfiguration of network organization, potentially disrupting maladaptive stable states and allowing transitions toward more adaptive circuit configurations. This interpretation is supported by cross-species evidence from both cellular-electrophysiological and systems-level perspectives^43,44^. The LHb, an epithalamic structure that forms reciprocal connections with midbrain dopamine neurons^45^, is typically activated by reward omission or aversive stimuli and inhibited by reward-predicting cues^46^. A growing body of evidence has established the LHb as a crucial site for ketamine’s antidepressant action^47,48^. In our study, the LHb demonstrated a unique response profile, with consistently elevated local topological properties in the CSDS-ketamine group compared to both control groups. This was in direct contrast to the seven examined brain regions (NAc, Cg1, PrL, IL, vHPC, VTA, and BLA), where CSDS-Ket group values were consistently lower. From a graph-theoretic perspective, this finding indicates that ketamine treatment enhances the centrality and integrative capacity of the LHb uniquely within the multilayer architecture. The elevated within-frequency multilayer degree (WMDegree) and CMDegree suggest that the LHb became a more influential hub, both within specific frequency bands and in mediating cross-frequency interactions. The increased lMPC indicates a more diverse connectivity profile across the multilayer network. We speculate this may reflect a therapeutic recalibration driven by ketamine rather than further exacerbation of its pathological state.

In this study, we also utilized 16S rRNA sequencing to analyze the colonic microbiota of C57 mice, aiming to investigate the impact of CSDS on gut microbial composition and the modulatory effect of ketamine. Alpha and beta diversity analyses revealed that ketamine treatment alleviated CSDS-induced gut microbiota dysbiosis and facilitated the restoration of microbial community homeostasis. At the genus level, we observed significant enrichment of several taxa in the ketamine-treated group, including *g_Colidextribacter*, *g_Anaeroplasma*, *g_Eubacterium_nodatum_group*, *g_NK4A214_group*, *g_Tuzzerella*, and *g_norank_f__Peptococcaceae*. The enrichment of *g_Eubacterium_nodatum_group* is noteworthy, as this phylogenetic cluster includes several established butyrate producers, most notably Eubacterium rectale^49,50^. Butyrate is a key energy source for colonocytes, enhances intestinal barrier function, and has strong anti-inflammatory effects^51^. Similarly, *g_NK4A214_group*, which belongs to the family Lachnospiraceae, is renowned for its capacity to degrade complex dietary fibers and its role as a major producer of short-chain fatty acids (SCFAs), especially butyrate and acetate^52^. The increased abundance of this taxon suggests enhanced butyrate production within the gut microbiome following ketamine treatment. Although functional attributes of *g_norank_f__Peptococcaceae* (within the family Peptococcaceae) require further validation, certain members of this family are implicated in protein fermentation pathways that may contribute to SCFA production. SCFA deficiency has been increasingly associated with neuroinflammatory pathways in depression^53^. Specifically, a reduced abundance of SCFA-producing bacteria can compromise intestinal barrier integrity, leading to increased systemic translocation of microbial components. This process triggers peripheral inflammation through the activation of immune pathways such as TLR4/NF-κB and NLRP3. Furthermore, impaired SCFA signaling diminishes their intrinsic anti-inflammatory effects, which are mediated through mechanisms such as histone deacetylase inhibition and activation of G-protein-coupled receptors. The resulting systemic inflammation may subsequently promote neuroinflammation and disrupt neural homeostasis. The enrichment of SCFA-related bacteria following ketamine administration may reflect a pathway through which gut microbiota contribute to ketamine’s effects, possibly via restoration of SCFA levels.

To evaluate the capacity of the FMBFN analytical framework to characterize multi-frequency neural dynamics and integrate multimodal data, we conducted correlation and exploratory mediation analyses to examine associations between ketamine-altered gut microbiota and brain network topology. The correlation analysis revealed that ketamine-enriched genera, such as *g_Colidextribacter* and *g_norank_f__Peptococcaceae*, were negatively associated with key multilayer topological properties (e.g., MPC, MCC2, MCC3), suggesting that microbial alterations may be related to a more flexible brain network configuration. In addition, exploratory bidirectional mediation models identified statistical patterns linking these microbial shifts with brain network reorganization. Given the small sample size and observational design, these findings should be interpreted as hypothesis-generating rather than as evidence of causal or truly bidirectional effects. Nevertheless, they raise the possibility that ketamine-associated microbial changes and neural network reconfiguration may be coupled within the broader gut–brain axis. Such associations were biologically plausible, because bidirectional gut–brain communication is well established^54–56^, and may operate through multiple convergent pathways, including microbial metabolites, immune and inflammatory signaling, vagal and autonomic pathways, and stress-axis regulation. These mechanisms could influence neuronal excitability, synaptic plasticity, and oscillatory coordination across distributed cortico-limbic circuits, thereby contributing to the multilayer network alterations captured by the FMBFN framework. In this context, our results do not establish mechanistic directionality, but they demonstrate the utility of the FMBFN framework for translating neural activity into system-level descriptors that can be integrated with peripheral biological data to generate testable hypotheses about gut–brain interactions in antidepressant response.

Our study has several limitations. First, due to the intrinsic constraints of CSDS model, only male mice were included. CSDS relies on territorial aggression, which is robust and ethologically relevant in male rodents. In contrast, most strains of female mice display lower levels of aggression, and aggressive male mice tend to mount rather than attack females^57^. Although some studies have attempted to extend social defeat paradigms to females through specific experimental modifications, the behavioral adaptation patterns of defeated males and females are not equivalent^58^. We therefore used male mice in the present study to establish and validate the framework under a well-characterized and reproducible stress model. Nevertheless, this design limits the generalizability of our findings, and future studies will be needed to determine whether the observed results extend to females. Second, cross-frequency connectivity was defined here using phase-based PLI, which provided a consistent basis for multilayer network construction but does not capture all possible forms of CFC. Accordingly, the present findings should be interpreted within this specific methodological framework, and future studies using complementary CFC measures will be needed to test their generality. Third, the gut–brain axis analyses were based on a limited microbiome sample size, and thus the cross-modal correlation and mediation results should be regarded as exploratory and hypothesis-generating. These analyses do not establish causal directionality, and larger, experimentally controlled studies will be necessary to validate the candidate associations reported here.

In summary, our study establishes an FMBFN framework that integrates LFP-based brain-wide fluctuations with gut microbiota profiling. Using this approach, we show that CSDS induces frequency-specific hyperconnectivity and disrupted global integration, while ketamine resets network topology through global simplification and region-specific reorganization, notably enhancing LHb centrality. Moreover, bidirectional interactions between microbial shifts and network reconfiguration highlight a functional gut–brain axis mechanism.Collectively, these findings indicate that the FMBFN framework provides a useful systems-level approach for investigating mechanisms underlying psychiatric disorders and may inform future therapeutic research.

## Methods

### Animals

Given the characteristics of the CSDS model. Male adult C57BL/6 mice (8–12 weeks old) and male CD-1 retired breeder mice (4–6 months old) were obtained from the Experimental Animal Center of Tianjin Medical University, China. Animals were housed in individually ventilated cages (20 air exchanges per hour) under controlled environmental conditions (12 h light/dark cycle, lights on at 07:00, ambient temperature 23 ± 2 °C, relative humidity 50 ± 5%), with ad libitum access to food and water. PVC pipes were provided as environmental enrichment. Prior to the initiation of the CSDS procedure, C57BL/6 mice were group-housed, whereas CD-1 mice were housed individually. Spruce wood shavings were used as bedding. Bedding was changed regularly, and animals were monitored daily for health status and welfare. To minimize potential confounding effects, all animals were housed under identical environmental conditions and subjected to the same experimental procedures throughout the study.

All experimental procedures were approved by the Animal Care and Use Committee of Tianjin Medical University (Approval No. TMUaMEC2021060) and were performed in accordance with institutional guidelines and the ARRIVE 2.0 recommendations. We have complied with all relevant ethical regulations for animal use.

### Chronic social defeat stress procedure

C57BL/6 mice were randomly assigned to either the control (Con) or CSDS group. The CSDS procedure was performed as previously described with minor modifications^59,60^. Briefly, each C57BL/6 mouse in the CSDS group was introduced into the home cage of a novel aggressive male CD-1 retired breeder for 10 min per day for 10 consecutive days, during which it was subjected to physical defeat. After each defeat session, the C57BL/6 mouse was housed in the same cage as the aggressor but separated by a transparent perforated divider for the following 24 h, allowing visual, olfactory, and auditory contact while preventing further physical interaction. A different CD-1 aggressor was used each day to minimize habituation.

Control mice were housed in similar divided cages in pairs with conspecific C57BL/6 mice, with daily cage rotation to match handling and environmental changes, but without exposure to aggressive CD-1 mice.

Throughout the CSDS procedure, all mice were inspected daily for body condition, locomotor activity, coat condition, wound status, and general health. Humane endpoint criteria were established a priori and included severe wounding, substantial weight loss, persistent immobility, inability to access food or water, or signs of severe distress requiring veterinary intervention. Animals meeting these criteria would have been removed from the study and euthanized immediately. No animals met the predefined humane endpoint criteria during the stress procedure.

### Social interaction test

Social avoidance behavior was assessed using the SIT. The apparatus consisted of an open-field arena (40 × 40 × 40 cm) containing a transparent perforated acrylic enclosure positioned along one sidewall. A rectangular zone surrounding the enclosure (14 × 24 cm, extending 8 cm from the enclosure) was defined as the interaction zone.

The test consisted of two consecutive 150-s sessions. In the first session (“No CD1”), the subject mouse was allowed to freely explore the arena in the absence of a social target. In the second session (“With CD1”), a novel male CD-1 mouse was placed inside the enclosure, and the subject mouse was again allowed to explore the arena. Animal movement was video-recorded from above and analyzed using EthoVision XT 8.5 (Noldus, Wageningen, the Netherlands). The primary behavioral variables included time spent in the interaction zone, total distance traveled, and mean velocity. To quantify social avoidance, the SI was calculated as follows:

1 $${\rm{SI}}=\frac{{{\rm{T}}}_{{\rm{With}}}}{{{\rm{T}}}_{{\rm{No}}}}$$

Where ${{\rm{T}}}_{{\rm{With}}}$ represents the time spent in the interaction zone during the “With CD1” session, and ${{\rm{T}}}_{{\rm{No}}}$ represents the time spent in the interaction zone during the “No CD1” session.

Following established criteria^59^, mice exposed to CSDS with SI < 1 were classified as stress-susceptible, indicating the presence of social avoidance behavior, whereas mice with SI ≥ 1 were classified as stress-resilient, approximately 70% show a susceptible phenotype. To characterize abnormal brain activity associated with the social avoidance phenotype induced by CSDS, only stress-susceptible mice (SI < 1) were included in the CSDS group for subsequent analyses. The criterion for stress susceptibility was predefined before data analysis and was based solely on behavioral performance in the SIT, rather than on electrophysiological or microbiome measures.

### Multi-region electrode array fabrication and Stereotaxic surgery

Mice were anesthetized with inhaled isoflurane and secured in a stereotaxic frame (Reward Life Science, Shenzhen, China). Body temperature was maintained during surgery using a heating pad. Ophthalmic ointment was applied to prevent corneal drying. The scalp was disinfected, locally incised, and the skull was exposed under aseptic conditions.

Target brain regions included: BLA (AP 1.6, ML −2.75, DV −3.9); Cg1 (AP −1.8, ML −0.3, DV −1.8); IL (AP −1.8, ML −0.3, DV −2.9); LHb (AP 1.72, ML −0.46, DV −2.62); NAc (AP −1.6, ML −1.1, DV −3.5); PrL (AP −1.8, ML −0.3, DV −2.3); vHPC (AP 3.7, ML −3.0, DV −3.5); VTA (AP 3.3, ML −0.5, DV −4.25). Only regions with correctly positioned electrode tips were included in the final analysis.

A custom multi-region electrode array was constructed using a multi-channel connector and formvar-insulated tungsten wires (0.033 mm diameter; California Fine Wire Co., CA, USA), each with an impedance below 1 MΩ. The electrodes were positioned according to the stereotaxic coordinates of the target regions, with wire lengths adjusted to match the required implantation depth. A common reference and ground were shared across all channels. After electrode placement, the assembly was secured to the skull with dental acrylic, and metal ground screws were attached. All implantations were performed unilaterally on the right hemisphere.

After surgery, mice received routine postoperative monitoring until full recovery from anesthesia and were then returned to their home cages. Animals were monitored at least once daily during the postoperative recovery period for wound healing, body condition, mobility, grooming, and any signs of pain or distress. Cages were kept clean and easily accessible to minimize postoperative burden. Mice were allowed to recover for at least 7 days before behavioral testing and electrophysiological recording. The overall surgical success rate was approximately 95%.

### Neurophysiological recording

All in vivo electrophysiological recordings were conducted using the Cerebus Acquisition System (Blackrock Microsystems Inc., UT, USA), synchronized with behavioral video tracking during the SIT. LFPs were initially bandpass filtered (0.5–250 Hz) and sampled at 2 kHz. Only susceptible mice (SI < 1) for the CSDS group were included in the subsequent analysis. Electrophysiological analysis focused on LFPs while the mice were within the social interaction zone during the “With CD1” phase.

Preprocessing was performed in MATLAB R2023a using the EEGLAB toolbox (version 2023.1). Segments containing amplifier saturation, movement-related artifacts, or excessive noise were automatically identified and excluded using the EEGLAB clean_rawdata routine (“pop_clean_rawdata”). Line noise was attenuated using the EEGLAB filtering routine (“pop_eegfiltnew”) in stopband mode at 49.5–50.5 Hz. This function implements a zero-phase Hamming-windowed sinc finite impulse response (FIR) filter, with filter order and transition bandwidth determined automatically by the built-in heuristic when only cutoff frequencies are specified. Slow baseline drift was reduced using the MATLAB ‘locdetrend‘ function with a moving window of 0.5 s and a step size of 0.25 s.

To minimize edge artifacts associated with narrow-band filtering, frequency decomposition was performed on the continuous preprocessed recordings prior to epoch segmentation. Continuous LFP signals were filtered into five frequency bands using “pop_eegfiltnew()”: band I, 2–7 Hz; band II, 7–12 Hz; band III, 12–30 Hz; band IV, 30–60 Hz; and band V, 60–90 Hz, as defined in a previous study^7^. The resulting band-limited continuous signals were used for subsequent phase extraction and connectivity analysis.

### Drug administration

To evaluate the antidepressant effects and capture significant neuronal responses, a dose of 10 mg/kg ketamine hydrochloride was selected^61,62^. The control group received an i.p. injection of saline. Meanwhile, mice subjected to CSDS were randomly assigned to two subgroups: one was administered a single i.p. injection of ketamine (10 mg/kg), and the other received saline at an equivalent volume. Fecal collection and the SIT were performed 24 h after injection^63^.

### Functional connectivity measurement

Functional connectivity was quantified using the PLI. For each band-limited continuous LFP signal obtained as described above, instantaneous phase was extracted using the MATLAB “hilbert” function. Specifically, the analytic signal was computed for each channel and frequency band, and the phase time series was obtained as the angle of the analytic signal. No phase unwrapping or additional temporal smoothing was applied after phase extraction.

The resulting continuous phase time series were then segmented into consecutive non-overlapping 5-s epochs corresponding to periods in which the animals were located within the social interaction zone during the “With CD1” session. Only artifact-free epochs were included in the connectivity analysis. PLI can be calculated by:

2 $${\rm{PLI}}=\left|\frac{1}{{\rm{N}}}{\sum }_{{\rm{k}}=1}^{{\rm{N}}}{\rm{sign}}\left(\Delta \varphi \left({{\rm{t}}}_{{\rm{k}}}\right)\right)\right|$$

where sign stands for signum, ∆*φ*(*t*_*k*_) denotes the phase difference between the two signals at time${{\rm{t}}}_{{\rm{k}}}$,${\rm{k}}=\left\{1 , 2 , 3{\rm{,...,N}}\right\}$.The phase lag index has a value between 0 and 1, More specifically, PLI = 0 indicates no coupling, while PLI = 1 indicates perfect phase coupling for two signals^64^.

Because PLI is computed from phase differences rather than signal amplitude, spectral power does not directly included the connectivity estimate. This consideration is particularly relevant in the present study, as our previous work demonstrated that CSDS and ketamine alter power spectral density across multiple frequency bands^7^. Nevertheless, differences in oscillatory power may indirectly affect the stability of phase estimation. To minimize this possibility, all groups were processed using identical preprocessing, filtering, phase-extraction, and artifact-rejection procedures.

### Network binarization

In functional connectomics, network thresholding methods are widely employed to eliminate ostensibly false connections, thus enabling the derivation of a binary matrix^65^. Given the substantially stronger within-frequency functional connectivity relative to CFC strength, conventional binarization thresholds (e.g., absolute or proportional criteria) are inadequate for our study. Consequently, we implement a surrogate data approach for network binarization. For each authentic LFP sub-frequency band, 100 surrogate datasets were generated using the amplitude-adjusted Fourier transform method. Functional connectivity matrices (FC) were subsequently computed for both true signals (${{\rm{FC}}}^{{\rm{true}}}$) and surrogate ensembles (${{\rm{FC}}}^{{\rm{rand}}}$). Through element-wise comparison at identical matrix coordinates, we assign ${{\rm{a}}}_{{\rm{ij}}}=1$ when ${{\rm{FC}}}_{{\rm{ij}}}^{{\rm{true}}} > \max \left({{\rm{FC}}}_{{\rm{ij}}}^{{\rm{rand}}}\right)$, and ${{\rm{a}}}_{{\rm{ij}}}=0$ otherwise. This procedure converts the weighted connectivity matrix into a binary adjacency matrix A.

### Frequency-varying multilayer brain functional network (FMBFN)

A network consists of nodes and edges between nodes. In our study, each brain region’s LFP was defined as a node and the WFC or CFC between the nodes as network edges. FMBFN can be defined as ${\rm{G}}=\left({\rm{V}},{\rm{E}}\right)$, expressed as ${{\rm{G}}}=\cup {{{\rm{G}}}}_{{{\rm{f}}}}=\cup \left({{{\rm{V}}}}_{{{\rm{f}}}},{{{\rm{E}}}}_{{{\rm{f}}}}\right)$. Here ${{\rm{f}}}\in {{\rm{F}}}$, where ${{{\rm{F}}}}_{{{\rm{W}}}}\cup {{\rm{F}}}_{{\rm{C}}} = \left\{{{\rm{f}}}_{{\rm{I}}{\rm{I}}},{{\rm{f}}}_{{\rm{II}}{\rm{II}}},{{\rm{f}}}_{{\rm{III}}{\rm{III}}},{{\rm{f}}}_{{\rm{IV}}{\rm{IV}}},{{\rm{f}}}_{{\rm{V}}{\rm{V}}}\right\}\cup \left\{{{\rm{f}}}_{{\rm{I}}{\rm{II}}},{{\rm{f}}}_{{\rm{I}}{\rm{III}}},{{\rm{f}}}_{{\rm{I}}{\rm{IV}}},{{\rm{f}}}_{{\rm{I}}{\rm{V}}},{{\rm{f}}}_{{\rm{II}}{\rm{III}}},{{\rm{f}}}_{{\rm{II}}{\rm{IV}}},{{\rm{f}}}_{{\rm{II}}{\rm{V}}},{{\rm{f}}}_{{\rm{III}}{\rm{IV}}},{{\rm{f}}}_{{\rm{III}}{\rm{V}}},{{\rm{f}}}_{{\rm{IV}}{\rm{V}}}\right\}$ Specifically, the elements of each ${{\rm{V}}}_{{\rm{f}}}=\left\{{{\rm{v}}}_{1},{{\rm{v}}}_{2},{{\rm{v}}}_{3}{\rm{,...,}}{{\rm{v}}}_{{\rm{n}}}\right\}$ represent the nodes of graph ${{\rm{G}}}_{{\rm{f}}}$, where n is the total number of nodes, and elements of each ${{{\rm{E}}}}_{{{\rm{f}}}}\subseteq {{{\rm{V}}}}_{{{\rm{f}}}}\times {{{\rm{V}}}}_{{{\rm{f}}}}$ stand for the edges of the corresponding graph. Actually, FMBFN can be fully characterized by its generalized adjacency matrix ${\rm{A}}$, expressed as ${\rm{A}}=\cup {{\rm{A}}}_{{\rm{f}}}$, where ${{\rm{A}}}_{{\rm{f}}}=\left({{\rm{a}}}_{{\rm{ij}}}^{{\rm{f}}}\right)$, ${\rm{f}}\in {\rm{F}}$. This matrix is derived through a two-step process: first computing the GFC matrix, then applying a binarization threshold, as described in Definitions 1 and 2.

**Definition 1 (Functional connectivity matrix and adjacency matrix)**

Let ${{\rm{r}}}_{{\rm{pq}}}^{{{\rm{f}}}_{{{\rm{\lambda }}}{{\rm{\mu }}}}}$ represent the coupling strength between node ${\rm{p}}$ in the f_λ_-band and node ${\rm{q}}$ in the f_μ_-band. The functional connectivity matrix ${{\rm{FC}}}_{{\rm{f}}}$ is then defined as

3 $${{\rm{FC}}}_{{\rm{f}}}=\left(\begin{array}{cccc}{{\rm{r}}}_{11}^{{\rm{f}}} & {{\rm{r}}}_{12}^{{\rm{f}}} & \cdots & {{\rm{r}}}_{1{\rm{n}}}^{{\rm{f}}}\\ {{\rm{r}}}_{21}^{{\rm{f}}} & {{\rm{r}}}_{22}^{{\rm{f}}} & \cdots & {{\rm{r}}}_{2{\rm{n}}}^{{\rm{f}}}\\ \vdots & \vdots & \ddots & \vdots \\ {{\rm{r}}}_{{\rm{n}}1}^{{\rm{f}}} & {{\rm{r}}}_{{\rm{n}}2}^{{\rm{f}}} & \cdots & {{\rm{r}}}_{{\rm{nn}}}^{{\rm{f}}}\end{array}\right)$$

Subsequently, the adjacency matrix ${{\rm{A}}}_{{\rm{f}}}$ is derived by binarizing ${{\rm{FC}}}_{{\rm{f}}}$

4 $${{\rm{A}}}_{{\rm{f}}}=\left(\begin{array}{cccc}{{\rm{a}}}_{11}^{{\rm{f}}} & {{\rm{a}}}_{12}^{{\rm{f}}} & \cdots & {{\rm{a}}}_{1{\rm{n}}}^{{\rm{f}}}\\ {{\rm{a}}}_{21}^{{\rm{f}}} & {{\rm{a}}}_{22}^{{\rm{f}}} & \cdots & {{\rm{a}}}_{2{\rm{n}}}^{{\rm{f}}}\\ \vdots & \vdots & \ddots & \vdots \\ {{\rm{a}}}_{{\rm{n}}1}^{{\rm{f}}} & {{\rm{a}}}_{{\rm{n}}2}^{{\rm{f}}} & \cdots & {{\rm{a}}}_{{\rm{nn}}}^{{\rm{f}}}\end{array}\right)$$

Here, nodes ${\rm{p}}$ and ${\rm{q}}\in \left\{1 , 2{\rm{,...,n}}\right\}$, n is the total number of nodes, meanwhile, f_λ_ and f_μ_ need to comply with f_λμ_ ∈F, i.e., ${\rm{f}}\in {\rm{F}}$

**Definition 2 (Generalized functional connectivity matrix and generalized adjacency matrix)**

The GFC matrix simultaneously characterizes both WFC and CFC, this matrix can be formally expressed as:

5 $${\rm{GFC}}=\left(\begin{array}{ccccc}{{\rm{FC}}}_{{\rm{I}}{\rm{I}}} & {{\rm{FC}}}_{{\rm{I}}{\rm{II}}} & {{\rm{FC}}}_{{\rm{I}}{\rm{III}}} & {{\rm{FC}}}_{{\rm{I}}{\rm{IV}}} & {{\rm{FC}}}_{{\rm{I}}{\rm{V}}}\\ {{\rm{FC}}}_{{\rm{II}}{\rm{I}}} & {{\rm{FC}}}_{{\rm{II}}{\rm{II}}} & {{\rm{FC}}}_{{\rm{II}}{\rm{III}}} & {{\rm{FC}}}_{{\rm{II}}{\rm{IV}}} & {{\rm{FC}}}_{{\rm{II}}{\rm{V}}}\\ {{\rm{FC}}}_{{\rm{III}}{\rm{I}}} & {{\rm{FC}}}_{{\rm{III}}{\rm{II}}} & {{\rm{FC}}}_{{\rm{III}}{\rm{III}}} & {{\rm{FC}}}_{{\rm{III}}{\rm{IV}}} & {{\rm{FC}}}_{{\rm{III}}{\rm{V}}}\\ {{\rm{FC}}}_{{\rm{IV}}{\rm{I}}} & {{\rm{FC}}}_{{\rm{IV}}{\rm{II}}} & {{\rm{FC}}}_{{\rm{IV}}{\rm{III}}} & {{\rm{FC}}}_{{\rm{IV}}{\rm{IV}}} & {{\rm{FC}}}_{{\rm{IV}}{\rm{V}}}\\ {{\rm{FC}}}_{{\rm{V}}{\rm{I}}} & {{\rm{FC}}}_{{\rm{V}}{\rm{II}}} & {{\rm{FC}}}_{{\rm{V}}{\rm{III}}} & {{\rm{FC}}}_{{\rm{V}}{\rm{IV}}} & {{\rm{FC}}}_{{\rm{V}}{\rm{V}}}\end{array}\right)$$

Then, the super adjacency matrix can be given as

6 $${{\rm{A}}}=\left(\begin{array}{ccccc}{{\rm{A}}}_{{\rm{I}}{\rm{I}}} & {{\rm{A}}}_{{\rm{I}}{\rm{II}}} & {{\rm{A}}}_{{\rm{I}}{\rm{III}}} & {{\rm{A}}}_{{\rm{I}}{\rm{IV}}} & {{\rm{A}}}_{{\rm{I}}{\rm{V}}}\\ {{\rm{A}}}_{{\rm{II}}{\rm{I}}} & {{\rm{A}}}_{{\rm{II}}{\rm{II}}} & {{\rm{A}}}_{{\rm{II}}{\rm{III}}} & {{\rm{A}}}_{{\rm{II}}{\rm{IV}}} & {{\rm{A}}}_{{\rm{II}}{\rm{V}}}\\ {{\rm{A}}}_{{\rm{III}}{\rm{I}}} & {{\rm{A}}}_{{\rm{III}}{\rm{II}}} & {{\rm{A}}}_{{\rm{III}}{\rm{III}}} & {{\rm{A}}}_{{\rm{III}}{\rm{IV}}} & {{\rm{A}}}_{{\rm{III}}{\rm{V}}}\\ {{\rm{A}}}_{{\rm{IV}}{\rm{I}}} & {{\rm{A}}}_{{\rm{IV}}{\rm{II}}} & {{\rm{A}}}_{{\rm{IV}}{\rm{III}}} & {{\rm{A}}}_{{\rm{IV}}{\rm{IV}}} & {{\rm{A}}}_{{\rm{IV}}{\rm{V}}}\\ {{\rm{A}}}_{{\rm{V}}{\rm{I}}} & {{\rm{A}}}_{{\rm{V}}{\rm{II}}} & {{\rm{A}}}_{{\rm{V}}{\rm{III}}} & {{\rm{A}}}_{{\rm{V}}{\rm{IV}}} & {{\rm{A}}}_{{\rm{V}}{\rm{V}}}\end{array}\right)$$

### Topological structure of FMBFN

#### Topological properties of functional segregation

Functional segregation in brain networks refers to the division of specialized cognitive functions across distinct regions^66^. Generalized clustering coefficients assess the functional segregation ability. In a SBFN, the clustering coefficient of a given node ${\rm{p}}$ is defined as the ratio of actual triangles it forms compared to the maximum possible triangles it could establish with all neighboring nodes.

In an FMBFN, the multilayer architecture introduces structural complexity that increases the diversity of triangular configurations. The diversity of triangular configurations significantly impacts the definition of its corresponding clustering coefficients. When all three edges forming a closed triangle reside within the same layer of FMBFN, the generalized clustering coefficient of node ${\rm{p}}$, also known as the local multilayer clustering coefficient ${{\rm{lMCC}}1}_{{\rm{p}}}$, can be defined as

7 $${{\rm{lMCC}}1}_{{\rm{p}}}=\left({\sum}_{{\rm{f}}\in {{\rm{F}}}_{{\rm{W}}}}\left({\sum}_{{\rm{k}}\ne {\rm{p}},{\rm{l}}\ne {\rm{p}}}{{\rm{a}}}_{{\rm{pk}}}^{{\rm{f}}}{{\rm{a}}}_{{\rm{kl}}}^{{\rm{f}}}{{\rm{a}}}_{{\rm{lp}}}^{{\rm{f}}}\right)/\left({\sum}_{{\rm{k}}\ne {\rm{p}},{\rm{l}}\ne {\rm{p}}}{{{\rm{a}}}_{{\rm{pk}}}^{{\rm{f}}}{\rm{a}}}_{{\rm{lp}}}^{{\rm{f}}}\right)\right)/{{\rm{N}}}_{{{\rm{F}}}_{{\rm{W}}}}$$

Where ${{\rm{N}}}_{{{\rm{F}}}_{{\rm{W}}}}$ indicates the total number of bands, with ${{\rm{N}}}_{{{\rm{F}}}_{{\rm{W}}}}=5$.

Additionally, if two edges residing in the same layer and one edge in other layer of FMBFN, while node ${\rm{p}}$ is not in this layer, then the local multilayer cross-two-frequencies clustering coefficient of node ${\rm{p}}$ can be depicted as

8 $${{\rm{lMCC}}2}_{{\rm{p}}}=\left({\sum}_{{{\rm{f}}}_{{{\rm{\lambda }}}}\in {{\rm{F}}}_{{\rm{W}}}}{\sum}_{{{{\rm{f}}}_{{{\rm{\mu }}}}\in {{\rm{F}}}_{{\rm{W}}},{\rm{f}}}_{{{\rm{\mu }}}}\ne {{\rm{f}}}_{{{\rm{\lambda }}}}}{\sum}_{{\rm{k}}\ne {\rm{p}},{\rm{l}}\ne {\rm{p}}}\left({{\rm{a}}}_{{\rm{pk}}}^{{{\rm{f}}}_{{{\rm{\lambda }}}}}{{\rm{a}}}_{{\rm{kl}}}^{{{\rm{f}}}_{{{\rm{\mu }}}}}{{\rm{a}}}_{{\rm{lp}}}^{{{\rm{f}}}_{{{\rm{\lambda }}}}}\right)\right)/ \\ \left(\left({{\rm{N}}}_{{{\rm{F}}}_{{\rm{W}}}}-1\right){\sum}_{{{\rm{f}}}_{{{\rm{\lambda }}}}\in {{\rm{F}}}_{{\rm{W}}}}{\sum}_{{\rm{k}}\ne {\rm{p}},{\rm{l}}\ne {\rm{p}}}{{\rm{a}}}_{{\rm{pk}}}^{{{\rm{f}}}_{{{\rm{\lambda }}}}}{{\rm{a}}}_{{\rm{lp}}}^{{{\rm{f}}}_{{{\rm{\lambda }}}}}\right)$$

Meanwhile, if the edges of a closed triangle extend across three ${{\rm{F}}}_{{\rm{W}}}$-layers of one FMBFN, then the local multilayer cross-three-frequencies clustering coefficient (${{\rm{lMCC}}3}_{{\rm{p}}}$), is defined as

9 $${{\rm{lMCC}}3}_{{\rm{p}}}=\left({\sum}_{{{\rm{f}}}_{{{\rm{\lambda }}}}\in {{\rm{F}}}_{{\rm{W}}}}{\sum}_{{{{\rm{f}}}_{{{\rm{\mu }}}}\in {{\rm{F}}}_{{\rm{W}}},{\rm{f}}}_{{{\rm{\mu }}}}\ne {{\rm{f}}}_{{{\rm{\lambda }}}}}{\sum}_{{{{\rm{f}}}_{{{\rm{\nu }}}}\in {{\rm{F}}}_{{\rm{W}}},{\rm{f}}}_{{{\rm{\nu }}}}\ne {{\rm{f}}}_{{{\rm{\mu }}}}\ne {{\rm{f}}}_{{{\rm{\lambda }}}}}{\sum}_{{\rm{k}}\ne {\rm{p}},{\rm{l}}\ne {\rm{p}}}\left({{\rm{a}}}_{{\rm{pk}}}^{{{\rm{f}}}_{{{\rm{\lambda }}}}}{{\rm{a}}}_{{\rm{kl}}}^{{{\rm{f}}}_{{{\rm{\nu }}}}}{{\rm{a}}}_{{\rm{lp}}}^{{{\rm{f}}}_{{{\rm{\mu }}}}}\right)\right)/\\ \left(\left({{\rm{N}}}_{{{\rm{F}}}_{{\rm{W}}}}-2\right){\sum}_{{{\rm{f}}}_{{{\rm{\lambda }}}}\in {{\rm{F}}}_{{\rm{W}}}}{\sum}_{{{{\rm{f}}}_{{{\rm{\mu }}}}\in {{\rm{F}}}_{{\rm{W}}},{\rm{f}}}_{{{\rm{\mu }}}}\ne {{\rm{f}}}_{{{\rm{\lambda }}}}}{\sum}_{{\rm{k}}\ne {\rm{p}},{\rm{l}}\ne {\rm{p}}}\left({{\rm{a}}}_{{\rm{pk}}}^{{{\rm{f}}}_{{{\rm{\lambda }}}}}{{\rm{a}}}_{{\rm{lp}}}^{{{\rm{f}}}_{{{\rm{\mu }}}}}\right)\right)$$

Additionally, the global multilayer clustering coefficient (${\rm{MCC}}1$), ${\rm{MCC}}2$, and ${\rm{MCC}}3$ can be obtained by averaging the values of their corresponding local measures—${{\rm{lMCC}}1}_{{\rm{p}}}$,${{\rm{lMCC}}2}_{{\rm{p}}}$, and ${{\rm{lMCC}}3}_{{\rm{p}}}$—across all nodes.

#### Topological properties of functional integration

Brain network functional integration refers to the ability of distinct brain regions to communicate and collaborate, forming a unified network that supports complex cognitive processes. The multilayer participation coefficients, characteristic path lengths, global efficiencies and multilayer entropy, characterizing the functional integration capability of FMBFN.

The multilayer participation coefficient measures the extent of nodes involvement in cross-layer information exchange. The local multilayer participation coefficient of node ${\rm{p}}$ can be given as

10 $${{\rm{lMPC}}}_{{\rm{p}}}=\left(1-{\sum}_{{\rm{f}}\in {{\rm{F}}}_{{\rm{W}}}}{\left({{\rm{o}}}_{{\rm{p}}}^{{\rm{f}}}/{{\rm{o}}}_{{\rm{p}}}^{{{\rm{F}}}_{{\rm{W}}}}\right)}^{2}\right)/\left(1-1/{{\rm{N}}}_{{{\rm{F}}}_{{\rm{W}}}}\right)$$

where ${{\rm{o}}}_{{\rm{p}}}^{{\rm{f}}}={\sum }_{{\rm{k}}=1}^{{\rm{n}}}{{\rm{a}}}_{{\rm{pk}}}^{{\rm{f}}}$ and ${{\rm{o}}}_{{\rm{p}}}^{{{\rm{F}}}_{{\rm{W}}}}={\sum }_{{\rm{f}}\in {{\rm{F}}}_{{\rm{W}}}}{{\rm{o}}}_{{\rm{p}}}^{{\rm{f}}}$. The ${\rm{MPC}}$ is computed as the average of its local values (${{\rm{lMPC}}}_{{\rm{p}}}$) across all nodes.

In the FMBFN, which integrates both within-frequency and CFC within a given frequency band ${\rm{f}}$, the concept of characteristic path length is extended to capture information transmission efficiency under different connectivity constraints. When considering only node pairs within the same layer, the multilayer characteristic path length is defined as:

11 $${{\rm{WML}}}=\left({\sum}_{{\rm{f}}\in {{\rm{F}}}_{{\rm{W}}}}\left({\sum}_{{\rm{k}}\ne {\rm{l}}}{{\rm{d}}}_{{\rm{kl}}}^{{\rm{f}}}\right)/\left({\rm{n}}\left({\rm{n}}-1\right)\right)\right)/{{\rm{N}}}_{{{\rm{F}}}_{{\rm{W}}}}$$

where ${{\rm{d}}}_{{\rm{kl}}}^{{\rm{f}}}$ denotes the shortest path length of different nodes ${\rm{k}}$ and ${\rm{l}}$ in ${\rm{f}}$ -layer of FMBFN.

Furthermore, given the existence of f_λμ_-layer of FMBFN, the cross-frequency multilayer characteristic path length can be denoted as

12 $${{\rm{CML}}}=\left({\sum}_{{\rm{f}}\in {{\rm{F}}}_{{\rm{C}}}}\left({\sum}_{{\rm{k}}\ne {\rm{l}}}{{\rm{d}}}_{{\rm{kl}}}^{{{\rm{f}}}_{{{\rm{\lambda }}}{{\rm{\mu }}}}}\right)/{{\rm{n}}}^{2}\right)/\left({{\rm{N}}}_{{{\rm{F}}}_{{\rm{W}}}}\left({{\rm{N}}}_{{{\rm{F}}}_{{\rm{W}}}}-1\right)/2\right)$$

where ${{\rm{d}}}_{{\rm{kl}}}^{{{\rm{f}}}_{{{\rm{\lambda }}}{{\rm{\mu }}}}}$ denotes the shortest path length of node ${\rm{k}}$ in f_λ_-layer and node ${\rm{l}}$ in f_μ_-layer of FMBFN.

The multilayer global efficiency is defined based on the spatial configuration of node pairs as the average of the inverses of the shortest path lengths between all node pairs in the network. When considering only node pairs within the same layer, the within-frequency multilayer global efficiency is defined as:

13 $${{\rm{globalWML}}}=\left({\sum}_{{\rm{f}}\in {{\rm{F}}}_{{\rm{W}}}}\left({\sum}_{{\rm{k}}\ne {\rm{l}}}1/{{\rm{d}}}_{{\rm{kl}}}^{{\rm{f}}}\right)/\left({\rm{n}}\left({\rm{n}}-1\right)\right)\right)/{{\rm{N}}}_{{{\rm{F}}}_{{\rm{W}}}}$$

Meanwhile, the cross-frequency multilayer global efficiency is

14 $${{\rm{globalCML}}}=\left({\sum}_{{\rm{f}}\in {{\rm{F}}}_{{\rm{C}}}}\left({\sum}_{{\rm{k}}\ne {\rm{l}}}{1/{\rm{d}}}_{{\rm{kl}}}^{{{\rm{f}}}_{{{\rm{\lambda }}}{{\rm{\mu }}}}}\right)/{{\rm{n}}}^{2}\right)/\left({{\rm{N}}}_{{{\rm{F}}}_{{\rm{W}}}}\left({{\rm{N}}}_{{{\rm{F}}}_{{\rm{W}}}}-1\right)/2\right)$$

The multilayer entropy quantifies the heterogeneity of connection patterns across all nodes in different layers of the network. It is defined as follows:

15 $${{\rm{ME}}}=-{\sum}_{{\rm{f}}\in {{\rm{F}}}_{{\rm{w}}}}{\sum}_{{\rm{p}}\in {{\rm{V}}}_{{\rm{f}}}}\left(\left({{\rm{o}}}_{{\rm{p}}}^{{\rm{f}}}/{{\rm{o}}}_{{\rm{p}}}^{{{\rm{F}}}_{{\rm{W}}}}\right){\mathrm{ln}}\left({{\rm{o}}}_{{\rm{p}}}^{{\rm{f}}}/{{\rm{o}}}_{{\rm{p}}}^{{{\rm{F}}}_{{\rm{W}}}}\right)\right)$$

#### Degree

The degree of a node is a fundamental graph-theoretic measure that reflects its importance and connectivity within a network. In functional brain networks, nodes with high degree are considered hubs and play a central role in facilitating information exchange^67^.

In the FMBFN, this concept naturally extends into two distinct dimensions. When considering connectivity within the same layer, the within-frequency multilayer degree of node *P* can be given as

16 $${{\rm{WMDegree}}}_{{\rm{p}}}={{\rm{o}}}_{{\rm{p}}}^{{{\rm{F}}}_{{\rm{W}}}}/{{\rm{N}}}_{{{\rm{F}}}_{{\rm{W}}}}$$

Furthermore, accounting for connections across different layers of the FMBFN, the cross-frequency multilayer degree of node *P* can be given as

17 $${{\rm{CMDegree}}}_{{\rm{p}}}={{\rm{o}}}_{{\rm{p}}}^{{{\rm{F}}}_{{\rm{C}}}}/\left({{\rm{N}}}_{{{\rm{F}}}_{{\rm{W}}}}\left({{\rm{N}}}_{{{\rm{F}}}_{{\rm{W}}}}-1\right)/2\right)$$

where $${{\rm{o}}}_{{\rm{p}}}^{{{\rm{F}}}_{{\rm{C}}}}={\sum }_{{\rm{f}}\in {{\rm{F}}}_{{\rm{C}}}}{{\rm{o}}}_{{\rm{p}}}^{{\rm{f}}}$$ and $${{\rm{o}}}_{{\rm{p}}}^{{\rm{f}}}={\sum }_{{\rm{k}}=1}^{{\rm{n}}}{{\rm{a}}}_{{\rm{pk}}}^{{\rm{f}}}$$.

### Fecal sample collection, 16S rRNA sequencing, and analyses

Fecal samples were collected 24 h after ketamine injection and immediately before LFPs recordings to synchronize metabolomic profiling with neural activity assessments. All samples were obtained aseptically and cryopreserved at −80 °C. DNA extraction, polymerase chain reaction (PCR) amplification, and Illumina MiSeq sequencing were were conducted as described previously^68^. Raw fastq files were merged with FLASH, and amplicon sequence variants (ASVs) were generated using DADA2 with default parameters. Sequences were rarefied to 20,000 reads per sample, achieving an average Good’s coverage of 99.09%. Taxonomic assignment of ASVs was conducted using a Naïve Bayes classifier. Alpha diversity, evaluated through the Shannon indices, was computed in Mothur. Beta diversity was analyzed via principal coordinate analysis (PCoA) based on Bray–Curtis dissimilarity, with statistical significance assessed using permutational multivariate analysis of variance. The horizontal and vertical axes in the PCoA plots correspond to PCo1 and PCo2 scores, respectively. Differentially enriched bacterial taxa between groups were identified with LEfSe analysis (LDA score > 2, *P* < 0.05).

### Correlation analysis and mediation analysis

Spearman’s correlation analysis was used to evaluate associations between behavioral measures, topological properties, and microbial features. The Benjamin–Hochberg method was applied to control the false discovery rate (FDR) for multiple comparisons. To explore potential mediation relationships among ketamine administration, topological properties, and microbial features, we conducted mediation analyses using the SPSS PROCESS Macro (version 4.2, Model 4).

### Support vector machines

To quantitatively evaluate the discriminative power of the extracted neural features, we performed both binary and multiclass classification using a Support Vector Machine (SVM) implemented with the LIBSVM toolbox (version 3.32) in MATLAB R2023a. The multiclass classification was conducted using the built-in one-vs-one strategy. Classification features consisted of 24 dimensions, including 9 global topological properties (MPC, WML, CML, global WML, globa lCML, ME, MCC1, MCC2, and MCC3) and 15 connectivity features obtained by averaging entire functional connectivity matrices. Specifically, for each of the 15 functional connectivity matrices (5 within-frequency and 10 cross-frequency), all elements in the matrix were averaged to yield a single scalar representing the overall connectivity strength for that particular frequency band or cross-frequency interaction. Each sample represented a single 5-second epoch, with all epochs from all mice across groups pooled for analysis. To ensure robust results, we repeated the classification procedure 100 times with random splits of the dataset into training (70%) and testing (30%) sets for each iteration. During training, we employed tenfold cross-validation for parameter optimization to enhance generalization and reduce over fitting. Additionally, to establish a statistical baseline representing chance-level performance, we performed 100 iterations of shuffle control by randomly permuting the class labels relative to the neural features, with the resulting classification accuracies forming a null distribution for statistical comparison against real data performance^69^. Prior to model training, all features were z-score standardized. Model performance was evaluated using accuracy, ROC curves, and area under the ROC curve (AUC). Classification accuracy was calculated from confusion matrices, while ROC curves were generated by plotting the true positive rate against the false positive rate across different classification thresholds. AUC values were derived from these ROC curves as a comprehensive metric for classifier performance.

### Statistics and reproducibility

Analyses were organized by experimental module, with one primary endpoint defined for each module to guide sample-size interpretation Supplementary Table 2. Required sample sizes for these endpoints were estimated with reference to previously published studies^20,70^ Sensitivity analyses were performed for the pre-specified primary endpoint of each module using *α* = 0.05 and target power = 0.80, and the resulting minimum detectable effect sizes are reported in Supplementary Table 2. Final sample sizes differed across modules because the modules addressed different aims and were subject to different predefined inclusion criteria.

Analyses were performed in a manner blinded to treatment assignments in all behavioral experiments. Statistical analyses were conducted using GraphPad Prism or MATLAB software. For statistical comparisons between two groups, data distribution was first assessed for normality using the Shapiro-Wilk test and for homogeneity of variances using Bartlett’s test. If both normality and homoscedasticity assumptions were satisfied, an unpaired Student’s t-test was employed. For data meeting the normality assumption but violating homogeneity of variances, Welch’s t-test was applied. When the normality assumption was not met, the non-parametric Mann-Whitney U test was used. For comparisons among three groups, the same preliminary assessments of normality and homoscedasticity were conducted. When both assumptions were met, ordinary one-way ANOVA followed by Tukey’s post hoc test for multiple comparisons was performed. For data with normal distribution but unequal variances, Brown-Forsythe and Welch ANOVA tests were conducted with Dunnett’s T3 post hoc analysis. When the normality assumption was violated, the non-parametric Kruskal-Wallis test was applied, followed by Dunn’s post hoc test for multiple comparisons. Data are presented as the mean ± SEM. Significance levels were established at *P < 0.05, ** *P* < 0.01, *** *P* < 0.001, ns. denoting not significant.

For all group-level statistical comparisons, with the exception of the SVM-based classification analyses, the inferential unit was the mouse. Specifically, network measures were first computed for each 5-s epoch and then averaged across epochs within each mouse before statistical testing. This approach was used to avoid treating repeated epochs from the same mouse as independent biological replicates and to preserve the nested structure of the data.

(F–M) Bidirectional mediation analyses among ketamine treatment, gut microbiota, and global topological properties. Path coefficients (*β*) and their significance are shown on the edges, and mediation *P* values with 95% CIs are shown in the center. Red and blue arrows indicate significant mediation effects. All *P* values were corrected for multiple testing using the Benjamini–Hochberg FDR procedure. Detailed statistical methods and full statistical results are provided in Supplementary Table 3. Con-Sal, *n* = 5; CSDS-Sal, *n* = 5; CSDS-Ket, *n* = 5. (F–I) Microbiota-mediated effects: ketamine influences network properties through gut microbiota. (J–M) Network-mediated effects: ketamine influences gut microbiota through brain network reorganization.

### Reporting summary

Further information on research design is available in the Nature Portfolio Reporting Summary linked to this article.

## Supplementary information

Transparent Peer Review file

Supplemental Information

Description of Additional Supplementary Files

Supplementary Data

Reporting Summary

## Data Availability

The numerical source data underlying all graphs and charts presented in the main figures have been deposited in Figshare and are publicly available at the following link: https://doi.org/10.6084/m9.figshare.30472571^72^. The raw and processed 16S rRNA sequencing data generated in this study have been included in the Figshare repository https://doi.org/10.6084/m9.figshare.32672760^73^). Raw electrophysiological recordings, processed datasets, and all other data supporting the findings of this study are available from the corresponding author upon reasonable request. Received: ((will be filled in by the editorial staff)) Revised: ((will be filled in by the editorial staff)) Published online: ((will be filled in by the editorial staff)).

## Code availability

Data was analyzed using EEGLAB (version 2023.1), Chronux toolbox (version 2.12), Brain Connectivity Toolbox (version 2019_03_03), and MATLAB (R2023a). Custom MATLAB scripts used for frequency-varying multilayer brain functional network analysis are publicly available on Figshare at https://doi.org/10.6084/m9.figshare.30475247^71^.
