# Supplementary material for: Multilayer brain network analysis in mice reveals ketamine-induced reorganization of brain- wide fluctuations and gut-brain axis
Source: Commun Biol. 2026 Jul 3;9:1399. doi: 10.1038/s42003-026-10606-0 (PMC13696714; doi:10.1038/s42003-026-10606-0)
Supplement: Supplementary file 1 [file 42003_2026_10606_MOESM1_ESM.pdf]

nature portfolio

Peer Review File

Multilayer Brain Network Analysis in Mice Reveals Ketamine-Induced Reorganization of Brain-wide Fluctuations and Gut-Brain Axis

Corresponding Author: Dr Tiaotiao Liu

This file contains all reviewer reports in order by version, followed by all author rebuttals in order by version.

Version 0:

Reviewer comments:

Reviewer #1

(Remarks to the Author)
I co-reviewed this manuscript with one of the reviewers who provided the listed reports. This is part of the Communications Biology initiative to facilitate training in peer review and to provide appropriate recognition for Early Career Researchers who co-review manuscripts.

This paper presents an analytical, systems-level framework for “characterising brain-wide neural fluctuations and topological dimensions” in vivo in mice. The studies are well-planned, with each research question relating coherently to the next: establishing the novel frequency-varying multilayer brain functional network (FMBFN) framework; applying the FMBFN framework in a chronic social defeat stress (CSDS) model; applying the FMBFN framework to investigate mechanisms behind a reversal of CSDS effects with ketamine; relating recorded neural oscillations to gut-microbial features; using classification models to test the discriminative power and biological relevance of the neural and microbial features in question.

The necessity for a high-dimensional approach is explicitly clarified, and the FMBFN framework is described in sufficient detail for the reader to understand and implement the systems-level framework. Furthermore, this paper demonstrates the utility of such an approach, successfully applying it in a translational psychiatry context and illuminating the complex neural dynamics behind different brain states. The FMBFN approach integrates spectral and organisational dimensions, allowing for a richer interpretation of the neural recordings in comparison to traditional single-dimension approaches.

The need for the FMBFN framework is particularly clear for the ketamine study, with emphasis placed on the issue that “the mechanisms by which ketamine induces multidimensional neural alterations … are not well understood.” In this regard, with ketamine exerting widespread, multidimensional effects, there is a need for an approach that goes beyond single-frequency analyses which “assume static operations within isolated frequency bands, neglecting the brain's dynamic cross-frequency coordination across multiple spectral domains.”

Collectively, the experiments in this study appear well-planned and logically motivated. The CSDS model of social avoidance is well-motivated and outlined clearly as a valid model of depressive-like behaviour (social avoidance). It is also described in sufficient detail for replication, along with the social interaction test, although dimensions should be given for the CSDS arena. Furthermore, in the “Functional Connectivity Measurement” section of the paper, the mathematical equations underpinning the FMBFN framework are described in sufficient detail and, although rather dense, appear theoretically sound. Overall, there is generally sufficient detail for replication of the various behavioural tasks and analyses.

However, a significant shortcoming is that in addressing a novel method, effects of CSDS, ketamine, and gut microbiome, the paper is trying to do too much, and this overly broad scope detracts from the paper’s impact. Perhaps the paper should be split into two or even three separate papers (1); in its current form it is too dense and attempts to deal simultaneously with too many research questions. This is particularly true for the gut-microbiome content, which does not appear particularly well-motivated - there is only one sentence linking the gut to antidepressant mechanisms - “Emerging evidence indicates

that ketamine alters the composition of gut microbial (17-19), prompting investigations into the role of the gut-brain axis in its antidepressant effects." Greater justification should be given for including gut-microbial features, with proper reference to previous literature (2). Relatedly, it is generally unclear why a "comprehensive tool that bridges brain-wide functional architecture and gut microbiota" is needed. The greatest strength of this paper is the development of the FMBFN analytic framework - greater focus on this in terms of motivation, similar precedents in the literature, and interpretations would be helpful. The excess of variables in this paper is also reflected in the inclusion of too many abbreviations, which are very difficult to follow, hampering the reader's ability to meaningfully interpret results (1). Finally, the inclusion of too many research questions and variables is reflected in the abstract, which is too dense and difficult to follow and appears poorly connected to the content of the manuscript (1).

Regarding figures, Figure 1B-1H provides examples of good visualisation, successfully conveying the FMBFN framework in sufficient detail, outlining relatively complex processes in a coherent manner. However, the figures generally contain too much information and are far too small (e.g., Figure 2U and Figure 3Q-R.) for the reader to meaningfully interpret them (3). This is especially true for the figure legends; much of the information contained within the figure legends should be separated, as it is difficult to locate the relevant information. This is particularly problematic as sample sizes are found only in the figure legends, making it difficult to find this important information (4).

Regarding sample size, a brief mention is made of how sample sizes were chosen based on previous studies of a similar nature. However, there are many tests performed in this paper, so it is unclear which outcome measures or tests would be used as a reference for other papers (4). Also, there are no explicit citations for these previous studies, nor are there any power analyses carried out with previous effect sizes (5).
It is unclear why sample sizes vary so much between studies - this should be justified (4).

Statistics are also reported only in the figure legends, which makes it difficult to locate and interpret the results of each test (6). No effect sizes are reported, which lessens the interpretability and reproducibility of the results (7). Also, exact p-values are not reported, but rather given as approximations (e.g., "$p < 0.05$" or "$p < 0.05$"), which obscures the true significance of each test statistic (8). The use of False Discovery Rate (FDR) corrections is welcome and necessary for such an exploratory study, although it is unclear why this was not used for the mediation analyses (9).

There are multiple issues in the reporting of animal information in all studies. First, only males were included, with no explanation given as to why females were excluded (10). This limits the generalisability of the findings in this study to only males, which is an important caveat. Furthermore, as per ARRIVE guidelines (11) for reporting animal experiments (https://arriveguidelines.org/arrive-guidelines), as referenced in https://www.nature.com/commsbio/submit/submission-guidelines, recommendation 15, housing and husbandry details should be included - for example, were the animals in open-top cages or individually ventilated cages? Was there enrichment in the cages for animal welfare? Also, given the invasive nature of the surgery and local-field potential recording methodology, it is surprising that no information is given regarding the post-surgery care. Furthermore, no information is given as to animal attrition post-surgery. (12)

Finally, apart from in the discussion section, there are repeated instances of inappropriate or incorrect citing of previous research (13). This includes citations such as 1, 3, 5, 9, 10, 21, and 25, which link to relevant papers, yet do not directly support the accompanying claims. This may be as a result of indirect or secondary citings, as seems to be the case for citation 25. Moreover, citations such as 6, 16-19, and 48-50, for example, are certainly relevant, but more robust, exemplary, landmark papers should be cited to support such claims, if possible.

Specific comments and recommendations:
1) Consider narrowing the focus of the paper. There are too many different research questions, variables, abbreviations, and analyses for the reader to follow or to tell a coherent story. The abstract is only weakly connected to the content of the paper.
2) Gut-microbiome content is not well motivated. A greater depth of scholarship and reference to robust evidence is needed in the introduction to justify the inclusion of gut-brain axis analyses.
3) Figures contain too much information, and are cramped together, making it difficult to understand the figures and the accompanying legends. Spread them out, and put some information in the legend in the text body instead, or elsewhere (e.g., a table of statistical results). Text must be larger to be legible.
4) Sample sizes are inconsistent and difficult to locate. Consider placing them all in one place (e.g., a table), with justifications for each final sample size. At the very least, the sample size for each part of the experiment should be described at the beginning of each results section.
5) Power analyses should be reported for each test.
6) Statistics should be reported for each test in a more easily findable manner.
7) No effect sizes are reported for tests done. Report along with the other statistics.
8) Exact p-values are not reported, but rather given as approximations. Report exact p-values
9) No FDR corrections applied to mediation analyses. Apply them as required.
10) Only male rats are included. This should be justified or the experiments should be repeated with females.
11) Refer to the ARRIVE guidelines to ensure proper reporting of animal information (https://arriveguidelines.org/arrive-guidelines).
12) Include information about animal care both during and after surgery.
13) Incorrect or irrelevant citations, weakening the paper's theoretical underpinnings. Revisit the following citations and reconsider their relevance: 1, 3, 5, 6, 9, 10, 16-19, 21, 25, 48-50.
14) Title should specify the fact that mice were used: "Multilayer Brain Network Analysis in mice Reveals Ketamine-Induced Reorganization of Brain- wide Fluctuations and Gut-Brain Axis"

Reviewer #2

(Remarks to the Author)
I co-reviewed this manuscript with one of the reviewers who provided the listed reports. This is part of the Communications Biology initiative to facilitate training in peer review and to provide appropriate recognition for Early Career Researchers who co-review manuscripts.A

Reviewer #3

(Remarks to the Author)
This manuscript presents an innovative and technically ambitious attempt to integrate behaviour, multi-region LFP-derived network physiology, and 16S rRNA gut microbiome profiling to probe ketamine's antidepressant-related effects in CSDS mice. The frequency-varying multilayer brain functional network (FMBFN) framework is clearly motivated, and the main results are interesting and consistent with existing literature. Further, the correlation between neural and gut-microbiome data is a highly relevant emerging topic for mental health treatment research. However, there are some methodological and interpretative considerations to be addressed, especially regarding the statistical approach.
Major comments
- In the materials, it is described how only the CSDS-sensitive animals were selected for the analysis. It would be good to report the number of animals that were sensitive to the behavioural test. What characteristics made certain animals sensitive, and some others not? What was the criterion and threshold approach used to select those?
- In Figure 2 (I–Q) and related analyses, the authors report individual 5-second epochs as the basic analytic unit for the FMBFN analysis. The authors should clarify why the data were not averaged across epochs for each mouse. Further, if any inferential statistics are performed at the epoch level (or on features derived per-epoch) without accounting for repeated measures within mouse, then the analysis is pseudo-replicated and should be re-analysed using an approach that respects nesting (e.g., linear mixed-effects models with mouse as a random effect, or averaging to one value per mouse before group statistics). Relatedly, the SVM validation explicitly treats each 5-second epoch as one sample, pooling epochs from all mice across groups with repeated random train/test splits and a shuffle-label baseline. This design risks inflated accuracy/AUC if epochs from the same mouse appear in both the training and test sets (non-independence / data leakage), so the classification should be repeated using animal-level splitting. Please implement statistical and machine-learning analyses that properly account for the nested structure (epochs within mice) and ensure that the conclusions are robust to these choices, and the limitations (mainly the same size) are clearly stated. Also, for clarity and reproducibility, please standardise and explicitly label for every analysis/figure: number of mice, number of epochs/trials contributing per mouse, whether values are averaged per mouse before statistics, and whether the reported P values and ML validation are computed at the mouse level or epoch level (including how cross-validation splits were performed).
- The framework quantifies within- and cross-frequency coupling using the phase-lag index (PLI) after bandpass filtering and Hilbert-phase extraction, including cross-frequency PLI computed between the phase time series of different bands/regions. Because ketamine (and stress) can robustly alter spectral power, a clearer explanation is needed of whether, and how, power differences are handled, especially for cross-frequency comparisons where band-specific power may differ between groups. This needs to be elaborated more in the methods, alongside a detailed report of filter parameters, Hilbert implementation details, edge handling, exact preprocessing steps, software packages/versions for each step, and QC metrics of the recordings.
- The manuscript defines cross-frequency connectivity (CFC) as PLI between phase time series from different frequency bands. However, this is not the common definition of CFC, which usually reflects the more well-studied metrics of phase–amplitude coupling (PAC) or amplitude–amplitude coupling. The authors should justify why cross-band PLI is an appropriate CFC metric here or add analyses with a conventional CFC method and show that key conclusions are robust using the above-mentioned statistical approaches. In addition, the Discussion requires a more neurophysiologically grounded interpretation of phase–phase CFC. What biological mechanism is proposed to underlie a change in phase-lag index between frequency bands between different regions? How is this related to more classical forms of functional connectivity?
- The paper indicates Benjamini–Hochberg FDR control for multiple comparisons in correlation contexts and that figure P values are FDR-corrected. Given the large number of connectivity matrices, many global and local topology metrics, and multiple regions, it would be important to define the exact "families" of tests entering each FDR correction (ideally in a supplementary table), especially because network metrics are not independent.
- The binarisation process to extrapolate the network metrics is described in the methods. However, a sensitivity analysis across thresholds should be reported, including network edge densities by layer and group, and show that topological effects persist after network edge density-matching.
- The manuscript reports "bidirectional mediation" between ketamine-altered microbiome genera and network metrics and suggests potential causal insights, with microbiome group sizes of n=5 per group. With such a small n and an observational design, a mediation analysis is hardly justified. The Discussion should more explicitly treat mediation as exploratory and hypothesis-generating (not causal proof) and outline concrete causal follow-up experiments that would test directionality. It would also be important to give a physiological interpretation of such a correlation. Why was the correlation expected, and what are the mechanisms that underlie it?
- Please add a clear limitation section. For instance, the generalisability of the finding is limited given that only male mice were used and there are known sex differences in stress susceptibility, neurophysiology, and ketamine response. Further, the limited sample size makes the correlations exploratory as they don't seem justified by power calculations, especially the one between neural and gut microbiome data.
Minor comments
- It would have been interesting to show whether the effect of ketamine was specific to the CSDS mice as compared to WT, given that additional ketamine-induced neurophysiological effects were observed beyond reversing the CSDS phenotype. If any data on ketamine effects in WT or CSDS mice are available would be good to report them as well.

- The sentence “To transform complex microbial community data into well-defined features for integration with FMBFN analytical framework.” is out of place and reads like an editing fragment (around lines 469–471).
- Typo/heading issue: “Topological properties of functional segregation” appears to introduce integration metrics (participation coefficient, path length, efficiency, entropy), so the heading should likely be “integration,” not “segregation” (around line ~885).
- Additional copyedits spotted in Methods: “Illumina MiSeq sequencing were were conducted…” (duplicate “were”).

Version 1:

Reviewer comments:

Reviewer #1

(Remarks to the Author)
This resubmission adequately addressed all of our comments - although we feel the paper remains rather long and complex - potentially difficult for readers to digest.

Please note that this review was completed using the ECR scheme.

Reviewer #2

(Remarks to the Author)
I co-reviewed this manuscript with one of the reviewers who provided the listed reports. This is part of the Communications Biology initiative to facilitate training in peer review and to provide appropriate recognition for Early Career Researchers who co-review manuscripts.

Reviewer #3

(Remarks to the Author)
Thank you for your thorough and careful revision of the manuscript "Multilayer Brain Network Analysis in Mice Reveals Ketamine-Induced Reorganization of Brain-wide Fluctuations and Gut-Brain Axis." The authors have engaged constructively and in good faith with all major and minor comments raised in the review. I am pleased to recommend acceptance for publication.

Open Access This Peer Review File is licensed under a Creative Commons Attribution 4.0 International License, which permits use, sharing, adaptation, distribution and reproduction in any medium or format, as long as you give appropriate credit to the original author(s) and the source, provide a link to the Creative Commons license, and indicate if changes were made.
In cases where reviewers are anonymous, credit should be given to 'Anonymous Referee' and the source.
The images or other third party material in this Peer Review File are included in the article’s Creative Commons license,

unless indicated otherwise in a credit line to the material. If material is not included in the article's Creative Commons license and your intended use is not permitted by statutory regulation or exceeds the permitted use, you will need to obtain permission directly from the copyright holder.
To view a copy of this license, visit https://creativecommons.org/licenses/by/4.0/

Revision explanation on manuscript COMMSBIO-25-10210

Title: Multilayer Brain Network Analysis in Mice Reveals Ketamine-Induced Reorganization of Brain- wide Fluctuations and Gut-Brain Axis

Dear Editor and Reviewers,

Thank you for your constructive and insightful feedback on our manuscript. We are encouraged that the methodological innovation of the FMBFN framework and its potential for characterizing multidimensional brain dynamics in vivo were recognized.

In this revised version, we have implemented substantial systematic improvements to strengthen thematic focus, methodological rigor, and reporting transparency. Specifically, we have:

1. Restructured the manuscript to clearly position FMBFN framework as the central methodological contribution;
2. Refined the statistical pipeline to eliminate potential pseudo-replication and prevent data leakage;
3. Enhanced reporting of experimental procedures in full accordance with ARRIVE 2.0 guidelines.

A detailed, point-by-point response to all comments is provided below.

Responses to the comments of the reviewers

Thank you for your review. We value your feedback and have implemented the suggested changes in the manuscript. Our responses to your comments are provided below. Please note that the page numbers refer to the revised manuscript with the changes highlighted.

Reviewer #1 (Comments to the Author):

Comment: I co-reviewed this manuscript with one of the reviewers who provided the listed reports. This is part of the Communications Biology initiative to facilitate training in peer review and to provide appropriate recognition for Early Career Researchers who co-review manuscripts.

This paper presents an analytical, systems-level framework for "characterising brain-wide neural fluctuations and topological dimensions" in vivo in mice. The studies are well-planned, with each research question relating coherently to the next: establishing the novel frequency-varying multilayer brain functional network (FMBFN) framework; applying the FMBFN framework in a chronic social defeat stress (CSDS) model; applying the FMBFN framework to investigate mechanisms

behind a reversal of CSDS effects with ketamine; relating recorded neural oscillations to gut-microbial features; using classification models to test the discriminative power and biological relevance of the neural and microbial features in question.

The necessity for a high-dimensional approach is explicitly clarified, and the FMBFN framework is described in sufficient detail for the reader to understand and implement the systems-level framework. Furthermore, this paper demonstrates the utility of such an approach, successfully applying it in a translational psychiatry context and illuminating the complex neural dynamics behind different brain states. The FMBFN approach integrates spectral and organisational dimensions, allowing for a richer interpretation of the neural recordings in comparison to traditional single-dimension approaches.

The need for the FMBFN framework is particularly clear for the ketamine study, with emphasis placed on the issue that "the mechanisms by which ketamine induces multidimensional neural alterations … are not well understood." In this regard, with ketamine exerting widespread, multidimensional effects, there is a need for an approach that goes beyond single-frequency analyses which "assume static operations within isolated frequency bands, neglecting the brain's dynamic cross-frequency coordination across multiple spectral domains. "

Collectively, the experiments in this study appear well-planned and logically motivated. The CSDS model of social avoidance is well-motivated and outlined clearly as a valid model of depressive-like behaviour (social avoidance). It is also described in sufficient detail for replication, along with the social interaction test, although dimensions should be given for the CSDS arena. Furthermore, in the "Functional Connectivity Measurement" section of the paper, the mathematical equations underpinning the FMBFN framework are described in sufficient detail and, although rather dense, appear theoretically sound. Overall, there is generally sufficient detail for replication of the various behavioural tasks and analyses.

However, a significant shortcoming is that in addressing a novel method, effects of CSDS, ketamine, and gut microbiome, the paper is trying to do too much, and this overly broad scope detracts from the paper's impact. Perhaps the paper should be split into two or even three separate papers (1); in its current form it is too dense and attempts to deal simultaneously with too many research questions. This is particularly true for the gut-microbiome content, which does not appear particularly well-motivated - there is only one sentence linking the gut to antidepressant mechanisms - "Emerging evidence indicates that ketamine alters the composition of gut microbial (17-19), prompting investigations into the role of the gut-brain axis in its antidepressant effects." Greater justification should be given for including

gut-microbial features, with proper reference to previous literature (2). Relatedly, it is generally unclear why a “comprehensive tool that bridges brain-wide functional architecture and gut microbiota” is needed. The greatest strength of this paper is the development of the FMBFN analytic framework - greater focus on this in terms of motivation, similar precedents in the literature, and interpretations would be helpful. The excess of variables in this paper is also reflected in the inclusion of too many abbreviations, which are very difficult to follow, hampering the reader’s ability to meaningfully interpret results (1). Finally, the inclusion of too many research questions and variables is reflected in the abstract, which is too dense and difficult to follow and appears poorly connected to the content of the manuscript (1).

Specific comments and recommendations:

1) Consider narrowing the focus of the paper. There are too many different research questions, variables, abbreviations, and analyses for the reader to follow or to tell a coherent story. The abstract is only weakly connected to the content of the paper.

2) Gut-microbiome content is not well motivated. A greater depth of scholarship and reference to robust evidence is needed in the introduction to justify the inclusion of gut-brain axis analyses.

Response:

We sincerely appreciate this thoughtful and constructive feedback. We agree that the original scope was overly broad, which may have diluted the impact of our central findings.

Manuscript Restructuring: We have fundamentally reorganized the manuscript to prioritize the FMBFN framework as the core contribution. The CSDS and ketamine models are now presented primarily as biological validation components.

Reframing the Gut-Brain Axis: We have expanded the Introduction **(Page 4, Lines 86-94)** to better contextualize the microbiota-gut-brain axis within antidepressant literature. Crucially, the gut-related analyses are now explicitly framed as an exploratory cross-modal extension.

Readability: Non-essential analyses have been moved to the **Supplement Information (Figure S8)**, and a comprehensive Summary Table of Abbreviations (**Table S1, Supplementary Information**) has been added to improve clarity. The Abstract has also been rewritten to reflect this refined narrative.

Comment: Regarding figures, Figure 1B-1H provides examples of good visualisation, successfully conveying the FMBFN framework in sufficient detail, outlining relatively complex processes in a coherent manner. However, the figures generally contain too much information and are far too small (e.g., Figure 2U and Figure 3Q-R.) for the reader to meaningfully interpret them (3). This is especially true for the figure

legends; much of the information contained within the figure legends should be separated, as it is difficult to locate the relevant information. This is particularly problematic as sample sizes are found only in the figure legends, making it difficult to find this important information (4).

Specific comments and recommendations:

3) Figures contain too much information, and are cramped together, making it difficult to understand the figures and the accompanying legends. Spread them out, and put some information in the legend in the text body instead, or elsewhere (e.g., a table of statistical results). Text must be larger to be legible.

Response:

Thank you for pointing out these presentation issues. In the revised manuscript, we substantially reorganized the main figures and accompanying legends to improve readability and reduce visual overload.

Specifically, the original **Figure 2** was divided into revised **Figures 2 and 3**: **Figure 2** now focuses on the CSDS phenotype, functional connectivity strength, and global topological properties, whereas **Figure 3** presents the local topological alterations and the binary classification results separately. We also replotted the local topology results as a heatmap with representative bar plots to improve clarity. Similarly, the original **Figure 3** was reorganized into revised **Figures 4 and 5**: **Figure 4** now presents the ketamine-related behavioral results, functional connectivity changes, and global topological reorganization, while **Figure 5** separately shows the region-specific local topological effects and the three-class classification results. In addition, the detailed genus-level biomarker plots were moved to the **Supplementary Information (Figure S7)**, and the main-text **Figure 6** was streamlined to focus on the experimental design, alpha/beta diversity, and the cross-modal correlation/mediation analyses.

Across **Figures 1–6**, we also enlarged fonts, simplified panel layouts, reduced descriptive text in the legends, and moved detailed statistical parameters to the **Supplementary Tables** to further enhance clarity. Detailed statistical parameters (e.g., test statistics, exact P-values) have been moved to **Supplementary Data** to reduce clutter in the figure legends.

Comment: Regarding sample size, a brief mention is made of how sample sizes were chosen based on previous studies of a similar nature. However, there are many tests performed in this paper, so it is unclear which outcome measures or tests would be used as a reference for other papers (4). Also, there are no explicit citations for these previous studies, nor are there any power analyses carried out with previous effect

sizes (5).
It is unclear why sample sizes vary so much between studies - this should be justified (4).

Specific comments and recommendations:

4) Sample sizes are inconsistent and difficult to locate. Consider placing them all in one place (e.g., a table), with justifications for each final sample size. At the very least, the sample size for each part of the experiment should be described at the beginning of each results section.

5) Power analyses should be reported for each test.

Response:

We agree that a transparent justification for sample size is essential for reproducibility.

In the revised manuscript, we have addressed this issue in several ways. **First**, we now define a primary endpoint for each experimental module to clarify the basis for sample-size considerations. **Second**, we have now clarified our approach to sample-size selection and explicitly cited the prior literature that informed our sample-size choices in the revised **Statistical Analysis** section **(Page 41, Lines 999)**. **Third**, we report sensitivity analyses for the achieved sample sizes, which specify the minimum detectable effect size under the current sample-size configuration and help clarify which findings should be interpreted as more confirmatory versus exploratory. **Fourth**, to improve readability, we summarize the sample sizes, sensitivity-analysis results, and other key information (e.g., primary endpoints) in **Table S2 (Supplementary Information)**.

Regarding the variability in sample sizes between studies, we agree that this required clearer justification. We now explain that sample size allocation differed because the study was organized around distinct experimental aims and primary endpoints.

Specifically, the behavioral modules primarily served to validate the established CSDS- and ketamine-related phenotypes using a well-characterized endpoint (SI), whereas the electrophysiological modules (including the CSDS-related FMBFN disruption module and the ketamine-related FMBFN reconfiguration module) addressed the central mechanistic focus of the study and were therefore prioritized within the limits of recording feasibility and quality control. By contrast, the gut–brain multimodal integration module was designed as an exploratory component of the study. Consistent with this interpretation, sensitivity analysis showed that, at the achieved sample size, only relatively large correlations could be detected in the gut–

brain module (minimum detectable $|\rho| = 0.65$). We now explicitly describe these analyses as exploratory and interpret them accordingly.

Comment: Statistics are also reported only in the figure legends, which makes it difficult to locate and interpret the results of each test (6). No effect sizes are reported, which lessens the interpretability and reproducibility of the results (7). Also, exact p-values are not reported, but rather given as approximations (e.g., "$p < 0.05$" or "$p < 0.05$"), which obscures the true significance of each test statistic(8).

Specific comments and recommendations:

6) Statistics should be reported for each test in a more easily findable manner.

7) No effect sizes are reported for tests done. Report along with the other statistics.

8) Exact p-values are not reported, but rather given as approximations. Report exact p-values

Response:

6) It is agreed that statistical results should be presented in a more easily accessible manner. In the revised manuscript, statistical outcomes have been reorganized and are now reported in a clearer and more easily findable format. In addition, a comprehensive summary table has been included in the **Supplementary Data** to facilitate rapid reference.

7) It is also agreed that reporting effect sizes is essential for rigorous statistical interpretation. Accordingly, effect sizes have now been systematically reported for all statistical tests. Specifically, Cohen's d or rank-biserial correlation has been included where appropriate (**Table S2, Supplementary Information**).

8) It is acknowledged that exact p-values provide greater transparency than threshold-based reporting. Therefore, exact nominal p-values are now reported throughout the revised manuscript, replacing previous approximations. Furthermore, both nominal p-values and FDR-adjusted q-values are provided for all tests (**Table S3, Supplementary Information and Supplementary Data**).

Comment: The use of False Discovery Rate (FDR) corrections is welcome and necessary for such an exploratory study, although it is unclear why this was not used for the mediation analyses (9)

Specific comments and recommendations:

9) No FDR corrections applied to mediation analyses. Apply them as required.

Response:

Thank you for pointing out this omission. We have now addressed this by applying Benjamini–Hochberg FDR correction to the mediation analyses. For each model, we now report the indirect effect estimate, the 95% bootstrap confidence interval, the nominal P value, and the FDR-adjusted q value in the revised manuscript and **Table S3 (Supplementary Information)**. After applying this correction, several indirect effects involving *g_Colidextribacter* are no longer statistically significant. We have updated the corresponding sections of the **Results (Page 23, Lines 462-467)** and **Discussion (Page 30, Lines 646-652)** to reflect these more conservative findings.

Comment: There are multiple issues in the reporting of animal information in all studies. First, only males were included, with no explanation given as to why females were excluded (10). This limits the generalisability of the findings in this study to only males, which is an important caveat.

Specific comments and recommendations:

10) Only male rats are included. This should be justified or the experiments should be repeated with females.

Response:

Thank you for your comments. We fully recognize the importance of including both sexes to ensure the generalizability of preclinical findings. However, our decision to focus exclusively on male mice in this study was based on several fundamental methodological and biological considerations specific to the Chronic Social Defeat Stress (CSDS) paradigm:

Paradigm-specific Constraints (Territorial Aggression): The classical resident-intruder CSDS model is built upon the innate territorial aggression of CD-1 resident males, which is a naturally occurring behavior. Female mice, by contrast, typically do not exhibit territorial aggression toward same-sex conspecifics under standard conditions. While recent adaptations have been developed to induce social defeat in females—for example, by applying male urine to the female intruder to provoke aggression from a resident—these modified protocols are not yet as widely standardized or phenotypically equivalent to the "gold standard" male CSDS paradigm used here[1].

Methodological Focus of the FMBFN Framework: The primary objective of this study was to establish and validate the FMBFN framework as a novel tool for multilayer network analysis. To achieve this, it was essential to use a highly reproducible behavioral model with the well characterized pathological biomarkers. By using the standard male CSDS model, we minimized potential behavioral

variability unrelated to our framework's performance, ensuring that the observed network reorganization reflected the stress-induced pathology and ketamine's intervention.

We have expanded the **Methods section (Page 31-32, Lines 694-703)** to clarify this rationale and added a dedicated paragraph in the **Discussion (Page 31, Lines 663-681)** to explicitly acknowledge this limitation. We now state that although our findings provide a robust validation of the FMBFN framework in males, future studies utilizing sex-specific stress models will be necessary to investigate the framework's applicability to female neurobiology and to uncover potential sex-dimorphic network patterns.

Comment: Furthermore, as per ARRIVE guidelines (11) for reporting animal experiments (https://arriveguidelines.org/arrive-guidelines), as referenced in https://www.nature.com/commsbio/submit/submission-guidelines, recommendation 15, housing and husbandry details should be included - for example, were the animals in open-top cages or individually ventilated cages? Was there enrichment in the cages for animal welfare? Also, given the invasive nature of the surgery and local-field potential recording methodology, it is surprising that no information is given regarding the post-surgery care. Furthermore, no information is given as to animal attrition post-surgery. (12)

Specific comments and recommendations:

11) Refer to the ARRIVE guidelines to ensure proper reporting of animal information (https://arriveguidelines.org/arrive-guidelines).

12) Include information about animal care both during and after surgery.

Response:

Thank you for these important comments. In response, we have carefully revised the manuscript in accordance with the **ARRIVE 2.0** guidelines and substantially expanded the reporting of animal housing, husbandry, and perioperative/postoperative care. Specifically, in the revised **Methods section (Page 31-32, Lines 694-706)**, we now provide additional information on animal source, strain, age, sex, housing conditions, and welfare monitoring, including the use of individually ventilated cages, cage ventilation rate, light/dark cycle, ambient temperature, relative humidity, bedding material, and environmental enrichment. We also clarify the housing conditions prior to the CSDS procedure, including group housing for C57BL/6 mice and individual housing for CD-1 mice.

In addition, following your suggestion, we have expanded the description of surgical and post-surgical care in **Methods section (Page 34, Lines 768-774).** We

now state that mice were anesthetized with inhaled isoflurane and placed in a stereotaxic frame, that body temperature was maintained during surgery using a heating pad, and that ophthalmic ointment was applied to prevent corneal drying. We also clarify that the scalp was disinfected and that surgery was performed under aseptic conditions. After surgery, mice were monitored until full recovery from anesthesia and were subsequently checked at least once daily for wound healing, body condition, mobility, grooming, and any signs of pain or distress. Cages were maintained in a clean and easily accessible condition to reduce postoperative burden, and mice were allowed to recover for at least 7 days before behavioral testing and electrophysiological recording.

These revisions have been incorporated into the revised manuscript, primarily in the Methods section, to improve the transparency and completeness of animal reporting.

Comment: Finally, apart from in the discussion section, there are repeated instances of inappropriate or incorrect citing of previous research (13). This includes citations such as 1, 3, 5, 9, 10, 21, and 25, which link to relevant papers, yet do not directly support the accompanying claims. This may be as a result of indirect or secondary citings, as seems to be the case for citation 25. Moreover, citations such as 6, 16-19, and 48-50, for example, are certainly relevant, but more robust, exemplary, landmark papers should be cited to support such claims, if possible.

Specific comments and recommendations:

13) Incorrect or irrelevant citations, weakening the paper’s theoretical underpinnings. Revisit the following citations and reconsider their relevance: 1, 3, 5, 6, 9, 10, 16-19, 21, 25, 48-50.

Response:

Thank you for your careful check. In response, we have re-examined the citations throughout the manuscript, with particular attention to references 1, 3, 5, 6, 9, 10, 16–19, 21, 25, and 48–50, as suggested. We have removed citations that did not directly support the corresponding claims, replaced indirect or secondary citations with more appropriate primary sources wherever possible, and introduced stronger landmark references when available. **Review Table 1** shows the detail information for our revisions.

Review Table 1. Summary of citation revisions for references

Original ref. no. in previous manuscript	Action taken	New ref. no. in revised manuscript	Revised reference
1	Replace	1	World Health Organization. Comprehensive Mental Health Action Plan 2013–2030 (World Health Organization, 2021).
3	Replace	3	American Psychiatric Association. Diagnostic and Statistical Manual of Mental Disorders: DSM-5 (American Psychiatric Association, 2013).
5	Remove		
6	Replace	5	Krishnan, V. et al. Molecular Adaptations Underlying Susceptibility and Resistance to Social Defeat in Brain Reward Regions. Cell. 2007.
9	Replace	8	Berman, R. M. et al. Antidepressant effects of ketamine in depressed patients. Biological Psychiatry. 2000.
10	Replace	9	Lyu, D. et al. Ketamine induces rapid antidepressant effects via the autophagy-NLRP3 inflammasome pathway. Psychopharmacology. 2022.
16	Remove		
17	Replace	19	Liu, L. et al. Gut microbiota and its metabolites in depression: from pathogenesis to treatment. eBioMedicine. 2023.
18	Replace	21	Xie, J.-X. & Zhou, L.-W. Ketamine enantiomers and the gut microbiota: mechanisms, clinical implications, and future directions. *Mol Biol Rep*. 53, 181 (2025).
19	Replace	20	Yang, C. *et al.* Possible role of the gut microbiota–brain axis in the antidepressant effects of (R)-ketamine in a social defeat stress model. *Transl Psychiatry*. 2017.
21	Replace	16	Barabási, D. L. *et al.* Neuroscience Needs Network Science. *The Journal of Neuroscience*. 2023.
25	Remove		
48	Replace	54	Cryan, J. F. *et al.* The Microbiota-Gut-Brain Axis. Physiological Reviews. 2019.
49	Replace	55	Morais, L. H., Schreiber, H. L. & Mazmanian, S. K. The gut microbiota–brain axis in behaviour and brain disorders. Nature Reviews Microbiology. 2021.
50	Replace	56	Loh, J. S. et al. Microbiota–gut–brain axis and its therapeutic applications in neurodegenerative diseases. Signal Transduction and Targeted Therapy.2024.

Response:

As suggested, the title has been revised to: "Multilayer Brain Network Analysis in mice Reveals Ketamine-Induced Reorganization of Brain-wide Fluctuations and Gut-Brain Axis ".

Reviewer #2 (Comments to the Author):

Comment: I co-reviewed this manuscript with one of the reviewers who provided the listed reports. This is part of the Communications Biology initiative to facilitate training in peer review and to provide appropriate recognition for Early Career Researchers who co-review manuscripts.

Response:

We thank Reviewers #1 and #2 for their time and contribution to the peer-review process.

Reviewer #3 (Comments to the Author):

Comment: This manuscript presents an innovative and technically ambitious attempt to integrate behaviour, multi-region LFP-derived network physiology, and 16S rRNA gut microbiome profiling to probe ketamine's antidepressant-related effects in CSDS mice. The frequency-varying multilayer brain functional network (FMBFN) framework is clearly motivated, and the main results are interesting and consistent with existing literature. Further, the correlation between neural and gut-microbiome data is a highly relevant emerging topic for mental health treatment research. However, there are some methodological and interpretative considerations to be addressed, especially regarding the statistical approach.

Response:

Thank you for this thoughtful and encouraging evaluation of our work. We have carefully revised the manuscript to improve methodological rigor, statistical transparency, and interpretative clarity, and we address each of your specific comments in detail below.

Comments: In the materials, it is described how only the CSDS-sensitive animals were selected for the analysis. It would be good to report the number of animals that were sensitive to the behavioural test. What characteristics made certain animals sensitive, and some others not? What was the criterion and threshold approach used to select those?

Response:

We thank you for this important comment. We agree that the definition of stress susceptibility and the basis for animal selection should be described more explicitly.

In the revised **Methods section (Social Interaction Test , Page 33, Lines 742-749)**, we now clarify that susceptibility to CSDS was determined using the social interaction test (SIT). Specifically, the social index (SI) was calculated as the ratio of time spent in the interaction zone during the "With CD1" session to that during the "No CD1" session. Following widely used criteria in the CSDS literature[1,2], mice exposed to CSDS with **SI < 1** were classified as **stress-susceptible**, indicating the presence of social avoidance behavior, whereas mice with **SI ≥ 1** were classified as **stress-resilient**.

We also now clarify that the key feature distinguishing susceptible mice from resilient mice in this study was the emergence of a social avoidance phenotype after CSDS exposure, operationally defined as reduced interaction with the social target in the SIT. Only stress-susceptible mice were included in the downstream CSDS-related

analyses because the aim of the study was to characterize abnormal brain activity associated with the CSDS-induced social avoidance phenotype. In our cohort, approximately 70% of CSDS-exposed mice exhibited a susceptible phenotype.

Comments: In Figure 2 (I－Q) and related analyses, the authors report individual 5-second epochs as the basic analytic unit for the FMBFN analysis. The authors should clarify why the data were not averaged across epochs for each mouse. Further, if any inferential statistics are performed at the epoch level (or on features derived per-epoch) without accounting for repeated measures within mouse, then the analysis is pseudo-replicated and should be re-analysed using an approach that respects nesting (e.g., linear mixed-effects models with mouse as a random effect, or averaging to one value per mouse before group statistics).

Response:

Thank you for insightful comments. We agree that the inferential unit in the FMBFN analyses should have been stated more explicitly, and that repeated epochs from the same mouse should not be treated as independent biological replicates.

In our framework, 5-s epochs were used as the basic unit for constructing individual FMBFNs because the duration of occupancy within the social interaction zone varied substantially across trials and across animals. Segmenting trials into fixed-length epochs allowed us to standardize the computation of within- and cross-frequency connectivity and to improve the comparability of network estimates across recordings. However, these epochs were not intended to serve as independent units for group-level inference.

With the exception of the SVM-based classification analyses, in which each 5-s epoch was treated as one sample to increase the sample number, **all statistical comparisons were performed at the mouse level after averaging across epochs within each mouse.** To address the confusion caused by our previous wording, we have now clarified in the revised manuscript that, for the analyses and related group comparisons, epoch-derived network measures were first averaged within each mouse, and the resulting **mouse-level values** were then used for inferential statistics. Thus, each data point represents one mouse rather than one epoch. We have revised the corresponding **Methods section (Page 42-43, Lines 1010-1015)** and **figure legends** to make this point explicit.

We fully agree that treating epochs as independent observations without accounting for nesting would constitute pseudo-replication. Our revised description now makes clear that the statistical analyses were based on **mouse-level values** after within-mouse averaging, thereby respecting the nested structure of the data.

Comments: Relatedly, the SVM validation explicitly treats each 5-second epoch as one sample, pooling epochs from all mice across groups with repeated random train/test splits and a shuffle-label baseline. This design risks inflated accuracy/AUC if epochs from the same mouse appear in both the training and test sets (non-independence / data leakage), so the classification should be repeated using animal-level splitting.

Response:

Thank you for this important comment. We agree that the original epoch-level SVM framework could risk inflated classification performance if epochs from the same mouse were present in both the training and test sets. Following your suggestion, we therefore **repeated the classification analysis using animal-level splitting**, such that all epochs from each mouse were kept within a single split and never shared across training and test sets. This approach eliminates within-mouse data leakage and provides a more stringent evaluation of classification performance.

The results of the animal-level classification analysis are shown as follow (**Figure S6, Supplemental Information**). Although the classification performance decreased compared with the epoch-level analysis, the classification accuracy and AUC remained significantly higher than the shuffled-label baseline after reanalysis at the animal level. This indicates that the original results were not solely driven by non-independence among epochs. We have revised the **Methods, Results, and figure legends** accordingly, and we now describe the SVM analysis more explicitly as an animal-level validation of the discriminative information contained in the FMBFN features.

Comments: Also, for clarity and reproducibility, please standardise and explicitly label for every analysis/figure: number of mice, number of epochs/trials contributing per mouse, whether values are averaged per mouse before statistics, and whether the reported P values and ML validation are computed at the mouse level or epoch level (including how cross-validation splits were performed).

Response:

We thank you for this helpful comment. We agree that these details should be reported more explicitly for clarity and reproducibility. In the revised manuscript, we have now standardized the reporting across figure legends and explicitly indicated these information.

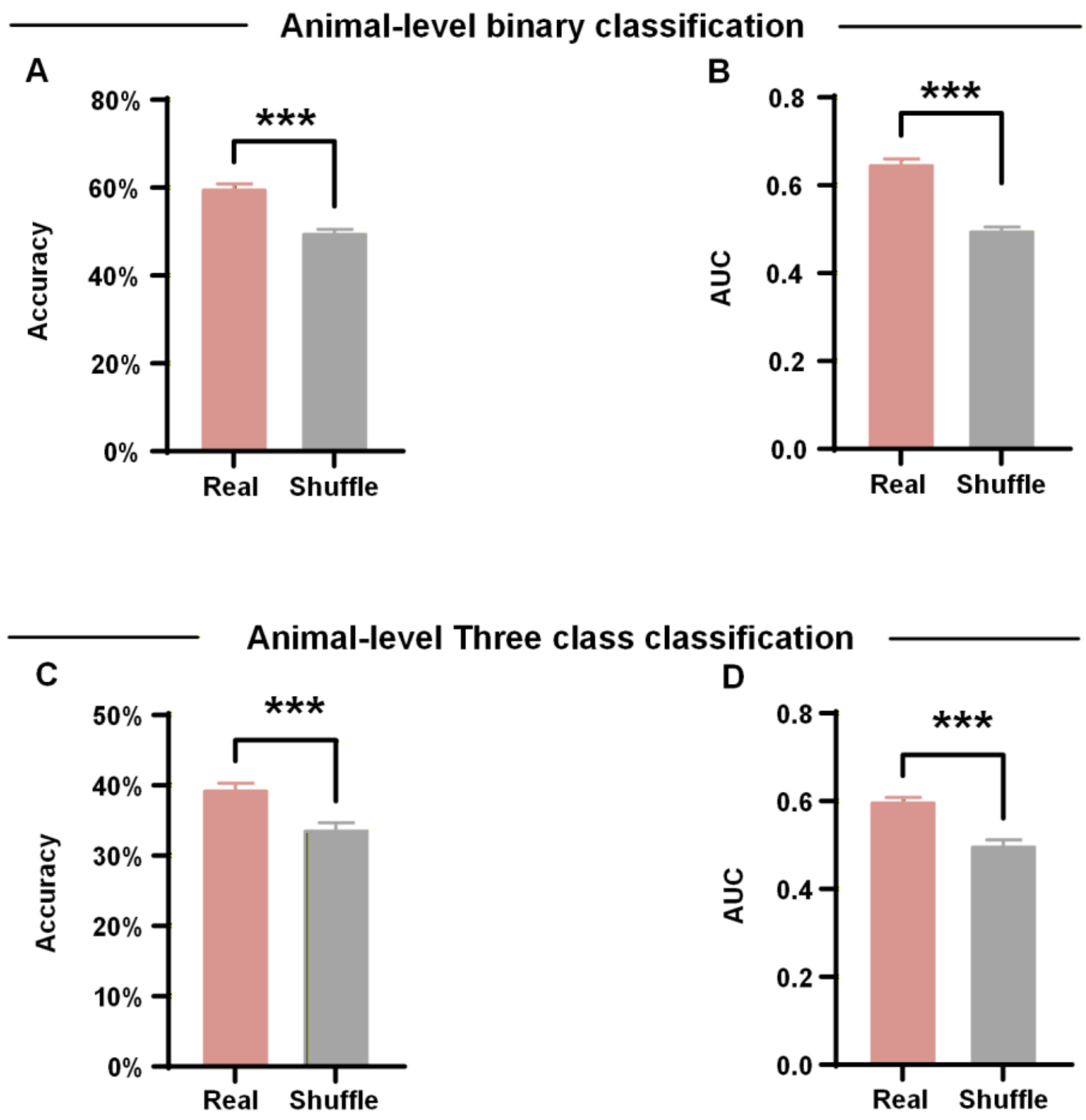

Figure S6. Animal-level classification performance based on FMBFN features (A) Binary classification accuracy for Real and Shuffle data. (Mann-Whitney test; Real 60.05% ± 1.08%; Shuffle: 50.00% ± 1.05%; U=2345.00; rank-biserial correlation (r_rb)=0.53 *** P < 0.001) (B) Binary classification AUC for Real and Shuffle data. (Mann-Whitney test; Real 0.67 ± 0.01; Shuffle: 0.50 ± 0.02; U=1930.00; rank-biserial correlation (r_rb)=0.61 *** P < 0.001). (A-B) Animal-level binary classification performance for the Con and CSDS group.Data are presented as mean ± SEM. (C) Three-class classification accuracy for Real and Shuffle data. (Mann-Whitney test; Real 39.58% ± 0.94%; Shuffle: 33.84% ± 0.98%; U=3395.00; rank-biserial correlation (r_rb)=0.32 *** P < 0.001) (D) Three-class classification AUC for Real and Shuffle data. (Mann-Whitney test; Real 0.60 ± 0.01; Shuffle: 0.50 ± 0.02; U=2550.00; rank-biserial correlation (r_rb)=0.49 *** P < 0.001). (C-D) Animal-level three class classification performance for the Con-Sal、CSDS-Sal and CSDS-Ket group. Data are presented as mean ± SEM.

Comments: The framework quantifies within- and cross-frequency coupling using the phase-lag index (PLI) after bandpass filtering and Hilbert-phase extraction, including cross-frequency PLI computed between the phase time series of different

bands/regions. Because ketamine (and stress) can robustly alter spectral power, a clearer explanation is needed of whether, and how, power differences are handled, especially for cross-frequency comparisons where band-specific power may differ between groups. This needs to be elaborated more in the methods, alongside a detailed report of filter parameters, Hilbert implementation details, edge handling, exact preprocessing steps, software packages/versions for each step, and QC metrics of the recordings.

Response:

We thank the reviewer for this insightful comment. We agree that both CSDS and ketamine can alter spectral power across multiple frequency bands. Indeed, in our previous study, we demonstrated robust changes in power spectral density following CSDS and ketamine exposure[3]. We have now clarified this point in the revised manuscript.

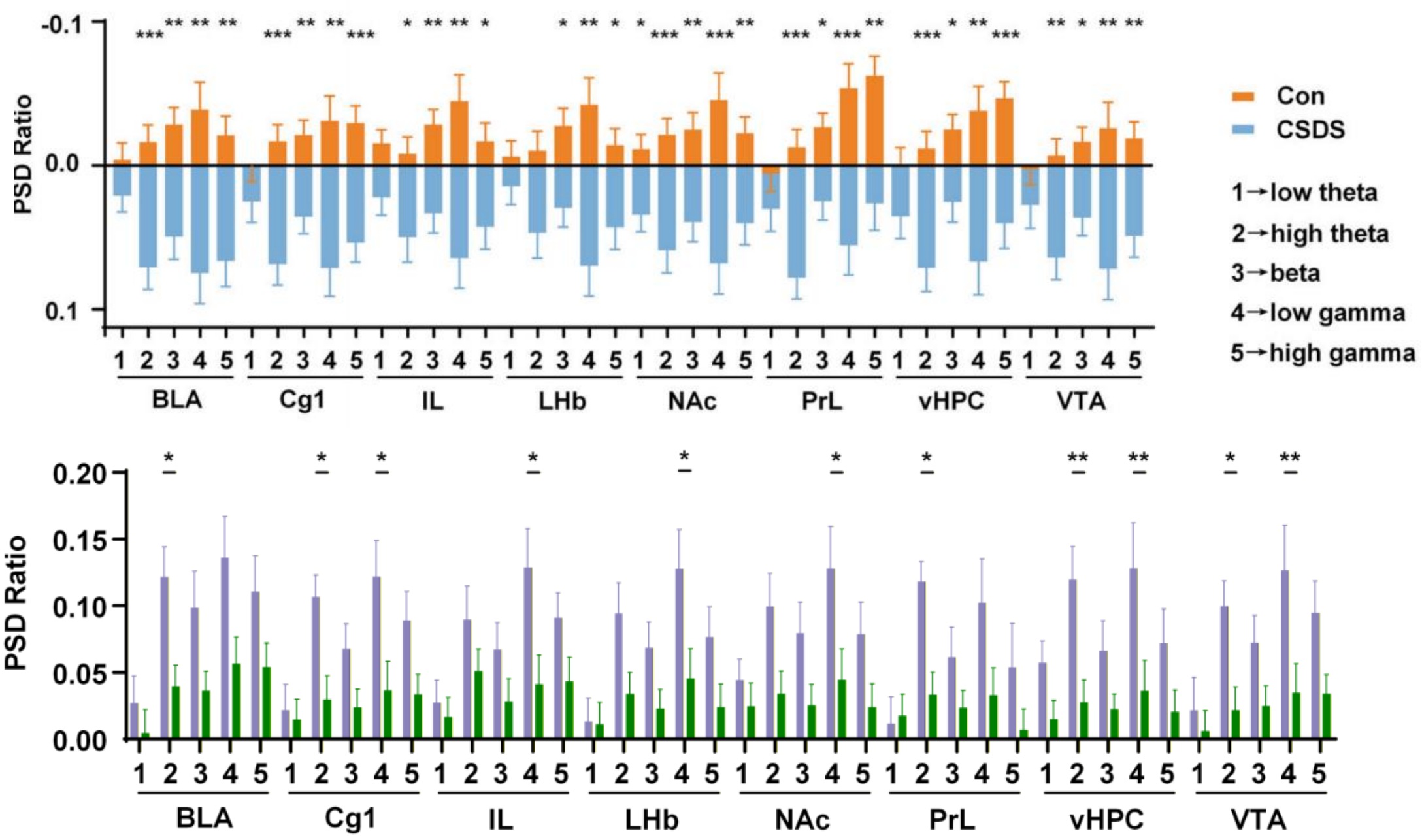

Review Figure 1. Region- and frequency-specific changes in power spectral density (PSD) ratio associated with CSDS and ketamine treatment（Figure modified from[3]）

In the present study, we quantified functional connectivity using the phase-lag index (PLI), which was selected because it is a phase-based metric computed from the consistency of non-zero phase differences and does not directly incorporate signal amplitude or spectral power into the connectivity estimate. Thus, although group differences in spectral power are expected, they do not mathematically enter into the PLI calculation itself.

At the same time, we acknowledge that changes in oscillatory power may indirectly influence the stability of phase estimation by altering signal-to-noise characteristics, which is particularly relevant for narrow-band and cross-frequency phase analyses. To minimize this potential confound, all recordings were processed using an identical preprocessing pipeline across groups, including the same artifact rejection, line-noise removal, detrending, band-specific filtering, and Hilbert-phase extraction procedures. In addition, filtering and phase extraction were performed on continuous recordings prior to epoch segmentation to reduce boundary-related artifacts, and only artifact-free data segments were included in the connectivity analysis.

We have revised the **Methods** section **(Page 35, 808-817; Page 35-36 823-828)** to clarify these points and supplemented the relevant details on preprocessing and Hilbert implementation, following your suggestions.

Comments: The manuscript defines cross-frequency connectivity (CFC) as PLI between phase time series from different frequency bands. However, this is not the common definition of CFC, which usually reflects the more well-studied metrics of phase–amplitude coupling (PAC) or amplitude–amplitude coupling. The authors should justify why cross-band PLI is an appropriate CFC metric here or add analyses with a conventional CFC method and show that key conclusions are robust using the above-mentioned statistical approaches. In addition, the Discussion requires a more neurophysiologically grounded interpretation of phase–phase CFC. What biological mechanism is proposed to underlie a change in phase-lag index between frequency bands between different regions? How is this related to more classical forms of functional connectivity?

Response:

We thank you for this insightful comment. We agree that the term cross-frequency coupling is most commonly used to refer to conventional measures such as phase–amplitude coupling (PAC) or amplitude–amplitude coupling (AAC). Our original wording was therefore not sufficiently precise, and we have revised the manuscript to clarify that the present study focuses specifically on **phase-based cross-band coupling quantified by PLI**, rather than canonical PAC. Oscillatory signals contain both phase and amplitude components[4], and within- and cross-frequency interactions can be characterized from multiple complementary perspectives, including phase–phase, amplitude–amplitude, phase–amplitude, and time-dependent coupling[5-8]. Different metrics therefore capture different aspects of neural coordination rather than interchangeable forms of the same phenomenon[9]. In

the revised manuscript, we now make this distinction more explicit **(Page 28, Lines 567-585)** .

Our choice of PLI as the metric for both within-frequency and cross-band interactions was motivated by two considerations. First, previous multilayer network studies[10] have systematically compared different combinations of within- and cross-frequency coupling metrics and showed that phase-based measures are informative candidates for multilayer network construction. Although PAC-based cross-frequency measures also yielded strong effects in that work, phase-based combinations showed excellent decoding performance and supported the feasibility of phase-based inter-layer coupling in a multilayer framework. Second, in our own exploratory analyses, we compared several candidate CFC metrics, including modulation index (MI) for PAC, amplitude envelope correlation (AEC) for amplitude–amplitude coupling, and PLI for phase–phase coupling. In our dataset, PLI was the most sensitive to group-related cross-band differences and was therefore selected for the primary network analysis. The Statistical comparison results for MI and AEC were shown as follow.

Importantly, our exploratory MI results showed partially convergent patterns with the PLI-based findings. For example, both metrics indicated stronger coupling involving the higher-frequency IV–V bands in the CSDS-Ket group relative to the Con-Sal group, and both detected significant interactions between band I and higher-frequency bands, although the direction of these differences was not identical across metrics. These observations suggest that the PLI-based cross-band results are not isolated findings, but rather capture one aspect of a broader reorganization of cross-band interactions.

To address your request for a more neurophysiologically grounded interpretation, we have expanded the **Discussion (Page 28, Lines 575-585)**. Specifically, we now clarify that a change in cross-band PLI does not imply canonical phase–amplitude nesting or a direct causal interaction between frequencies. Rather, it indicates that the stability of non-zero phase relationships between oscillatory activities operating at different timescales has changed across distributed brain regions. One plausible biological interpretation is that oscillatory phase alignment helps regulate inter-areal communication by aligning windows of neuronal excitability[11-13]; from this perspective, altered cross-band PLI may reflect altered temporal coordination of neuronal communication across timescales. In our data, the increased II–III cross-frequency PLI observed in the CSDS group may therefore indicate abnormally rigid cross-timescale locking or maladaptive hypersynchronization, which is consistent with the concomitant increase in global integration and reduced network

flexibility. Conversely, ketamine-related reductions in cross-frequency PLI may reflect a release from this rigid temporal locking and a shift toward more flexible network communication.

We have also clarified how this measure relates to more classical forms of functional connectivity. Within-frequency connectivity captures coordination within the same oscillatory timescale, whereas PAC quantifies nested interactions between the phase of a slower rhythm and the amplitude of a faster rhythm. By contrast, our PLI-based cross-band measure captures the stability of inter-regional timing relationships across different oscillatory channels. It should therefore be viewed as complementary to, but not interchangeable with, classical within-frequency functional connectivity or PAC/AAC-based cross-frequency coupling.

To avoid overextending the analytical scope of the manuscript, these additional exploratory comparisons were not included in the main text. However, we now acknowledge this issue more explicitly in the revised Discussion. We have also revised the terminology throughout the manuscript to distinguish our phase-based cross-band PLI measure from conventional PAC-based CFC.

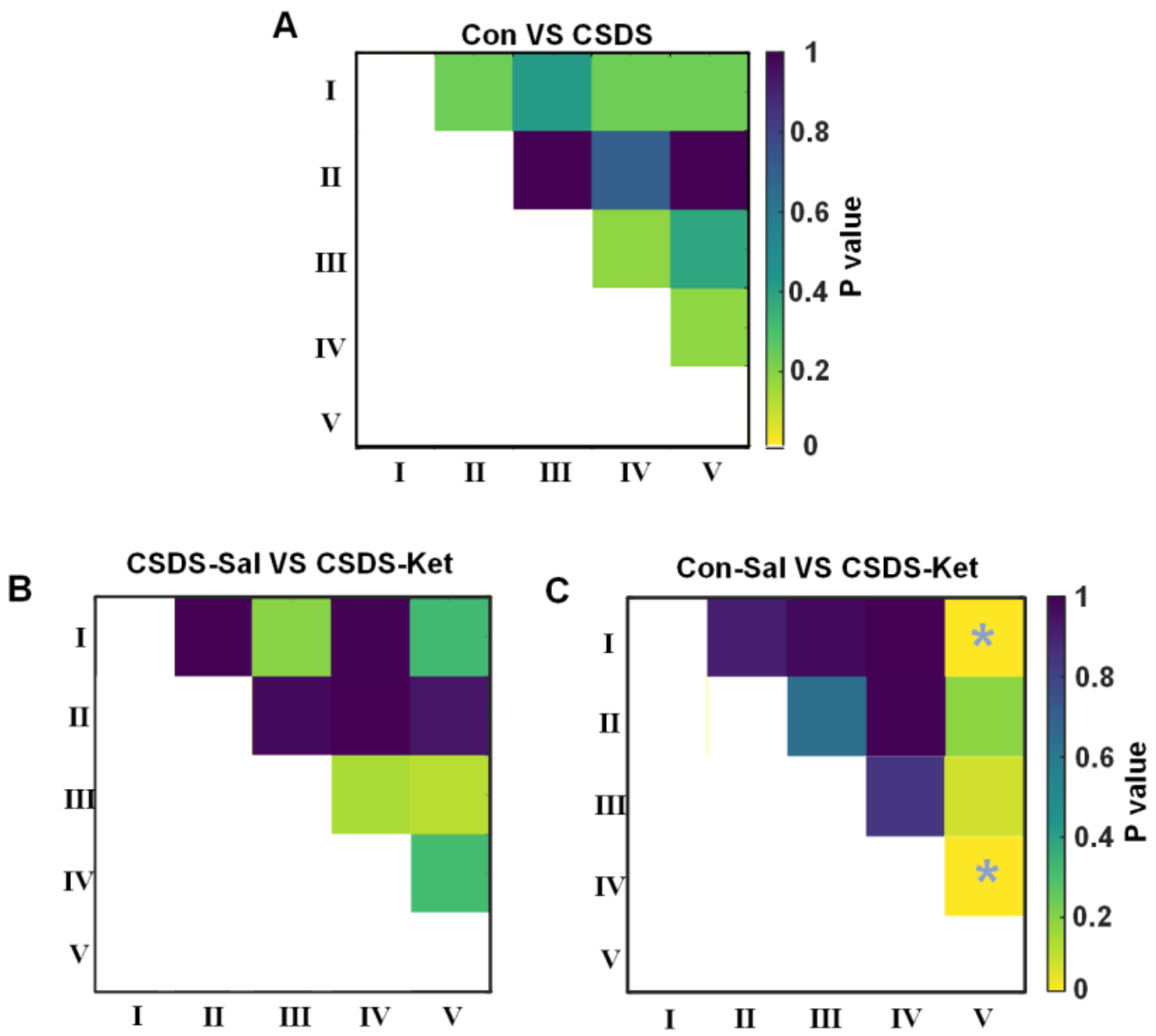

Review Figure 2. Exploratory group comparisons of MI-based cross-frequency coupling strength. Heatmaps show the P values for between-group comparisons of modulation index (MI)-based coupling strength across frequency-band pairs. (A) Group comparison between the Con and CSDS groups (Con, n=35 mice; CSDS, n=36 mice). (B) Group comparison between the CSDS-Sal

and CSDS-Ket groups. (C) Group comparison between the Con-Sal and CSDS-Ket groups. Asterisks mark band pairs that reached statistical significance. (B-C) For Con-Sal group: n= 24 mice; for CSDS-Sal group: n= 21 mice; for CSDS-Ket group: n = 21 mice. All P values were corrected for multiple testing using the Benjamini–Hochberg false discovery rate (FDR) procedure. Overall, the MI-based exploratory results showed partially convergent patterns with the PLI-based analysis, including group-dependent alterations involving higher-frequency band interactions and coupling between low- and high-frequency bands. Detailed statistical methods and full statistical results are provided in **Review Table 2** and **Review Table 3**.

Review Table 2. Summary of exploratory MI-based cross-frequency coupling statistics for Con vs CSDS

Frequency bands	Con group	CSDS group	Normal P value	FDR-adjusted q value
I–II	0.0014 ± 0.00017	0.0015 ± 0.00033	0.1112	0.2224
I–III	0.0026 ± 0.00026	0.0026 ± 0.00029	0.2822	0.4032
I–IV	0.0024 ± 0.00032	0.0024 ± 0.00035	0.0965	0.2224
I–V	0.0027 ± 0.00041	0.0028 ± 0.00075	0.0989	0.2224
II–III	0.0012 ± 0.00019	0.0012 ± 0.00025	0.9862	0.9862
II–IV	0.00072 ± 4.6e-05	0.00072 ± 8.5e-05	0.5536	0.6921
II–V	0.0016 ± 0.0004	0.0017 ± 0.00065	0.9587	0.9862
III–IV	0.00023 ± 1.3e-05	0.00024 ± 3.1e-05	0.0293	0.1692
III–V	0.00036 ± 6.1e-05	0.00049 ± 0.00036	0.2206	0.3677
IV–V	7.6e-05 ± 4.4e-06	7.4e-05 ± 7.3e-06	0.0338	0.1692

Review Table 3. Summary of exploratory MI-based cross-frequency coupling statistics for ketamine-related group comparisons

Frequency bands	Con-Sal group	CSDS-Sal group	CSDS-Ket group	Normal P value	FDR adjusted q value	Post hoc results(adjusted P value)
I–II	0.0015 ± 0.0002	0.0015 ± 0.00036	0.0015 ± 0.00024	0.8226	0.9548	Con-Sal vs CSDS-Sal: adj p = 0.9547 (ns); Con-Sal vs CSDS-Ket: adj p = 0.9130 (ns); CSDS-Sal vs CSDS-Ket: adj p = 0.9991 (ns)
I–III	0.0027 ± 0.0003	0.0025 ± 0.0003	0.0029 ± 0.00063	0.1488	0.2602	Con-Sal vs CSDS-Sal: adj p = 0.3548 (ns); Con-Sal vs CSDS-Ket: adj p = 0.9659 (ns); CSDS-Sal vs CSDS-Ket: adj p = 0.1813 (ns)
I–IV	0.0024 ± 0.00028	0.0023 ± 0.00035	0.0026 ± 0.00091	0.9548	0.9548	Con-Sal vs CSDS-Sal: adj p = 0.9875 (ns); Con-Sal vs CSDS-Ket: adj p = 0.9998 (ns); CSDS-Sal vs CSDS-Ket: adj p = 0.9949 (ns)
I–V	0.0029 ± 0.00063	0.0028 ± 0.00066	0.0025 ± 0.00032	0.0187	0.0934	Con-Sal vs CSDS-Sal: adj p = 0.5440 (ns); Con-Sal vs CSDS-Ket: adj p = 0.0144 (sig); CSDS-Sal vs CSDS-Ket: adj p = 0.3116 (ns)
II–III	0.0013 ± 0.00023	0.0012 ± 0.00027	0.0012 ± 0.00014	0.5614	0.8021	Con-Sal vs CSDS-Sal: adj p = 0.8976 (ns); Con-Sal vs CSDS-Ket: adj p = 0.6375 (ns); CSDS-Sal vs CSDS-Ket: adj p = 0.9641 (ns)
II–IV	0.00072 ± 5.8e-05	0.00072 ± 6.6e-05	0.00073 ± 5.5e-05	0.933	0.9548	Con-Sal vs CSDS-Sal: adj p = 0.9764 (ns); Con-Sal vs CSDS-Ket: adj p = 0.9943 (ns); CSDS-Sal vs CSDS-Ket: adj p = 0.9987 (ns)
II–V	0.002 ± 0.00075	0.0018 ± 0.00074	0.0016 ± 0.0003	0.1561	0.2602	Con-Sal vs CSDS-Sal: adj p = 0.4671 (ns); Con-Sal vs CSDS-Ket: adj p = 0.1768 (ns); CSDS-Sal vs CSDS-Ket: adj p = 0.9339 (ns)
III–IV	0.00023 ± 2e-05	0.00024 ± 3.4e-05	0.00023 ± 2.3e-05	0.123	0.2602	Con-Sal vs CSDS-Sal: adj p = 0.4500 (ns); Con-Sal vs CSDS-Ket: adj p = 0.8392 (ns); CSDS-Sal vs CSDS-Ket: adj p = 0.1253 (ns)
III–V	0.00045 ± 0.00018	0.00049 ± 0.00031	0.00035 ± 4.4e-05	0.0439	0.1464	Con-Sal vs CSDS-Sal: adj p = 0.9997 (ns); Con-Sal vs CSDS-Ket: adj p = 0.0720 (ns); CSDS-Sal vs CSDS-Ket: adj p = 0.1039 (ns)
IV–V	7.7e-05 ± 4.6e-06	7.5e-05 ± 7.4e-06	7.2e-05 ± 5.3e-06	0.0053	0.0497	Con-Sal vs CSDS-Sal: adj p = 0.2929 (ns); Con-Sal vs CSDS-Ket: adj p = 0.0036 (sig); CSDS-Sal vs CSDS-Ket: adj p = 0.3029 (ns)

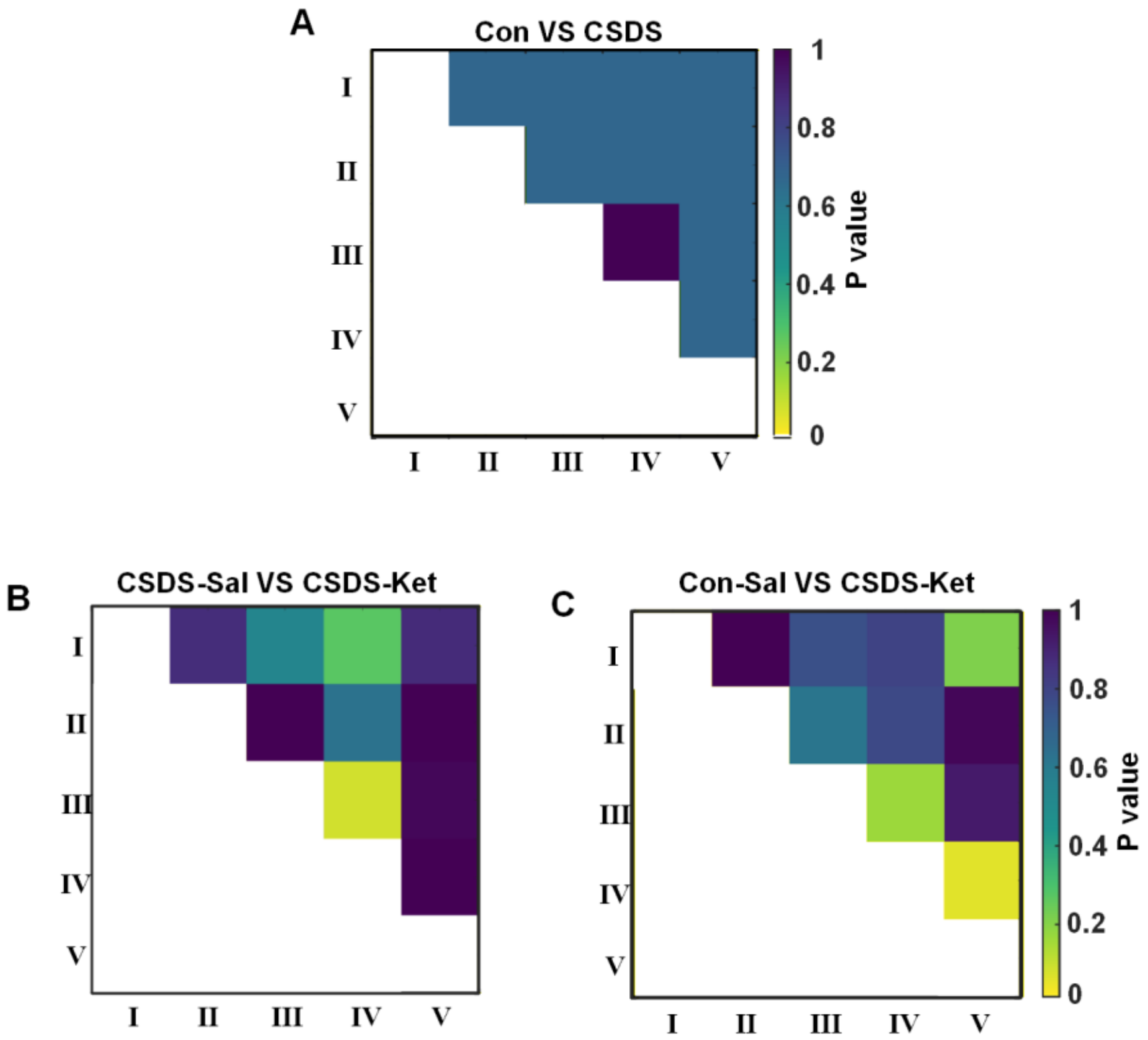

Review Figure 3. Exploratory group comparisons of AEC-based cross-frequency coupling strength. Heatmaps show the P values for between-group comparisons of AEC-based coupling strength across frequency-band pairs. (A) Group comparison between the Con and CSDS groups (Con, n=35 mice; CSDS, n=36 mice). (B) Group comparison between the CSDS-Sal and CSDS-Ket groups. (C) Group comparison between the Con-Sal and CSDS-Ket groups. Asterisks mark band pairs that reached statistical significance. (B-C) For Con-Sal group: n= 24 mice; for CSDS-Sal group: n= 21 mice; for CSDS-Ket group: n = 21 mice. All P values were corrected for multiple testing using the Benjamini–Hochberg false discovery rate (FDR) procedure. Detailed statistical methods and full statistical results are provided in Review Table 4 and Review Table 5.

Review Table4. Summary of exploratory AEC-based cross-frequency coupling statistics for Con vs CSDS

Frequency bands	Con group	CSDS group	Normal P value	FDR-adjusted q value
I–II	0.14 ± 0.0085	0.16 ± 0.044	0.1918	0.6675
I–III	0.086 ± 0.005	0.096 ± 0.026	0.2979	0.6675
I–IV	0.094 ± 0.0055	0.10 ± 0.017	0.0775	0.6675
I–V	0.08 ± 0.0081	0.085 ± 0.018	0.4723	0.6675
II–III	0.092 ± 0.0059	0.099 ± 0.018	0.2621	0.6675
II–IV	0.092 ± 0.0035	0.094 ± 0.011	0.6008	0.6675
II–V	0.076 ± 0.005	0.078 ± 0.011	0.5691	0.6675
III–IV	0.10 ± 0.012	0.10 ± 0.02	0.9954	0.9954
III–V	0.066 ± 0.003	0.069 ± 0.0098	0.5928	0.6675
IV–V	0.068 ± 0.0055	0.071 ± 0.012	0.4044	0.6675

Review Table 5. Summary of exploratory AEC-based cross-frequency coupling statistics for ketamine-related group comparisons

Frequency bands	Con-Sal group	CSDS-Sal group	CSDS-Ket group	Normal P value	FDR adjusted q value	Post hoc results(adjusted P value)
I–II	0.15 ± 0.0099	0.15 ± 0.021	0.15 ± 0.015	0.6607	0.8259	Con-Sal vs CSDS-Sal: adj p = 0.7663 (ns); Con-Sal vs CSDS-Ket: adj p = 0.9978 (ns); CSDS-Sal vs CSDS-Ket: adj p = 0.8693 (ns)
I–III	0.088 ± 0.0053	0.09 ± 0.016	0.091 ± 0.0091	0.4489	0.7609	Con-Sal vs CSDS-Sal: adj p = 0.9735 (ns); Con-Sal vs CSDS-Ket: adj p = 0.7512 (ns); CSDS-Sal vs CSDS-Ket: adj p = 0.5255 (ns)
I–IV	0.097 ± 0.0089	0.095 ± 0.0089	0.099 ± 0.011	0.2481	0.6203	Con-Sal vs CSDS-Sal: adj p = 0.7442 (ns); Con-Sal vs CSDS-Ket: adj p = 0.7921 (ns); CSDS-Sal vs CSDS-Ket: adj p = 0.2589 (ns)
I–V	0.087 ± 0.017	0.086 ± 0.02	0.079 ± 0.0062	0.1877	0.6203	Con-Sal vs CSDS-Sal: adj p = 0.6239 (ns); Con-Sal vs CSDS-Ket: adj p = 0.1977 (ns); CSDS-Sal vs CSDS-Ket: adj p = 0.8738 (ns)
II–III	0.092 ± 0.0068	0.098 ± 0.015	0.099 ± 0.017	0.4566	0.7609	Con-Sal vs CSDS-Sal: adj p = 0.6630 (ns); Con-Sal vs CSDS-Ket: adj p = 0.6073 (ns); CSDS-Sal vs CSDS-Ket: adj p = 0.9999 (ns)
II–IV	0.092 ± 0.0035	0.091 ± 0.008	0.093 ± 0.0052	0.6302	0.8259	Con-Sal vs CSDS-Sal: adj p = 0.9541 (ns); Con-Sal vs CSDS-Ket: adj p = 0.7752 (ns); CSDS-Sal vs CSDS-Ket: adj p = 0.6206 (ns)
II–V	0.076 ± 0.0054	0.076 ± 0.0086	0.075 ± 0.0037	0.9336	0.9336	Con-Sal vs CSDS-Sal: adj p = 0.9888 (ns); Con-Sal vs CSDS-Ket: adj p = 0.9808 (ns); CSDS-Sal vs CSDS-Ket: adj p = 0.9999 (ns)
III–IV	0.096 ± 0.0075	0.097 ± 0.014	0.12 ± 0.04	0.0515	0.2577	Con-Sal vs CSDS-Sal: adj p = 0.9653 (ns); Con-Sal vs CSDS-Ket: adj p = 0.1506 (ns); CSDS-Sal vs CSDS-Ket: adj p = 0.0700 (ns)
III–V	0.067 ± 0.0035	0.067 ± 0.0058	0.067 ± 0.0038	0.838	0.9311	Con-Sal vs CSDS-Sal: adj p = 0.8304 (ns); Con-Sal vs CSDS-Ket: adj p = 0.9233 (ns); CSDS-Sal vs CSDS-Ket: adj p = 0.9783 (ns)
IV–V	0.066 ± 0.0035	0.071 ± 0.013	0.07 ± 0.0074	0.0432	0.2577	Con-Sal vs CSDS-Sal: adj p = 0.1195 (ns); Con-Sal vs CSDS-Ket: adj p = 0.0746 (ns); CSDS-Sal vs CSDS-Ket: adj p = 0.9979 (ns)

Comments: The paper indicates Benjamini–Hochberg FDR control for multiple comparisons in correlation contexts and that figure P values are FDR-corrected. Given the large number of connectivity matrices, many global and local topology metrics, and multiple regions, it would be important to define the exact “families” of tests entering each FDR correction (ideally in a supplementary table), especially because network metrics are not independent.

Response:

Thank you for this important comment. We agree that, given the large number of connectivity matrices, global and local topological metrics, and multiple region-wise comparisons, it is essential to define explicitly the exact families of tests included in each Benjamini–Hochberg FDR correction. To address this point, we have now summarized the key statistical information in **Supplementary Data**, including the definitions of the corresponding FDR families for each analysis.

Comments: The binarisation process to extrapolate the network metrics is described in the methods. However, a sensitivity analysis across thresholds should be reported, including network edge densities by layer and group, and show that topological effects persist after network edge density-matching.

Response:

Thank you for this important comment. We agree that the interpretation of binary network topology requires careful assessment of the potential influence of edge density.

First, we would like to clarify that our primary binarization procedure was not based on an arbitrarily selected global threshold. Instead, each edge was binarized using a surrogate-data-based, data-driven significance criterion, in which the observed connectivity value was compared against the corresponding surrogate distribution for that specific connection. Thus, the original adjacency matrices were generated without imposing a manually chosen common threshold across the entire multilayer network.

However, we also agree that even with this data-driven approach, the resulting binary topology may still be influenced by differences in edge density. We therefore performed additional analyses to directly address this issue. Specifically, we first quantified edge density separately for each within-frequency and cross-frequency functional connectivity matrix set and compared these densities across groups **(Figure S3, Supplemental Information)**. These analyses showed that in the Con vs CSDS comparison, matrix-wise edge densities did not differ significantly between groups, whereas in the Con-Sal / CSDS-Sal / CSDS-Ket comparison, the CSDS-Ket group showed density differences in several frequency matrix.

We did not implement a conventional "across-thresholds" analysis using a single common threshold applied to the full super-adjacency matrix, because this is not well suited to the present multilayer network structure. In our data, sparsity differed markedly not only between within-frequency and cross-frequency matrix sets, but also across individual frequency Matrices within each category. For example, within-frequency Matrices were substantially denser than cross-frequency Matrices, and substantial heterogeneity was also present among different within-frequency and cross-frequency Matrices themselves. Under these conditions, applying one global threshold across the entire multilayer matrix would distort the native sparsity structure of the network, disproportionately affecting some Matrices while over- or under-representing others.

To examine whether the key conclusions from our global topology analysis were driven primarily by differences in edge density, we performed a matrix-wise density-controlled analysis (**Figure S4, Supplemental Information**). For each comparison (two-group or three-group), binary networks were reconstructed using a common matrix-specific edge density derived from the observed densities of the groups under comparison (implemented as the mean edge density for that matrix across the compared groups). This approach allowed us to preserve the intrinsic sparsity differences among frequency Matrices while removing between-group edge-density differences as a confounding factor.

These additional analyses refined our interpretation. In the Con vs CSDS comparison, the previously observed differences in WML and globalWML disapear after density control, consistent with the known dependence of path-length-related metrics on edge density, whereas the ME difference remained significant. Thus, the main conclusion that CSDS selectively affects functional integration while largely sparing functional segregation remained supported, with ME providing a density-robust evidence. In the Con-Sal / CSDS-Sal / CSDS-Ket comparison, ketamine-related differences remained evident in topology metrics (MPC, MCC) that were not altered by CSDS alone, supporting our main conclusion that ketamine induces an additional reorganization of multilayer network architecture rather than simply nomalized it.

We have added the corresponding edge-density results, density-controlled analyses, and revised interpretations to the Result sections of the revised manuscript.

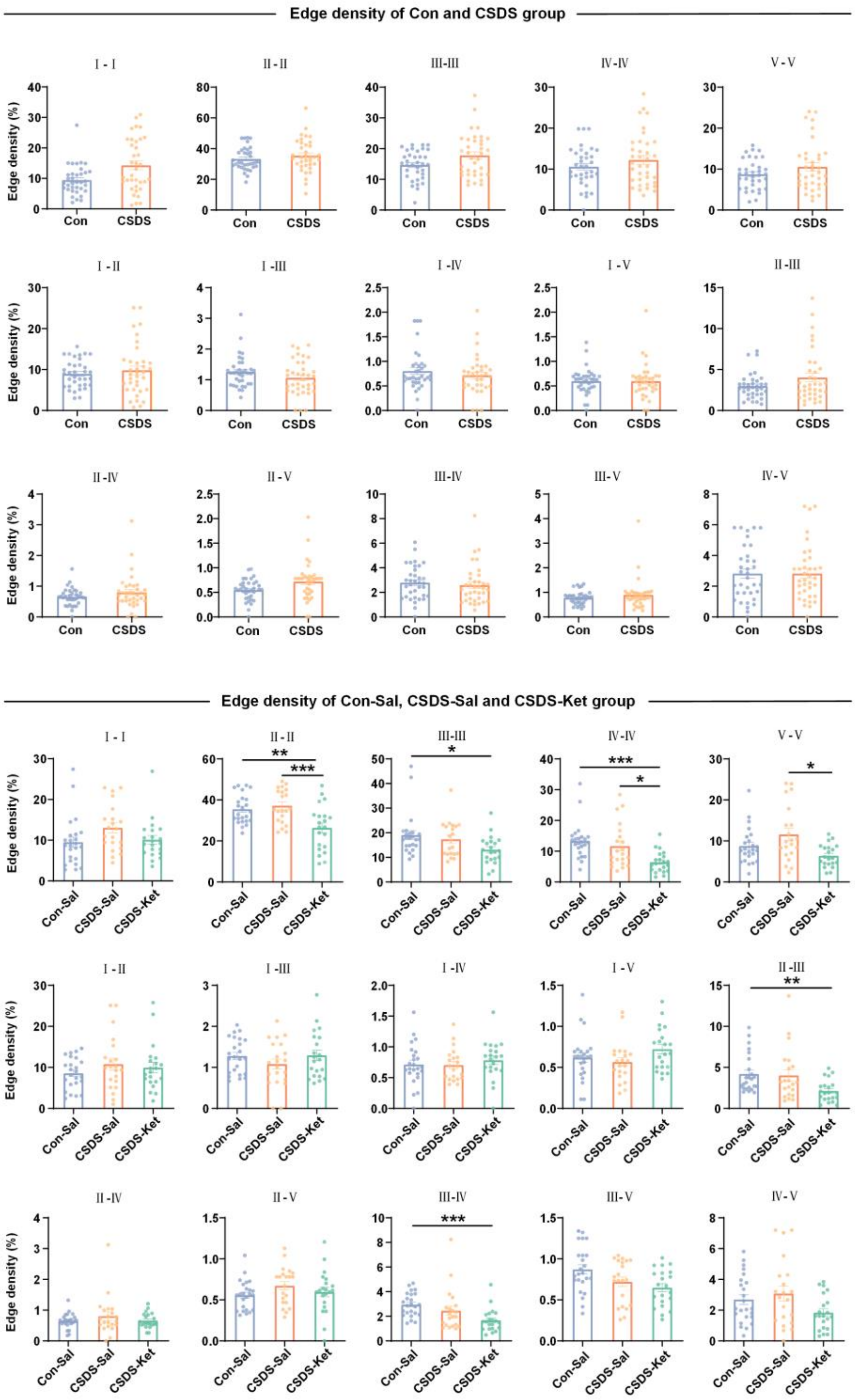

Figure S3. Edge density analysis (A) Edge density analysis for Con and CSDS group. (B) Edge density analysis for Con-Sal, CSDS-Sal and CSDS-Ket group. (A-B) Animal-level binary classification performance. Data are presented as mean ± SEM. All P values were corrected for multiple testing using the Benjamini–Hochberg false discovery rate (FDR) procedure. Detailed statistical methods and full statistical results are provided in Supplementary Data.

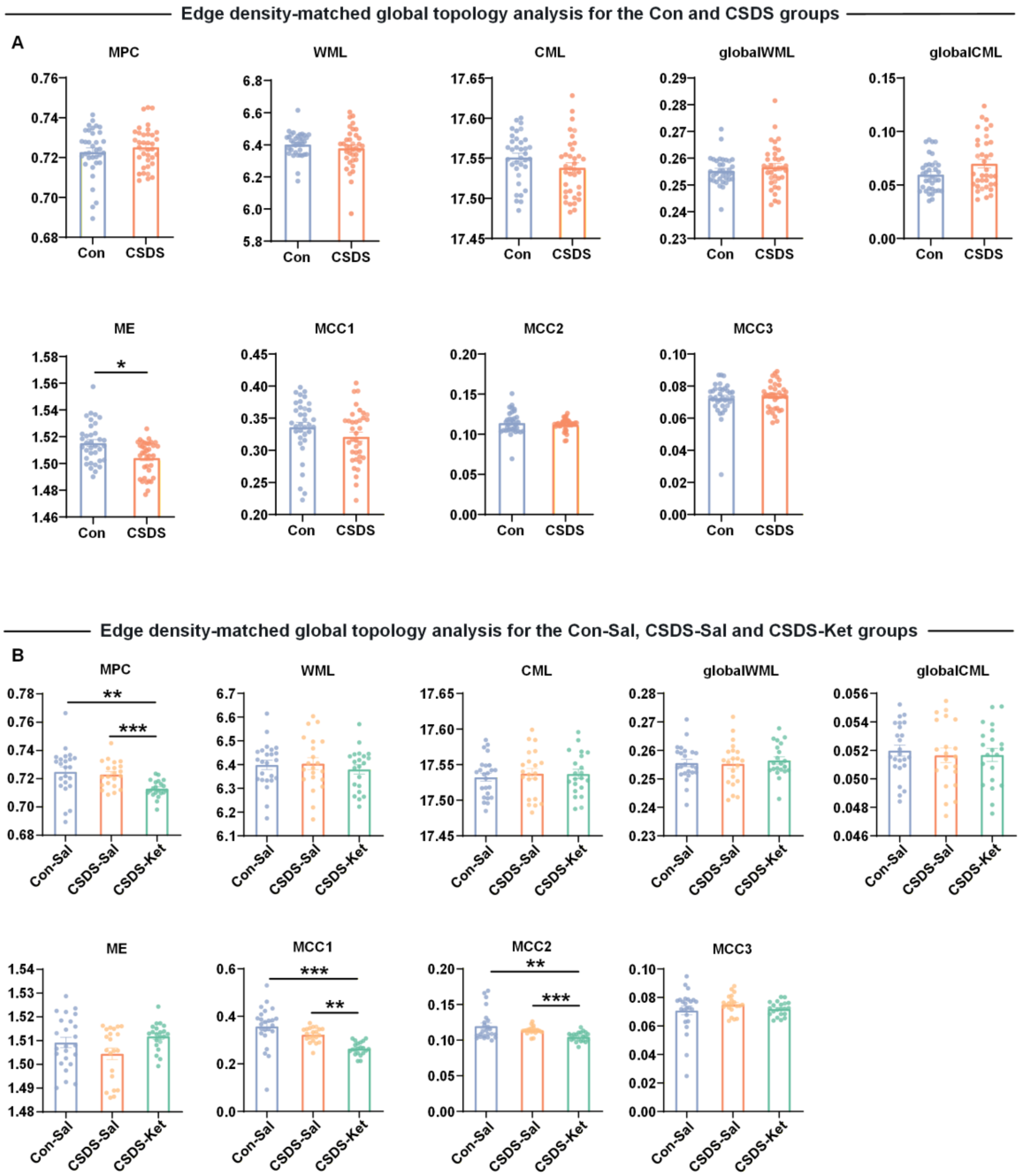

Figure S4. Edge density-matched global topology analysis (A) Edge density-matched global topology analysis for Con and CSDS group. (B) Edge density-matched global topology analysis for Con-Sal, CSDS-Sal and CSDS-Ket group. (A-B) Animal-level binary classification performance. Data are presented as mean ± SEM. All P values were corrected for multiple testing using the Benjamini–Hochberg false discovery rate (FDR) procedure. Detailed statistical methods and full statistical results are provided in **Supplementary Data**.

Comments: The manuscript reports "bidirectional mediation" between ketamine-altered microbiome genera and network metrics and suggests potential causal insights, with microbiome group sizes of n=5 per group. With such a small n

and an observational design, a mediation analysis is hardly justified. The Discussion should more explicitly treat mediation as exploratory and hypothesis-generating (not causal proof) and outline concrete causal follow-up experiments that would test directionality. It would also be important to give a physiological interpretation of such a correlation. Why was the correlation expected, and what are the mechanisms that underlie it?

Response:

Thank you for this thoughtful and important comment. We agree that, given the limited microbiome sample size (n = 5 per group) and the observational nature of the study, the mediation analyses should not be interpreted as evidence of causality. Accordingly, we have substantially softened the relevant wording throughout the **Results (Page 23, Lines 462-470)** and **Discussion (Page 30, Lines 646-652)**. Specifically, we no longer describe these findings as providing causal insight or as demonstrating a true bidirectional feedback loop. Instead, we now explicitly present the mediation analyses as **exploratory and hypothesis-generating**, intended to identify candidate gut–brain association patterns for future validation. We have also expanded the **Discussion** to provide a physiological rationale for why such correlations might be expected **(Page 30, Lines 652-662)**.

Comments: Please add a clear limitation section. For instance, the generalisability of the finding is limited given that only male mice were used and there are known sex differences in stress susceptibility, neurophysiology, and ketamine response. Further, the limited sample size makes the correlations exploratory as they don't seem justified by power calculations, especially the one between neural and gut microbiome data.

Response:

Thank you for this helpful suggestion. We agree and have added a clear **limitation section** to the revised **Discussion (Page 30, Lines 663-681)**. Specifically, we now state that the use of only male mice limits the generalisability of our findings, particularly in light of known sex differences in stress susceptibility, neurophysiology, and ketamine response. We also clarify that the microbiome-related analyses, especially the neural–microbiome correlation and mediation analyses, were based on a limited sample size and should therefore be interpreted as exploratory and hypothesis-generating rather than confirmatory. In addition, we note that these analyses do not support causal inference and will require validation in larger and experimentally controlled studies.

Comments: Minor comments

- It would have been interesting to show whether the effect of ketamine was specific to the CSDS mice as compared to WT, given that additional ketamine-induced neurophysiological effects were observed beyond reversing the CSDS phenotype. If any data on ketamine effects in WT or CSDS mice are available would be good to report them as well.

Response:

Thank you for this comment. We agree that it is important to determine whether the neurophysiological effects of ketamine are specific to CSDS mice or are also present in WT/control animals, particularly because several ketamine-related changes appear to extend beyond a simple normalization of the CSDS phenotype. This is indeed an important point and a valuable suggestion. In the current study, our primary objective was to examine ketamine-associated network reorganization within the context of stress-induced pathology. We agree that the inclusion of ketamine-treated WT/control mice would help to distinguish disease-state rescue effects from ketamine-specific physiological effects. Although such data are not available in the present study, your comment is highly informative and will provide important guidance for our future work.

Comments:- The sentence "To transform complex microbial community data into well-defined features for integration with FMBFN analytical framework." is out of place and reads like an editing fragment (around lines 469－471).

- Typo/heading issue: "Topological properties of functional segregation" appears to introduce integration metrics (participation coefficient, path length, efficiency, entropy), so the heading should likely be "integration," not "segregation" (around line ~885).

- Additional copyedits spotted in Methods: "Illumina MiSeq sequencing were were conducted…" (duplicate "were").

Response:

Thank you for these careful comments. We agree that the quoted sentence was misplaced and appeared as an editing fragment, and we have revised it accordingly. We have also corrected the heading referring to "functional segregation," as the section mainly introduces integration-related metrics, and we have fixed the typographical error in the Methods section ("were were"). All of these issues have all been corrected in the revised manuscript.

References

1. Golden, SA., Covington, HE., Berton, O. & Russo, SJ. A standardized protocol for repeated social defeat stress in mice. *Nat Protoc*. **6**, 1183-1191 (2011).
2. Morel, C. *et al.* Midbrain projection to the basolateral amygdala encodes anxiety-like but not depression-like behaviors. *Nat Commun*. **13**, 1532 (2022).
3. Cao, Q. *et al.* Mesoscale brain-wide fluctuation analysis: revealing ketamine's rapid antidepressant across multiple brain regions. *Transl Psychiatry*. **15**, 155 (2025).
4. Gwon, D. & Ahn, M. Alpha and high gamma phase amplitude coupling during motor imagery and weighted cross-frequency coupling to extract discriminative cross-frequency patterns. *NeuroImage*. **240**, 118403 (2021).
5. Yeh, CH. *et al.* Cross-Frequency Coupling and Intelligent Neuromodulation. *Cyborg Bionic Syst*. **4**, 0034 (2023).
6. Siems, M. & Siegel, M. Dissociated neuronal phase- and amplitude-coupling patterns in the human brain. *NeuroImage*. **209**, 116538 (2020).
7. Benschop, L. *et al.* Electrophysiological scarring in remitted depressed patients: Elevated EEG functional connectivity between the posterior cingulate cortex and the subgenual prefrontal cortex as a neural marker for rumination. *J Affect Disord*. **281**, 493-501 (2021).
8. Charalambous, E. & Djebbara, Z. On natural attunement: Shared rhythms between the brain and the environment. *Neurosci Biobehav Rev*. **155**, 105438 (2023).
9. Cai, H. *et al.* A multi-modal open dataset for mental-disorder analysis. *Sci Data*. **9**, 178 (2022).
10. Qu, S. *et al.* Depression recognition using high-order generalized multilayer brain functional network fused with EEG multi-domain information. *Information Fusion*. **114,** 102723 (2025).
11. Fries, PA mechanism for cognitive dynamics: neuronal communication through neuronal coherence. *Trends Cogn Sci*. **9**, 474-480 (2005).
12. Fries, P. Rhythms for Cognition: Communication through Coherence. *Neuron*. **88**, 220-235 (2015).
13. Canolty, RT. & Knight, RT. The functional role of cross-frequency coupling. *Trends Cogn Sci*. **14**, 506-515 (2010).

Revision explanation on manuscript COMMSBIO-25-10210A

Title: Multilayer Brain Network Analysis in Mice Reveals Ketamine-Induced Reorganization of Brain- wide Fluctuations and Gut-Brain Axis

Dear Editor and Reviewers,

We sincerely thank the Editor and Reviewers for their positive evaluation of our revised manuscript. Following the editorial request, we carefully reviewed the manuscript and associated files and made the necessary revisions to comply with the journal's formatting and submission requirements. Minor editorial refinements were also made to further improve clarity and readability.

Reviewer #1 (Comments to the Author):

Comment: This resubmission adequately addressed all of our comments - although we feel the paper remains rather long and complex - potentially difficult for readers to digest.

Response:

We thank the reviewer for the positive assessment. No further scientific comments were raised. Minor editorial refinements have been made to improve readability.

Reviewer #2(Comments to the Author):

Comment: I co-reviewed this manuscript with one of the reviewers who provided the listed reports. This is part of the Communications Biology initiative to facilitate training in peer review and to provide appropriate recognition for Early Career Researchers who co-review manuscripts.

Response:

We thank the reviewer for the evaluation and support.

Reviewer #3Comments to the Author):

Comment: Thank you for your thorough and careful revision of the manuscript "Multilayer Brain Network Analysis in Mice Reveals Ketamine-Induced Reorganization of Brain-wide Fluctuations and Gut-Brain Axis." The authors have engaged constructively and in good faith with all major and minor comments raised in the review. I am pleased to recommend acceptance for publication.

Response:

We thank the reviewer for recommending acceptance.
