# Supplementary material for: Multilayer brain network analysis in mice reveals ketamine-induced reorganization of brain- wide fluctuations and gut-brain axis
Source: Commun Biol. 2026 Jul 3;9:1399. doi: 10.1038/s42003-026-10606-0 (PMC13696714; doi:10.1038/s42003-026-10606-0)
Supplement: Supplementary file 2 [file 42003_2026_10606_MOESM2_ESM.pdf]

**Multilayer Brain Network Analysis Reveals Ketamine-Induced Reorganization of Brain-wide Fluctuations and Gut-Brain Axis in mice**

*Fengkai He, Xiaojun Xu, Yuqi zhu, Jiahui Lu, Jiahui Lu, Dongyong Guo, Aili Liu, Wenwen Bai, Xuyuan Zheng, Baolin Guo, Tiaotiao Liu*[*]

## Supplemental Information:

### Supplemental Figures:

- Supplementary Figure 1. Multi-region electrode array for in vivo neural recordings.
- Supplementary Figure 2.The behavioral results of social interaction test
- Supplementary Figure 3. Edge density analysis
- Supplementary Figure 4. Edge density-matched global topology analysis
- Supplementary Figure 5. Correlations analysis between global topological properties and the social index (SI) for Con and CSDS group.
- Supplementary Figure 6. Animal-level classification performance based on FMBFN features.
- Supplementary Figure 7. Gut microbiota composition at different taxonomic levels.
- Supplementary Figure 8. identifying microbial biomarkers of ketamine's treatment at genus level.

### Supplemental Tables:

- Supplementary Table 1. List of abbreviations
- Supplementary Table 2. Sample sizes, primary endpoints, and sensitivity analyses for each experimental module
- Supplementary Table 3. Mediation analysis with FDR correction

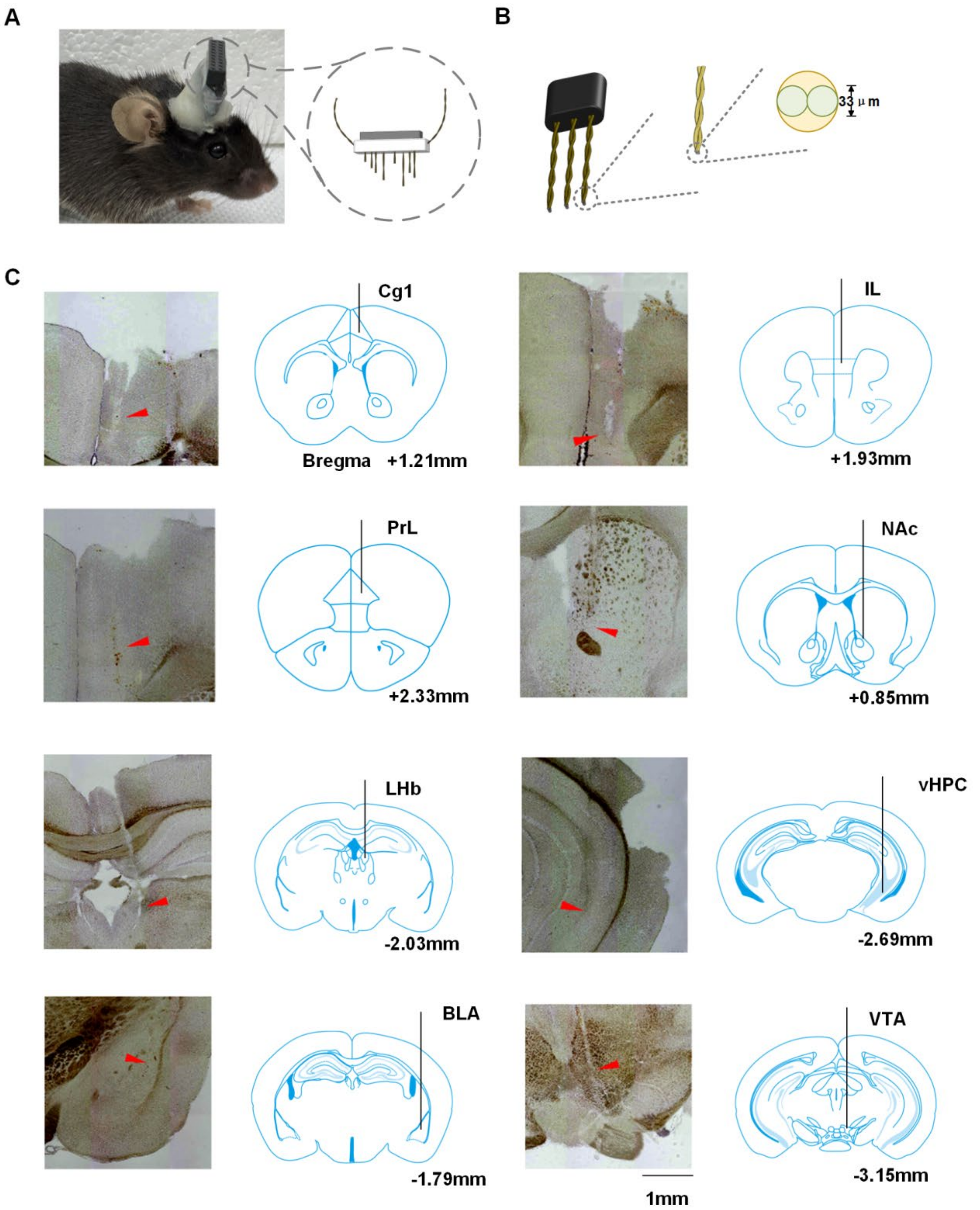


**Supplementary Figure 1. Multi-region electrode array for in vivo neural recordings.** (A) Photograph and illustration of chronically implanted device in mouse. (B) Schematic drawing of electrodes and wires arrangements. (C) Location of the electrode for eight brain regions.

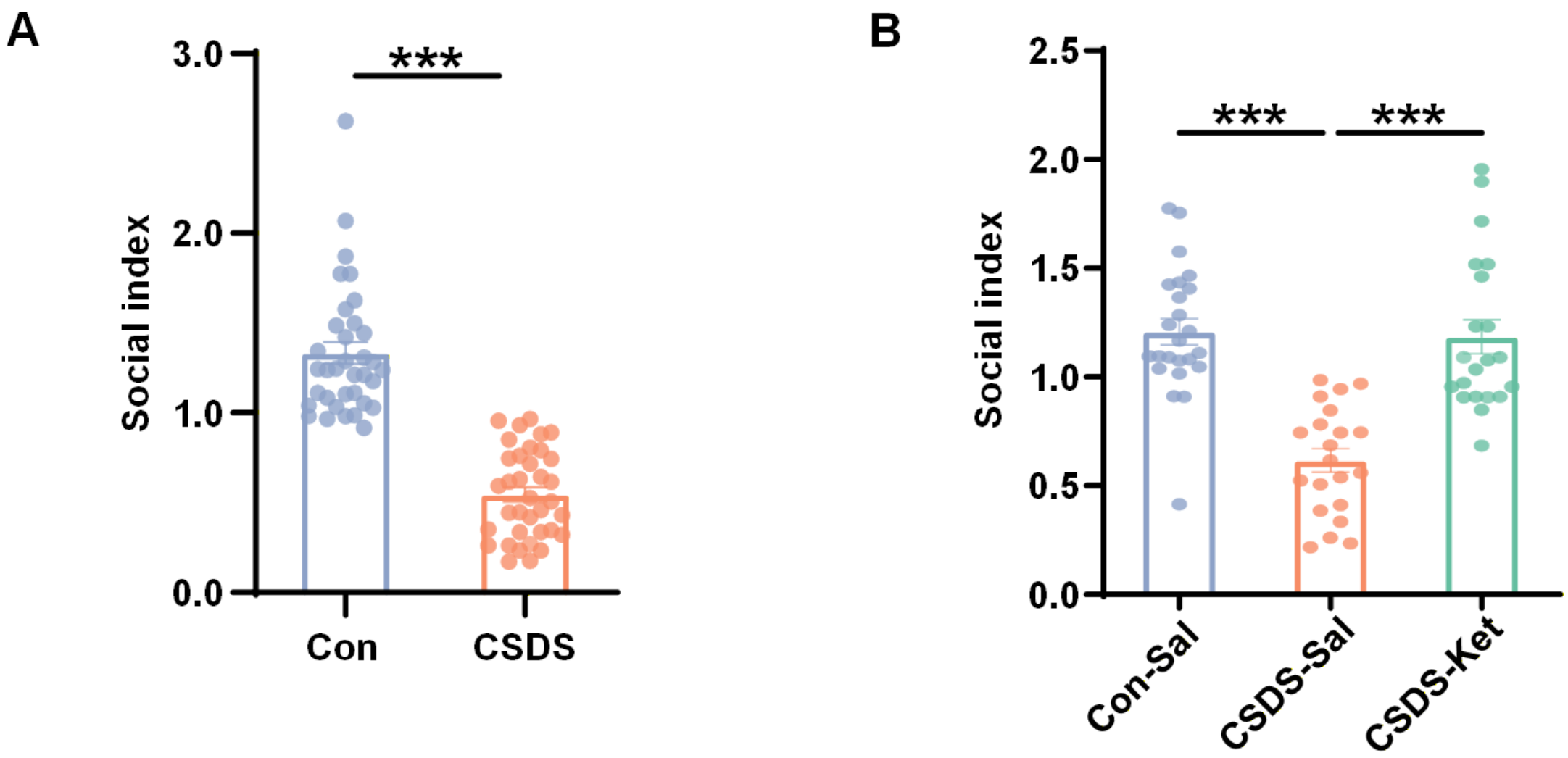


**Supplementary Figure 2. The behavioral results of social interaction test.** Behavioral performance of all mice assigned to the electrophysiological study is shown here prior to final inclusion in the network analyses. These data provide the complete behavioral context for the electrophysiological cohort. (A) Behavioral data from the full cohort of mice used for the electrophysiological comparison between the Con and CSDS groups (Unpaired t test; Con: 1.33 ± 0.060; CSDS: 0.55 ± 0.041; t(69)=10.89; Cohen's d=0.80 *** P < 0.001). (B) Behavioral data from the full cohort of mice used for the electrophysiological comparison among the Con-Sal, CSDS-Sal, and CSDS-Ket groups (Ordinary one-way ANOVA with Tukey's multiple comparisons test; Con-Sal: 1.21 ± 0.06, CSDS-Sal: 0.62 ± 0.05, CSDS-Ket: 1.18 ± 0.08; F (2, 15) = 26.42, $^{***}$ P < 0.001. Con-Sal vs CSDS-Sal: $^{***}$ P < 0.001, Con-Sal vs CSDS-Ket: $^{ns}$ P =0.96, CSDS-Sal vs CSDS-Ket: $^{**}$P < 0.01). (A-B) All data are presented as mean ± SEM.

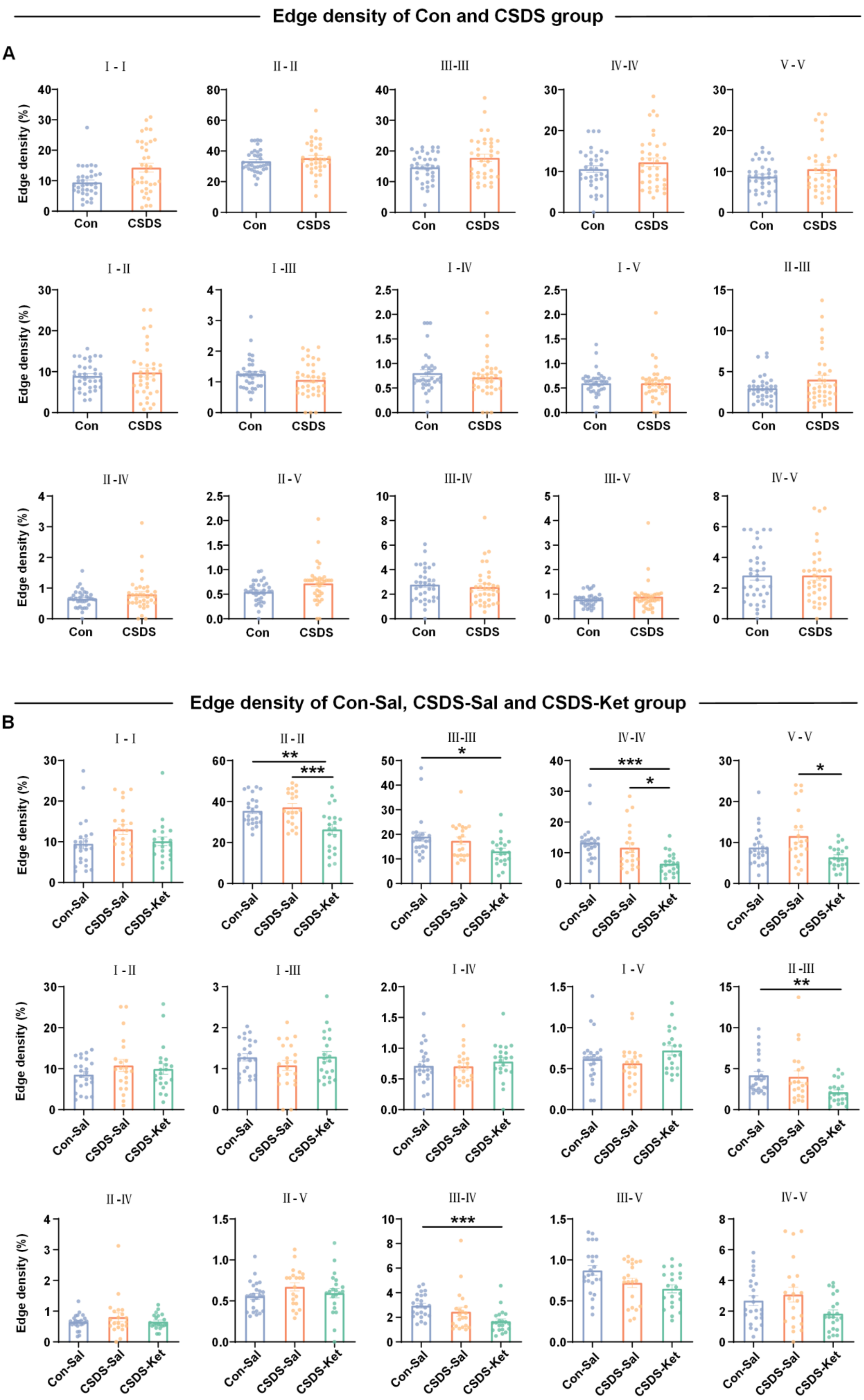
Edge density of Con and CSDS group
A
Ⅰ-Ⅰ
Ⅱ-Ⅱ
Ⅲ-Ⅲ
Ⅳ-Ⅳ
Ⅴ-Ⅴ
Ⅰ-Ⅱ
Ⅰ-Ⅲ
Ⅰ-Ⅳ
Ⅰ-Ⅴ
Ⅱ-Ⅲ
Ⅱ-Ⅳ
Ⅱ-Ⅴ
Ⅲ-Ⅳ
Ⅲ-Ⅴ
Ⅳ-Ⅴ
Edge density (%)
Con
CSDS
Edge density of Con-Sal, CSDS-Sal and CSDS-Ket group
B
Ⅰ-Ⅰ
Ⅱ-Ⅱ
Ⅲ-Ⅲ
Ⅳ-Ⅳ
Ⅴ-Ⅴ
Ⅰ-Ⅱ
Ⅰ-Ⅲ
Ⅰ-Ⅳ
Ⅰ-Ⅴ
Ⅱ-Ⅲ
Ⅱ-Ⅳ
Ⅱ-Ⅴ
Ⅲ-Ⅳ
Ⅲ-Ⅴ
Ⅳ-Ⅴ
Edge density (%)
Con-Sal
CSDS-Sal
CSDS-Ket

**Supplementary Figure 3. Edge density analysis.** (A) Edge density analysis for Con and CSDS group. (B) Edge density analysis for Con-Sal, CSDS-Sal and CSDS-Ket group. (A-B) Animal-level binary classification performance. Data are presented as mean ± SEM. All P values were corrected for multiple testing using the Benjamini–Hochberg false discovery rate (FDR) procedure. Detailed statistical methods and full statistical results are provided in Supplementary Data.

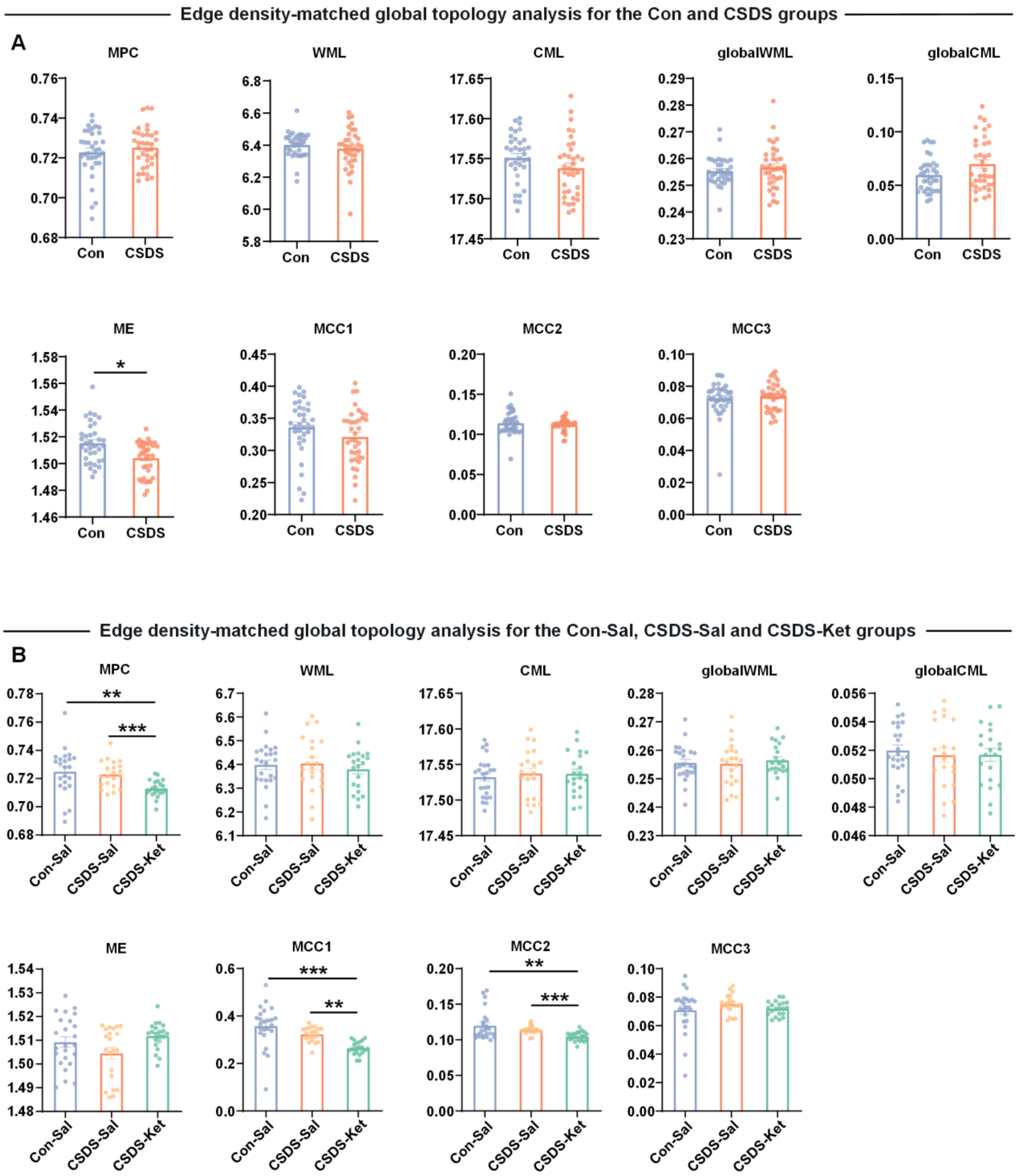


**Supplementary Figure 4. Edge density-matched global topology analysis.** (A) Edge density-matched global topology analysis for Con and CSDS group. (B) Edge density-matched global topology analysis for Con-Sal, CSDS-Sal and CSDS-Ket group. (A-B) For each comparison, binary networks were reconstructed using block-wise matched edge densities. Specifically, a common density for each within-frequency and cross-frequency block was defined from the observed densities of the groups being compared (implemented as the mean block-wise edge density across groups), and the strongest weighted edges were retained up to that density. This procedure preserved block-specific sparsity structure while minimizing the confounding effect of between-group edge-density differences. Animal-level binary

classification performance. Data are presented as mean ± SEM. All P values were corrected for multiple testing using the Benjamini–Hochberg false discovery rate (FDR) procedure. Detailed statistical methods and full statistical results are provided in Supplementary Data.

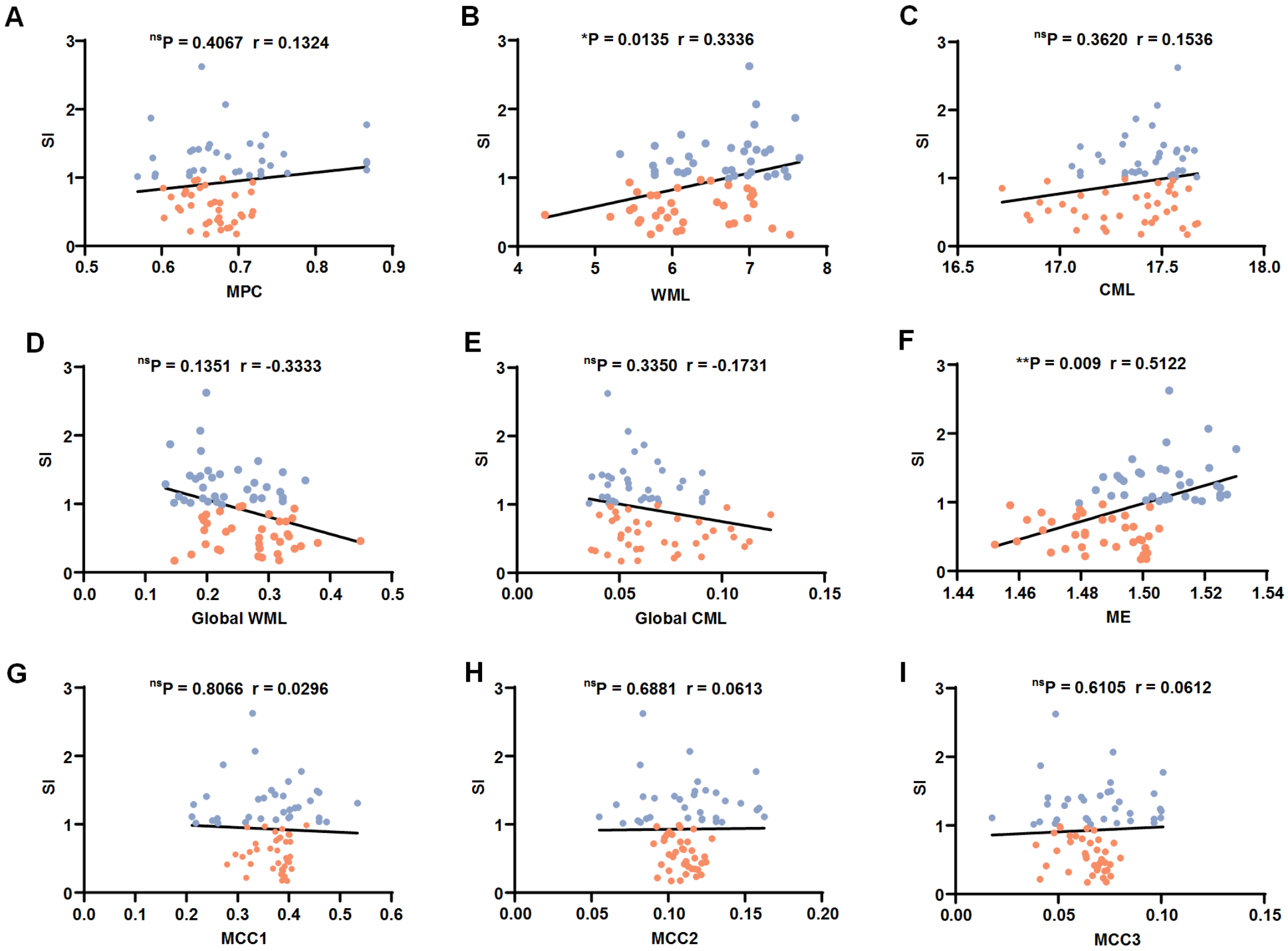


**Supplementary Figure 5. Correlations analysis between global topological properties and the social index (SI) for Con and CSDS group.** (A-I) Spearman correlation analysis between global topological properties and SI Each data point represents the average value of all epochs for a single mouse. For Con group: n= 35 mice; For CSDS group: n = 36 mice. All P values are FDR-corrected. All P values were corrected for multiple testing using the Benjamini–Hochberg false discovery rate (FDR) procedure. Detailed statistical methods and full statistical results are provided in Supplementary Data.

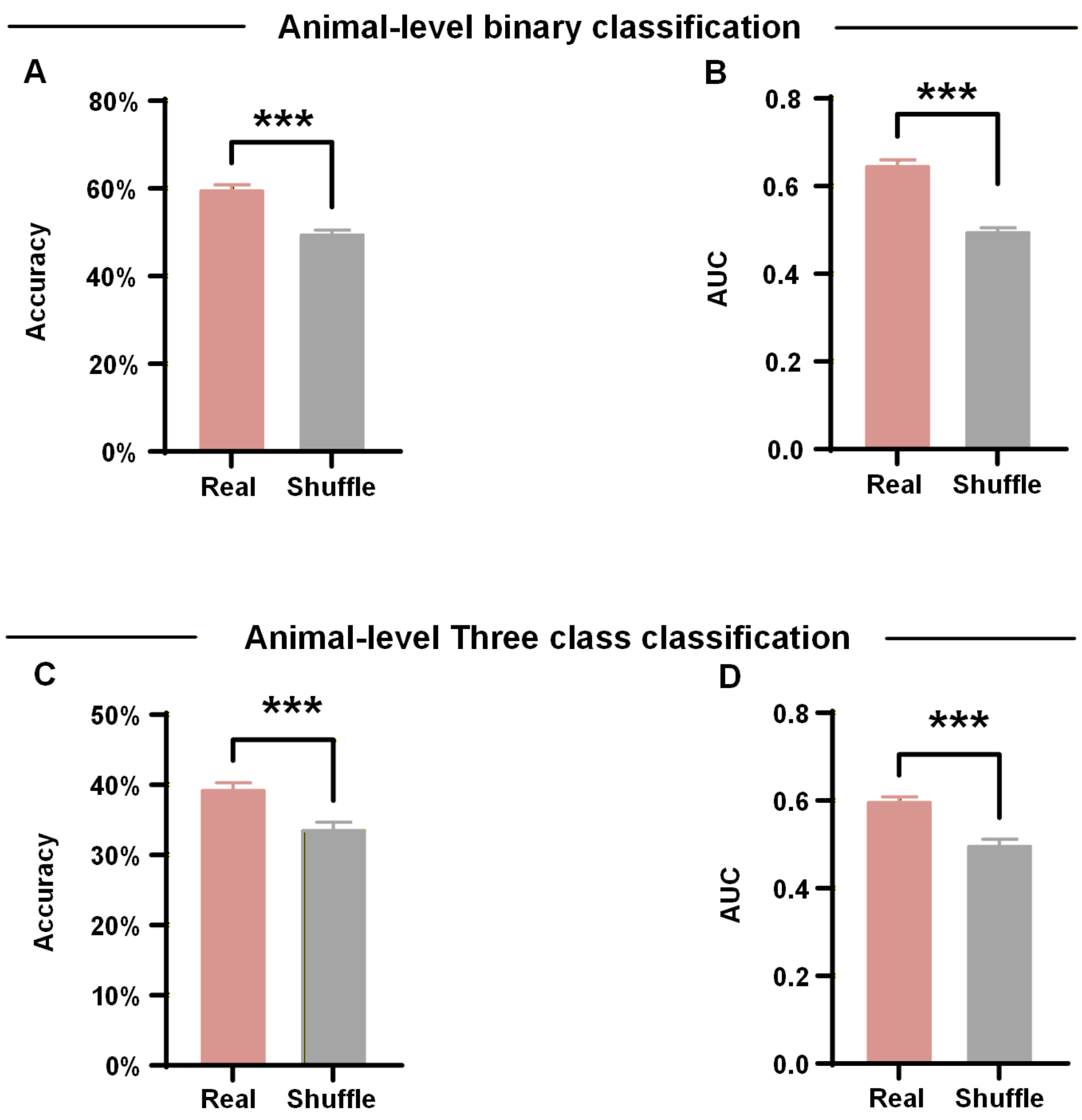


**Supplementary Figure 6. Animal-level classification performance based on FMBFN features.** (A) Binary classification accuracy for Real and Shuffle data. (Mann-Whitney test; Real 60.05% ± 1.08%; Shuffle: 50.00% ± 1.05%; U=2345.00; rank-biserial correlation (r_rb)=0.53 *** P < 0.001) (B) Binary classification AUC for Real and Shuffle data. (Mann-Whitney test; Real 0.67 ± 0.01; Shuffle: 0.50 ± 0.02; U=1930.00; rank-biserial correlation (r_rb)=0.61 *** P < 0.001). (A-B) Animal-level binary classification performance for the Con and CSDS group.Data are presented as mean ± SEM.

(C) Three-class classification accuracy for Real and Shuffle data. (Mann-Whitney test; Real 39.58% ± 0.94%; Shuffle: 33.84% ± 0.98%; U=3395.00; rank-biserial correlation (r_rb)=0.32 *** P < 0.001) (D) Three-class classification AUC for Real and Shuffle data. (Mann-Whitney test; Real 0.60 ± 0.01; Shuffle: 0.50 ± 0.02; U=2550.00; rank-biserial correlation (r_rb)=0.49 *** P < 0.001). (C-D) Animal-level three class classification performance for the Con-Sal、CSDS-Sal and CSDS-Ket group.Data are presented as mean ± SEM.

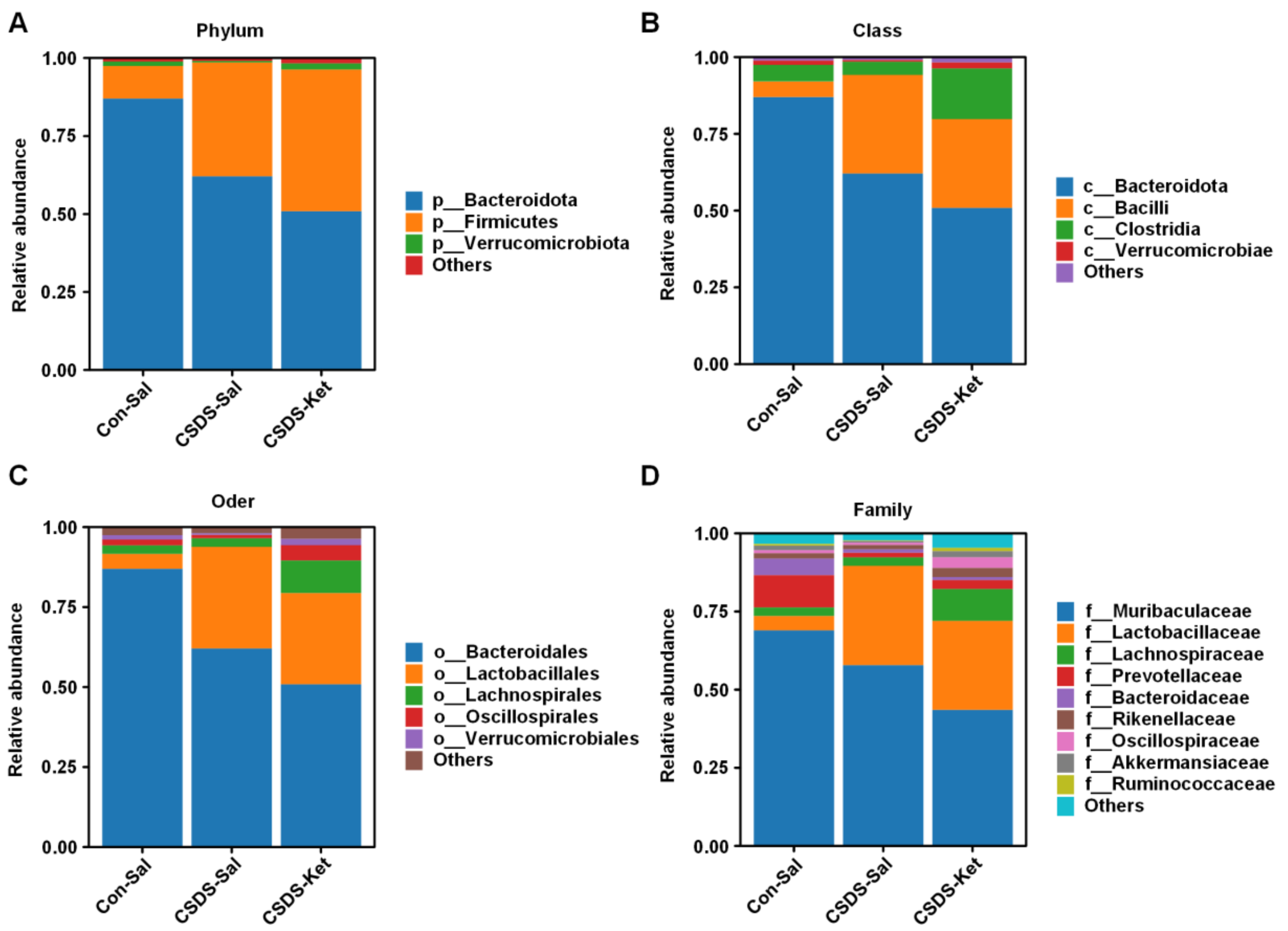


**Supplementary Figure 7. Gut microbiota composition at different taxonomic levels.** (A) Community bar plot at the phylum level. (B) Community bar plot at the class level. (C) Community bar plot at the order level. (D) Community bar plot at the family level.

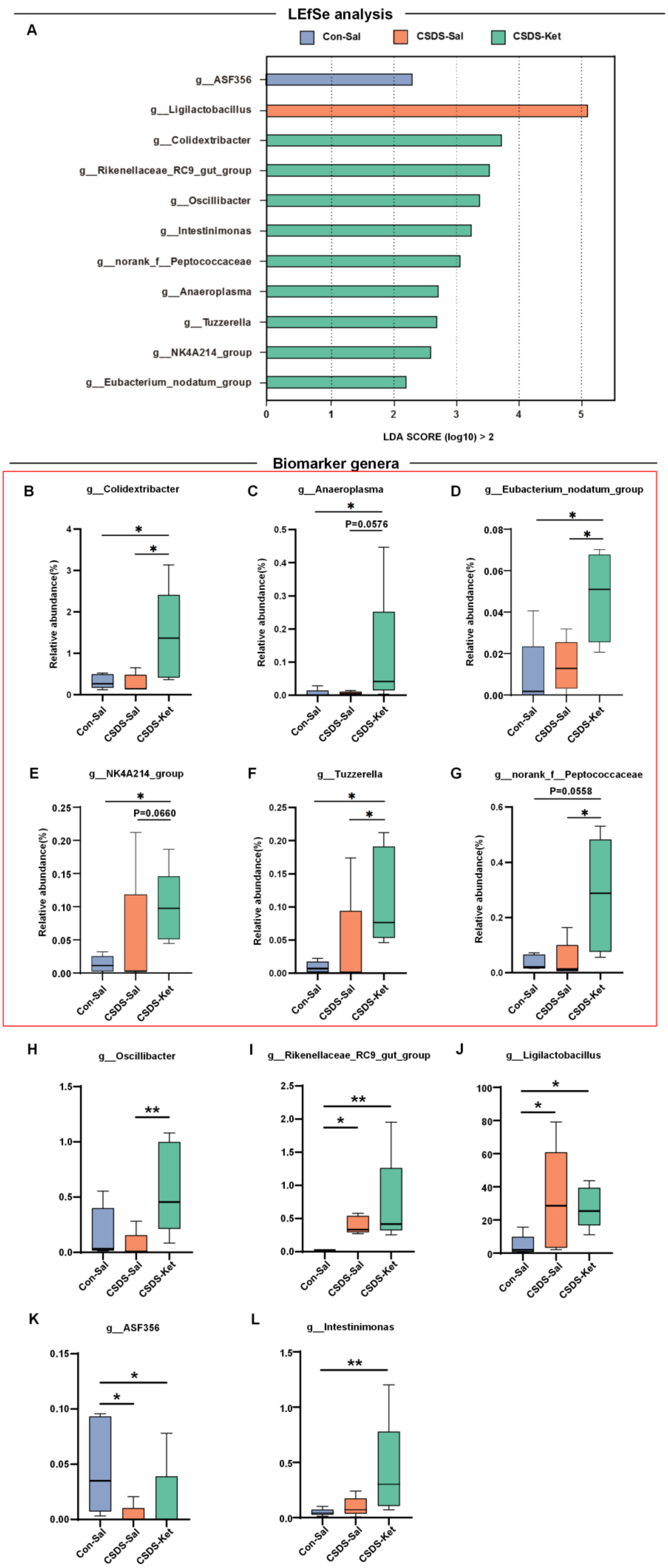

LEfSe analysis
A
Con-Sal
CSDS-Sal
CSDS-Ket
g__ASF356
g__Ligilactobacillus
g__Colidextribacter
g__Rikenellaceae_RC9_gut_group
g__Oscillibacter
g__Intestinimonas
g__norank_f__Peptococcaceae
g__Anaeroplasma
g__Tuzzerella
g__NK4A214_group
g__Eubacterium_nodatum_group
LDA SCORE (log10) > 2
Biomarker genera
B
g__Colidextribacter
C
g__Anaeroplasma
P=0.0576
D
g__Eubacterium_nodatum_group
E
g__NK4A214_group
P=0.0660
F
g__Tuzzerella
G
g__norank_f__Peptococcaceae
P=0.0558
H
g__Oscillibacter
I
g__Rikenellaceae_RC9_gut_group
J
g__Ligilactobacillus
K
g__ASF356
L
g__Intestinimonas
Relative abundance(%)
Con-Sal
CSDS-Sal
CSDS-Ket

**Supplementary Figure 8. identifying microbial biomarkers of ketamine's treatment at genus level.** (A) LEfSe analysis at the genus level. The bars represent the effect size (LDA score) of significantly discriminative genera. Colors indicate the group where each genus was most enriched (Con-Sal: blue; CSDS-Sal: orange; CSDS-Ket: green). (B-L) Kruskal-Wallis test for potential biomarkers Data are consistently expressed as Box plots (median ± IQR). For Con-Sal group: n= 5 mice; for CSDS-Sal group: n= 5 mice; for CSDS-Ket group: n = 5 mice. Detailed statistical methods and full statistical results are provided in Supplementary Data.

**Supplementary Table 1. List of abbreviations**

| Abbreviation | Full Name |
|---|---|
| ACC | Anterior cingulate cortex |
| AUC | Area under the curve |
| BLA | Basolateral amygdala |
| CFC | cross-frequency functional connectivity |
| CMDegree | Cross-frequency multilayer degree |
| CML | Cross-frequency multilayer characteristic path length |
| Cg1 | Cingulate cortex area 1 |
| CSDS | Chronic social defeat stress |
| FMBFN | Frequency-varying multilayer brain functional network |
| GFC | Generalized functional connectivity matrix |
| globalCML | Cross-frequency multilayer global efficiency |
| globalWML | Within-frequency multilayer global efficiency |
| HPA | Hypothalamic pitutary adrenal |
| IL | Infralimbic cortex |
| LFP | Local field potential |
| LHb | Lateral habenular nucleus |
| lMCC1 | Local multilayer clustering coefficient |
| lMCC2 | Local multilayer cross-two-frequencies clustering coefficient |
| lMCC3 | Local multilayer cross-three-frequencies clustering coefficient |
| lMPC | Local multilayer participation coefficient |
| MCC1 | Multilayer clustering coefficient |
| MCC2 | Multilayer cross-two-frequencies clustering coefficient |
| MCC3 | Multilayer cross-three-frequencies clustering coefficient |
| MPC | Global multilayer participation coefficient |
| ME | Multilayer entropy |
| NAc | Nucleus accumbens |
| PLI | phase-lag index |
| SIT | Social interaction test |
| SI | Social index |
| VTA | Ventral tegmental area |
| vHPC | ventral hippocampus |
| WFC | within-frequency functional connectivity |
| WMDegree | Within-frequency multilayer degree |
| WML | Within-frequency multilayer characteristic path length |

**Supplementary Table 2. Sample sizes, primary endpoints, and sensitivity analyses for each experimental module**

| Experimental module | Included analyses (figure panels) | Sample size | Primary endpoint | Statistical test for sensitivity-analysis | minimum detectable effect size | Alpha | Target power |
|---|---|---|---|---|---|---|---|
| CSDS behavioral validation | Time spent in the interaction zone during the "No CD1" phases of the SIT (Fig2B) | Con:n=6; CSDS:n=6 | Social interaction index (SI) | Two-tailed t test | minimum detectable effect size:d = 1.8 | 0.05 | 0.80 |
| | Time spent in the interaction zone during the "With CD1" phases of the SIT (Fig2C) | Con:n=6; CSDS:n=6 | | | | | |
| | social index of Con and CSDS mice (Fig2D) | Con:n=6; CSDS:n=6 | | | | | |
| | Velocity of Con and CSDS mice in SIT (Fig2E) | Con:n=6; CSDS:n=6 | | | | | |
| Ketamine behavioral validation | Time spent in the interaction zone during the "No CD1" phases of the SIT for each group (Fig4B) | Con-Sal:n=6; CSDS-Sal:n=6; CSDS-Ket:n=6 | Social interaction index (SI) | One-way ANOVA | minimum detectable effect size:d = 1.8 | | |
| | Time spent in the interaction zone during the "With CD1" phases of the SIT for each group (Fig4C) | Con-Sal:n=6; CSDS-Sal:n=6; CSDS-Ket:n=6 | | | | | |
| | SI of each group (Fig4D) | Con-Sal:n=6; CSDS-Sal:n=6; CSDS-Ket:n=6 | | | | | |
| | Velocity of each group in SIT (Fig4E) | Con-Sal:n=6; CSDS-Sal:n=6; CSDS-Ket:n=6 | | | | | |
| CSDS-related FMBFN | Statistical comparison of functional connectivity strength between Con and CSDS groups (Fig2H) | Con: n=35 ; CSDS: n=36 | Multilayer participatio | Two-tailed t test | minimum detectable | | |

| | | | | | | | |
|---|---|---|---|---|---|---|---|
| disruption | Comparison of the global toplogical properties between the Con and CSDS groups (Fig2I-2Q) | Con: n=35 ; CSDS: n=36 | n coefficient (MPC) AND Multilayer clustering coefficient (MCC1) | | effect size: d = 0.61 | | |
| | Region-specific comparisons of local multilayer topological properties between Con and CSDS groups (Fig3A) | Con: n=35 ; CSDS: n=36 | | | | | |
| | Spearman correlation analysis between global toplogical properties and SI (Fig S1A-I) | Con: n=35 ; CSDS: n=36 | | | | | |
| Ketamine-related FMBFN reconfiguration | Functional connectivity strength differences between CSDS-Sal and CSDS-Ket groups (Fig4F) | CSDS-Sal: n=21; CSDS-Ket: n=21 | Multilayer participation coefficient (MPC) AND Multilayer clustering coefficient (MCC1) | One-way ANOVA | minimum detectable effect size: f = 0.39 | | |
| | Functional connectivity strength differences between Con-Sal and CSDS-Ket groups (Fig4G) | Con-Sal: n=24; CSDS-Ket: n=21 | | | | | |
| | Comparison of the global toplogical properties among the Con-Sal, CSDS-Sal and CSDS-Ket groups (Fig4H-3P) | Con-Sal: n=24; CSDS-Sal: n=21; CSDS-Ket: n=21 | | | | | |
| | Region-specific comparisons of local multilayer topological properties between CSDS-Sal and CSDS-Ket groups (Fig5A) | CSDS-Sal: n=21; CSDS-Ket: n=21 | | | | | |
| | Region-specific comparisons of local multilayer topological properties between Con-Sal and CSDS-Ket groups (Fig5B) | Con-Sal: n=24; CSDS-Ket: n=21 | | | | | |
| Gut–brain multimodal integration | Comparison of microbial alpha-diversity (Fig6B) | Con-Sal: n=5; CSDS-Sal: n=5; CSDS-Ket: n=5 | Correlation between microbial features and | spearman correlation test | Minimum detectable correlation :\|ρ\| =0.65 | | |
| | Beta diversity analysis of gut microbiota (Fig6C-6D) | Con-Sal: n=5; CSDS-Sal: n=5; | | | | | |

| | | | | | | | |
|---|---|---|---|---|---|---|---|
| | | CSDS-Ket: n=5 | global topological properties | | | | |
| | Gut microbiota composition at different taxonomic levels (Fig S3A-3D) | Con-Sal: n=5; CSDS-Sal: n=5; CSDS-Ket: n=5 | | | | | |
| | LEfSe analysis at the genus level (Fig S4A) | Con-Sal: n=5; CSDS-Sal: n=5; CSDS-Ket: n=5 | | | | | |
| | Relative abundance of ketamine-associated biomarker candidates (Fig S5B-5L) | Con-Sal: n=5; CSDS-Sal: n=5; CSDS-Ket: n=5 | | | | | |
| | Correlation between microbial features and global topological properties (Fig 6E) | Con-Sal: n=5; CSDS-Sal: n=5; CSDS-Ket: n=5 | | | | | |
| | Microbiota-mediated effects: Ketamine's effects on network properties mediated through gut microbiota (Fig 6F-6M) | CSDS-Sal: n=5; CSDS-Ket: n =5 | | | | | |

Note: Sensitivity analyses were performed on the basis of the pre-specified primary endpoint for each experimental module, using the achieved sample size corresponding to that primary endpoint rather than every individual test within the module.

**Supplementary Table 3. Mediation analysis with FDR correction**

| Direction | Predictor (X) | Mediator (M) | Outcome (Y) | Indirect effect (ab) | 95% bootstrap CI | Nominal p value | FDR-adjusted q value | Is mediation effect significant | Sample size (n) | FDR family |
|---|---|---|---|---|---|---|---|---|---|---|
| Microbiota-mediated | Ketamine treatment | g_Colidextribacter | MPC | -0.4271 | [-0.9432, -0.0883] | 0.0052 | 0.0416 | Yes | CSDS-Sal: n=5; CSDS-Ket :n=5 | Family M1: microbiota-mediated indirect effects (8 tests) |
| | | g_NK4A214_group | MPC | -0.0172 | [-0.1308, 0.0245] | 0.3392 | 0.4523 | No | | |
| | | g_NK4A214_group | MCC2 | -0.0042 | [-0.0283, 0.0135] | 0.4528 | 0.4528 | No | | |
| | | g_NK4A214_group | MCC3 | -0.0051 | [-0.0147, 0.0125] | 0.4528 | 0.4528 | No | | |
| | | g_Tuzzerella | MPC | -0.0236 | [-0.1284, 0.0048] | 0.1656 | 0.265 | No | | |
| | | g_norank_f_Peptococcaceae | MPC | -0.3564 | [-0.8411, -0.0203] | 0.0236 | 0.0472 | Yes | | |
| | | g_norank_f_Peptococcaceae | MCC2 | -0.3433 | [-0.8623, -0.0342] | 0.0132 | 0.0472 | Yes | | |
| | | g_norank_f_Peptococcaceae | MCC3 | -0.3661 | [-1.1113, -0.0853] | 0.0188 | 0.0472 | Yes | | |
| Network-mediated | | MPC | g_Colidextribacter | 1.0593 | [0.2533, 15462] | 0.0012 | 0.0096 | Yes | | Family M2: network-medi |

| | | | | | | | | | | |
|---|---|---|---|---|---|---|---|---|---|---|
| | | MPC | g_NK4A214_group | 0.1120 | [-0.0158, 0.3046] | 0.092 | 0.1269 | No | | ated indirect effects (8 tests) |
| | | MCC2 | g_NK4A214_group | 0.1152 | [-0.0670, 0.3355] | 0.2228 | 0.2228 | No | | |
| | | MCC3 | g_NK4A214_group | 0.1575 | [-0.1670, 0.2757] | 0.1888 | 0.2158 | No | | |
| | | MPC | g_Tuzzerella | 0.1051 | [-0.0161, 0.2277] | 0.0952 | 0.1269 | No | | |
| | | MPC | g_norank_f_Peptococcaceae | 0.7778 | [0.0762, 1.7774] | 0.0203 | 0.0496 | Yes | | |
| | | MCC2 | g_norank_f_Peptococcaceae | 1.2071 | [0.2353, 1.9761] | 0.0248 | 0.0496 | Yes | | |
| | | MCC3 | g_norank_f_Peptococcaceae | 1.0134 | [0.3432, 2.9364] | 0.008 | 0.032 | Yes | | |

Note: MPC, Global multilayer participation coefficient; MCC2, Cross-two-frequency clustering coefficient; MCC3: Cross-three-frequency clustering coefficient

Benjamini–Hochberg FDR correction was applied separately to two pre-specified mediation families: microbiota-mediated models and network-mediated models.
