# Supplementary material for: Multilayer brain network analysis in mice reveals ketamine-induced reorganization of brain- wide fluctuations and gut-brain axis
Source: Commun Biol. 2026 Jul 3;9:1399. doi: 10.1038/s42003-026-10606-0 (PMC13696714; doi:10.1038/s42003-026-10606-0)
Supplement: Supplementary file 3 [file 42003_2026_10606_MOESM3_ESM.pdf]

Description of Additional Supplementary Files

File name: Supplementary Data 1

Description: The Summary of statistical analyses in the paper.
