# Supplementary material for: Multilayer brain network analysis in mice reveals ketamine-induced reorganization of brain- wide fluctuations and gut-brain axis
Source: Commun Biol. 2026 Jul 3;9:1399. doi: 10.1038/s42003-026-10606-0 (PMC13696714; doi:10.1038/s42003-026-10606-0)
Supplement: Supplementary file 4 [file 42003_2026_10606_MOESM5_ESM.pdf]

nature portfolio

Corresponding author(s): COMMSBIO-23-10210B (Tiaotiao Liu)

Last updated by author(s): Jun 15, 2026

Reporting Summary

Nature Portfolio wishes to improve the reproducibility of the work that we publish. This form provides structure for consistency and transparency in reporting. For further information on Nature Portfolio policies, see our Editorial Policies and the Editorial Policy Checklist.

Statistics

For all statistical analyses, confirm that the following items are present in the figure legend, table legend, main text, or Methods section.

n/a	Confirmed	
☐	☒	The exact sample size (*n*) for each experimental group/condition, given as a discrete number and unit of measurement
☐	☒	A statement on whether measurements were taken from distinct samples or whether the same sample was measured repeatedly
☐	☒	The statistical test(s) used AND whether they are one- or two-sided<br>*Only common tests should be described solely by name; describe more complex techniques in the Methods section.*
☐	☒	A description of all covariates tested
☐	☒	A description of any assumptions or corrections, such as tests of normality and adjustment for multiple comparisons
☐	☒	A full description of the statistical parameters including central tendency (e.g. means) or other basic estimates (e.g. regression coefficient) AND variation (e.g. standard deviation) or associated estimates of uncertainty (e.g. confidence intervals)
☐	☒	For null hypothesis testing, the test statistic (e.g. *F*, *t*, *r*) with confidence intervals, effect sizes, degrees of freedom and *P* value noted<br>*Give P values as exact values whenever suitable.*
☒	☐	For Bayesian analysis, information on the choice of priors and Markov chain Monte Carlo settings
☐	☒	For hierarchical and complex designs, identification of the appropriate level for tests and full reporting of outcomes
☐	☒	Estimates of effect sizes (e.g. Cohen's *d*, Pearson's *r*), indicating how they were calculated

Our web collection on statistics for biologists contains articles on many of the points above.

Software and code

Policy information about availability of computer code

Data collection	In vivo electrophysiological recordings from multiple brain regions were performed using the Cerebus-128 multichannel neural signal acquisition and analysis system(Blackrock Microsystems,USA). Animal behavior was recorded simultaneously using EthoVision XT (Version 8.5).
Data analysis	Data was analyzed using EEGLAB (Version 2023.1), Chronux toolbox (Version 2.12), Brain Connectivity Toolbox (Version 2019_03_03) and MATLAB (R2023a). Custom MATLAB scripts in this study is available on Figshare.com (https://doi.org/10.6084/m9.figshare.30475247).

For manuscripts utilizing custom algorithms or software that are central to the research but not yet described in published literature, software must be made available to editors and reviewers. We strongly encourage code deposition in a community repository (e.g. GitHub). See the Nature Portfolio guidelines for submitting code & software for further information.

Data

Policy information about availability of data

All manuscripts must include a data availability statement. This statement should provide the following information, where applicable:

- Accession codes, unique identifiers, or web links for publicly available datasets
- A description of any restrictions on data availability
- For clinical datasets or third party data, please ensure that the statement adheres to our policy

The dataset generated and analyzed during the current study is available on Figshare.com at the following link: https://doi.org/10.6084/m9.figshare.30472571.

Research involving human participants, their data, or biological material

Policy information about studies with human participants or human data. See also policy information about sex, gender (identity/presentation), and sexual orientation and race, ethnicity and racism.

Reporting on sex and gender	The study did not involve human participants.
Reporting on race, ethnicity, or other socially relevant groupings	The study did not involve human participants.
Population characteristics	The study did not involve human participants.
Recruitment	The study did not involve human participants.
Ethics oversight	The study did not involve human participants.

Note that full information on the approval of the study protocol must also be provided in the manuscript.

Field-specific reporting

Please select the one below that is the best fit for your research. If you are not sure, read the appropriate sections before making your selection.

☒ Life sciences ☐ Behavioural & social sciences ☐ Ecological, evolutionary & environmental sciences

For a reference copy of the document with all sections, see nature.com/documents/nr-reporting-summary-flat.pdf

Life sciences study design

All studies must disclose on these points even when the disclosure is negative.

Sample size	Required sample sizes were estimated on the basis of published studies, although no statistical methods were used to predetermine sample sizes. Mice were randomly assigned to treatment groups.
Data exclusions	Base on pre-established criteria, values were excluded from analysis if electrode implant sites were out of the targeted eight regions.
Replication	The repeatability of all key findings in this study was rigorously ensured through a multi-faceted strategy centered on biological replication and statistical validation. Specifically, all experiments were designed with sufficient biological replicates (e.g., n=35-36 mice per group across independent cohorts) to account for individual variability.<br>We used independent samples across different batches under consistent experimental conditions.<br>Statistical significance (e.g., p<0.05) served as aquantitative measure of reliability, confirming that observed effects were unlikely to arise by chance.
Randomization	Subjects were randomly assigned to experimental groups using a computer-generated randomization sequence to ensure baseline equivalence and minimize selection bias. Upon acclimatization, mice were first allovated to control or chronic social defeat stress (CSDS) groups. After CSDS induction, stress-induced social avoidant mice were further randomized to receive either saline or ketamine treatment. Blinded implementation was maintained during assignments.
Blinding	Analyses were performed in a manner blinded to treatment assignments in all behavioral experiments.

Reporting for specific materials, systems and methods

We require information from authors about some types of materials, experimental systems and methods used in many studies. Here, indicate whether each material, system or method listed is relevant to your study. If you are not sure if a list item applies to your research, read the appropriate section before selecting a response.

Materials & experimental systems

n/a	Involved in the study	
☒	☐	Antibodies
☒	☐	Eukaryotic cell lines
☒	☐	Palaeontology and archaeology
☐	☒	Animals and other organisms
☒	☐	Clinical data
☒	☐	Dual use research of concern
☒	☐	Plants

Methods

n/a	Involved in the study	
☒	☐	ChIP-seq
☒	☐	Flow cytometry
☒	☐	MRI-based neuroimaging

Animals and other research organisms

Policy information about studies involving animals; ARRIVE guidelines recommended for reporting animal research, and Sex and Gender in Research

Laboratory animals	The study used the following laboratory animals: Male CD-1 retired breeder mice (aged 4-6 months), male adult C57BL/6 mice (aged 8-12 weeks).
Wild animals	The study did not involve wild animals.
Reporting on sex	All experiments utilized male mice exclusively, as required by the Chronic social defeat stress (CSDS)paradigm and social interaction test (SIT), As CSDS relies on innate male aggression for induction, and SIT uses male CD-1 mice as social targets.
Field-collected samples	The study did not involve wild animals.
Ethics oversight	All experimental procedures were conducted with approved protocols from the care and use of laboratory animals and the Tianjin Medical University animal care and use committee (No. TMUaMEC2021060).

Note that full information on the approval of the study protocol must also be provided in the manuscript.

Plants

Seed stocks	The study did not involve plants.
Novel plant genotypes	The study did not involve plants.
Authentication	The study did not involve plants.
